# Discovery of a novel Nrf2 activator that modulates mitochondrial function in neurons by regulating DHRS3-Nrf2 interaction after ischemic stroke

**DOI:** 10.7150/thno.128602

**Published:** 2026-03-30

**Authors:** Xiaohui Sun, Zhaofeng Liu, Huanhuan An, Hengwei Xu, Fangxia Zou, Jing Lu, Xiaofan Zhang, Xinyu Han, Ziwei Song, Yanli Sun, Wenyan Wang, Hongbo Wang, Jianzhao Zhang, Yunjie Wang, Jingwei Tian

**Affiliations:** 1 School of Pharmacy, Key Laboratory of Molecular Pharmacology and Drug Evaluation (Yantai University), Ministry of Education, Collaborative Innovation Center of Advanced Drug Delivery System and Biotech Drugs in Universities of Shandong, Yantai University, Yantai, 264005, China.; 2 State Key Laboratory of Advanced Drug Delivery and Release Systems, Shandong Luye Pharmaceutical Co., Ltd., Yantai, Shandong, 264003, China.; 3 School of Life Science and Technology, ShanghaiTech University, Shanghai, 201210, China.

**Keywords:** Nrf2 activator, acute ischemic stroke, DHRS3, Keap1, neurons, mitochondria

## Abstract

***Rationale:*** Given the crucial role of the Nrf2 pathway in cellular adaptability to stress, targeting small-molecule activation of Nrf2 represents a promising therapeutic strategy for acute ischemic stroke (AIS). However, the clinical translation of existing Nrf2 activators is hindered by adverse effects, such as liver damage, and none are currently approved for AIS. Therefore, we aimed to develop a novel Nrf2 activator that specifically activates neuronal Nrf2 while mitigating adverse effects, with the goal of providing a lead compound for AIS.

***Methods:*** We validated the anti-AIS effects and mitochondrial protective functions of the novel Nrf2 activator Cpd.51 through multiple *in vivo* and *in vitro* experiments. Mechanistic studies involving surface plasmon resonance, cellular thermal shift assay, co-immunoprecipitation, chromatin immunoprecipitation, GST pull-down, and RNA sequencing were used to determine how Cpd.51 activates Nrf2. A comparative toxicological evaluation was conducted to demonstrate its superior safety profile over parent compound (Omaveloxolone).

***Results:*** Cpd.51 exhibited favorable blood-brain barrier permeability, improved safety profile, enhanced mitochondrial function protection and significant neuroprotective effect through the specific activation of neuronal Nrf2. Mechanistically, Cpd.51 interacted with Cys151 and Gly148 in the BTB domain of Keap1, inhibiting Nrf2 degradation, consequently suppressing the transcription of its downstream target DHRS3, a member of the short-chain dehydrogenase/reductase family. Furthermore, Cpd.51 exerted additional Nrf2-activating activity by disrupting protein-protein interactions between Nrf2 and DHRS3.

***Conclusions:*** Our work identified Cpd.51 as a novel and safe Nrf2 activator and unveils a unique feedback mechanism involving Nrf2-DHRS3 interaction, providing a new therapeutic avenue for AIS.

## Introduction

Acute ischemic stroke (AIS) manifests abruptly with neurological deficits including hemiplegia, dysarthria, sensory disturbances, aphasia, and visual impairment, often culminating in mortality. This condition inflicts substantial psychological and socioeconomic burdens on patients and caregivers while straining global healthcare systems 1, 2. Despite advances in medical care, stroke remains the world's second-leading cause of death (responsible for 11.6% of total disease mortality) and the third-largest contributor to disability-adjusted life years (DALYs) at 5.7% of global DALYs 3. Apart from recombinant tissue-type plasminogen activator (rt-PA), there are no effective treatment options for AIS. However, its narrow therapeutic window and risk of hemorrhagic complications restrict clinical application, only ~13.5% of eligible patients receive thrombolysis 4. Consequently, identifying novel neuroprotective agents and therapeutic targets for AIS remains an urgent priority.

Nuclear factor erythroid 2-related factor 2 (Nrf2) serves as the master transcriptional regulator coordinating redox homeostasis, metabolic stability, proteostasis, and intracellular iron dynamics, collectively determining cellular adaptability to stress 5. These processes constitute key pathological mechanisms in cerebral ischemia/reperfusion (I/R) injury. Under ischemic conditions, reactive oxygen species (ROS) disrupt the cytoplasmic Nrf2-Keap1 (Kelch-like ECH-associated protein 1) complex, facilitating Nrf2 nuclear translocation, where it dimerizes with small MAF proteins (sMAF) and binds to antioxidant response elements (ARE). However, prolonged I/R paradoxically triggers Nrf2 degradation via ubiquitination or epigenetic silencing 6, 7. Preclinical evidence consistently demonstrates that pharmacological or genetic activation of the Nrf2 pathway mitigates AIS damage, whereas Nrf2 inhibition exacerbates injury 6-8. Clinically relevant Nrf2 inducers, including USA Food and Drug Administration (FDA) approves agents like dimethyl fumarate (DMF) for relapsing multiple sclerosis and Omaveloxolone (Oma) for Friedreich's ataxia. They have demonstrated multiple biological activities in clinical and preclinical studies: DMF modulates oxidative stress and immune responses, while Oma inhibits neuroinflammation and restores mitochondrial function 9-11. Given this mechanistic rationale, targeted Nrf2 activation represents a promising therapeutic strategy for AIS, warranting further translational research and drug development.

Dehydrogenase/reductase (SDR family) member 3 (DHRS3), a key metabolic enzyme within the short-chain dehydrogenase/reductase (SDR) superfamily, modulates retinoid flux, lipid homeostasis, and signaling cascades to influence diverse physiological and pathological processes 12, 13. DHRS3 reduces retinaldehyde to retinol, thereby blocking the synthesis pathway of retinoic acid (RA) 14. Critically, reduced serum RA levels correlate with increased mortality risk in first-time AIS patients 15. RA exerts neuroprotective effects by maintaining blood-brain barrier integrity, mitigating hemorrhagic transformation following rt-PA administration, and suppressing neuroinflammation through inhibition of neutrophil extracellular trap formation in ischemic lesions 16, 17. Consequently, pharmacological inhibition of DHRS3 represents a promising strategy to ameliorate AIS injury. Current research on DHRS3 primarily focuses on its roles in oncogenesis and inflammation. It functions as a tumor suppressor in papillary thyroid cancer, gastric cancer, and breast cancer, where its expression serves as a prognostic biomarker 13, 18, 19. In inflammatory contexts, overexpression of DHRS3 affects macrophage differentiation and regulates anti-inflammatory responses in Crohn's disease stromal cells 20, 21. Nevertheless, pathophysiological significance of DHRS3 in ischemic stroke and potential bidirectional regulation with Nrf2 remains underexplored, warranting further investigation into its potential as a novel target for modulating AIS progression.

DMF and Oma are cysteine-reactive compounds that target specific cysteine residues on Keap1, particularly Keap1 Cys151. These inducers covalently modify Keap1, leading to inhibition of the Keap1-Cullin3 E3 ubiquitin ligase and stabilization of Nrf2 5, 10, 22. Although these inducers are effective, they often exhibit off-target effects due to electrophilic reactions with other proteins containing reactive cysteine residues 23-26. To develop an Nrf2 activator with improved selectivity and safety, we synthesized a series of Oma derivatives through structural modification. Following extensive* in vitro* and *in vivo* evaluation, Compound 51 (Cpd.51) emerged as a candidate with enhanced bioactivity, reduced hepatotoxicity and favorable druggability. Basically, we first examined the capacity of Cpd.51 to activate Nrf2 using the ARE-Luciferase reporter gene system and a novel Nrf2 sensor. Additionally, Cpd.51 mitigated AIS pathology through its protective effects on mitochondria, which were mediated by neuron-specific activation of Nrf2, as demonstrated in both *in vitro* and *in vivo* pharmacodynamic studies. Next, we investigated the mechanisms by which Cpd.51 activated Nrf2. Thermodynamic and kinetic analyses revealed that Cpd.51 bound directly to the Gly148 and Cys151 residues of Keap1. However, surface plasmon resonance (SPR) results demonstrated that Cpd.51 exhibited a lower binding affinity for the BTB domain of Keap1 in comparison to Oma. This finding was in stark contrast to its potent Nrf2 activation and robust neuroprotective effects observed in AIS models. Further mechanistic studies revealed a dual regulatory axis: (1) Cpd.51 bound to DHRS3 at a site overlapping the Nrf2-DHRS3 binding interface, disrupting the binding of the protein-protein and thereby enhancing the Nrf2-mediated antioxidant response. (2) Nrf2 suppressed DHRS3 transcriptional activity, mitigating DHRS3-mediated neurotoxicity. Critically, this work elucidated novel Nrf2-DHRS3 crosstalk in AIS and identifies Cpd.51 as a promising therapeutic candidate.

## Materials and Methods

### Cell culture and drug administration *in vitro*

Human neuroblastoma cell line (SH-SY5Y, RRID: CVCL_0019), microglia cell line (HMC3, RRID: CVCL_II76), astrocyte cell line (TNA2, RRID: CVCL_3670) and Hek-293T (RRID: CVCL_ZK70) cells were purchased from American Type Culture Collection (ATCC, China), and all the cell lines were confirmed contamination free. SH-SY5Y were grown in MEM (12492013, Gibco, USA) supplemented with 10% FBS and 1% P/S and 1% NESS. Hek-293T were grown in DMEM (11965092, Gibco, USA) supplemented with 10% FBS and 1% P/S. TNA2 survived in MEM complete medium, which was purchased from Sunncell (SNLM-552, China). HMC3 survived in DMEM complete medium, which was purchased from Procell (CM-0620, China). All cells were grown at 37 °C in a humidified incubator in an atmosphere of 95% O_2_ and 5% CO_2_.

To mimic ischemic conditions* in vitro*, cells were washed with PBS and then cultured for 2.5 h at 37 °C in glucose-free medium under anaerobic conditions (5% hydrogen, 90% nitrogen, and 5% carbon dioxide). OGD process was terminated by transferring cells back to normal glucose-containing medium under normoxic conditions (5% CO₂, 37 °C) and maintained for 24 h.

In the administration group, cells were exposed for 12 h with Cpd.51 prior to OGD. OGD lasted for 2.5 h and the reperfusion lasted for 24 h before the experiment was concluded. Subsequently, cell viability was determined using the cell counting kit-8 (CCK-8, C0039, Beyotime, China). In the experiment to explore the treatment time window, the only difference was the timing of the Cpd.51 treatment, while all other procedures remained unchanged.

### Isolation and culture of primary cells

Rat primary neuronal cells collected from the cerebral cortices of embryos (embryonic day 18) from pregnant SD rats were isolated and cultured. In brief, the cerebral cortex tissue of fetal mice was removed and cut into small pieces using ophthalmic curved shears. The tissue was repeatedly pipetted after the addition of preheated 0.25% Trypsin digestive solution (BR00083, ABclonal, China). Finally, twice the volume of the culture medium was added and terminated the digestion to complete the extraction of primary cortical cells.

Primary neuronal cells were seeded in plates coated with poly-D-lysine (P2100, Solarbio, China), and maintained in serum-free neurobasal (10888022, Gibco, USA) with 1% Glutamax (35050061, Gibco, USA) and 1% B27 supplement (17504044, Gibco, USA) at 37 °C in a 5% CO_2_ incubator. Cytosine arabinoside (V900339, Sigma, USA) was added to prevent non-neuronal proliferation. Half of the medium was replaced every three days. After seven days of cell culture, the drugs were applied and subsequent experiments were conducted 34. At the same time, we ensured that the cells were not contaminated and growing well, as shown by immunofluorescence co-staining with β-tubulin and Synaptophysin.

### ATP test

Experiment one: to test the cell viability, the cells were inoculated into 96-well plates. After treatment, 100 μL of ATP detection solution (0RT0769, PerkinElmer, USA) was added to detect by luminomenter.

Experiment two: mitochondrial ATP levels were assayed using ATP assay kit (S0027, Beyotime, China) according to the manufacturer's protocols. The cells were cultured by placing them in six-well plates as described in the "Cell culture and drug administration *in vitro*" method. Subsequently, 200 uL of lysate was added into the hole and supernatants were collected (4 °C, 12000 g, 5 min). 100 uL supernatants cultured with 100 µL probe of ATP, and the RLU was measured by a luminometer.

### Luciferase reporter assays

The Hek-293T cells were cultured in 96-well plates 24 h before transfection with ARE-luciferase reporter plasmid (11548ES03, Yeasen, China) for 48 h according to the manufacturer's instructions. The Firefly luciferase activities was carried out 12 h after the drug treatment, as described by the manufacturer (11412ES81, Yeasen, China).

### Novel biosensor screens for activators of Nrf2

Nrf2-Keap1 separation sensor was commissioned to be produced by Professor Zhang Qiang from Zhejiang University. The design principle of it is to attach the HOTag small peptide to Nrf2-GFP and co-express it with Keap1-mCherry. Due to the oligomerization effect of HOTag, a polymeric structure - "phase transition" droplet - of Nrf2-GFP-HOTag/Keap1-mCherry was formed, thereby inhibiting the degradation of Nrf2 by the proteasome and increasing the fluorescence intensity by more than 1000 times. The signal was captured and analyzed by high-throughput imaging equipment.

Each well was prepared the DNA-PEI cationic nucleic acid transfection reagent complex according this ratio: dilute 360 ng of Nrf2 phase transition probe plasmid with 10 μL of Opti-MEM serum-free medium (51985034, Gibco, USA), and dilute 0.6 μL of PEI 40000 transfection reagent (40816ES01, Yeasen, China) with 10 μL of Opti-MEM serum-free medium. Hek-293T cells were seeded in 96-well plates and co-incubated with the complex for 1 h. Then, the cells were transferred to normal culture medium and cultured at 5% CO_2_ and 37 °C for 18 h. After stimulation with different concentrations of Cpd.51 for 6 h, photographs were taken and fluorescence intensity was measured.

### Animal

All animal care and experimental procedures complied with the National Institutes of Health Guide for the Care and Use of Laboratory Animals and were approved by the Ethics Committee of Yantai University.

Female and male Sprague-Dawley rats (200-220 g) were used in this study. Animals were free to access food and water and housed with a 12:12 h light/dark cycle, at a temperature of 22-24 °C. The mice were kept five to six per cage and randomly assigned to different experimental groups. All experiments were carried out by investigators who were blinded to experimental group allocations.

Cpd.51 was dissolved in solvent (10% DMSO, 10% Solutol HS-15 and 80% saline). It was injected (i.v.) at dose of 3 mg/kg or 5 mg/kg in a 0.5 mL volume. The Sham group and Vehicle group were treated simultaneously with an equal volume of solvent. In the experiment to explore the treatment time window, we merely administered the drug to the rats at different time points, while all other procedures remained unchanged. In the short-term toxicity study, 10 mg/kg Cpd.51 and Oma were administered via tail vein injection at 0.5 mL daily for 7 consecutive days.

During the long-term toxicity test, rats in each group received daily intragastric administration for 28 consecutive days. Animals in the Sham group were administered a mixture of 99% sesame oil and 1% DMSO at a volume of 0.5 mL per rat. Rats in the remaining groups were intragastrically administered varying doses of the respective therapeutic agents.

### Animal model

All rats were fasted for more than 12 h before the operation began. Transient focal cerebral ischemia was induced by middle cerebral artery occlusion (MCAO) in rat, as previously described 14, 29. In short, after anesthetizing the rats with 2.5% tribromoethanol, the right common carotid artery (CCA), external carotid artery (ECA), and internal carotid artery (ICA) were isolated. A MCAO suture (purchased from RWD Life Science) was inserted into the external carotid artery and advanced along the internal carotid artery from the carotid bifurcation until occlusion was achieved for a duration of 90 min, after which it was removed. The body temperature of the animals was maintained between 36.5 °C and 37.5 °C.

In another set of experiments, adenoviral vector expressing AAV-shNrf2 or AAV-shCon (5 μL of 1.73 × 10^13^ plaque-forming-unit/mL, all produced by Genechem Technology, Shanghai, China) was injected into lateral ventricles (coordinates: Starting from the anterior lobe of the brain, move 2 mm to the right and 1 mm backward and 3.5 mm ventral to skull surface) in the right hemisphere 21 days before tMCAO surgery. The operation of injecting the virus AAV-DHRS3 was the same as before.

The virus sequence we purchased is as follows:

AAV-shNrf2: 5'-GCACTTGTTTGGAGGATTTAA-3'.

AAV-shCon-1: 5'-CGCTGAGTACTTCGAAATGTC-3'.

AAV-shDHRS3: 5'-CCCTCTGCAAATGATCTATTT-3'.

AAV-shCon-2: 5'-GCGTGAATTCTCAGGAACCTC-3'.

### 2, 3, 5-triphenyltetrazolium chloride (TTC) staining

After the behavioral assessments, rats were euthanized and the brain harvested. Brain tissue was cut into five coronal sections (2 mm thick) and incubated in 2% TTC (298-96-4, TCI, Japan) for 20 min at 37 °C. The volume of ischemic brain injury was measured using ImageJ (US National Institutes of Medicine, Bethesda, MD, USA).

### Neurological severity score

To assess the degree of neurological impairment, we conducted modified Neurological Severity Score (mNSS) according to previous experience 14. The investigator was blinded to the grouping situation during the whole experiment.

### Angular test to detect sensorimotor integration function

On postoperative day 3 two wooden boards of identical size were joined at a 30° angle, with a narrow gap retained at the junction. The rat was positioned facing the apex of the angle. When it advanced into the deeper part of the angle, bilateral whisker contact with the boards triggered a characteristic rearing response, followed by a turn to face the opposite direction. Each rat underwent 15 trials, and the number of right turns was recorded. The higher the right-turn ratio, the more severe the injury 30.

### Inverted screen test to assess grip strength

Rats were placed on a horizontal, stainless steel wire grid. The grid was then smoothly rotated 180 degrees to an inverted position and held stationary at a height of 0.8 m. A stopwatch was started immediately upon inversion to measure the latency to fall. The test was terminated if the animal remained on the grid for 60 s, at which point it was gently returned to its home cage, and a score of 60 s was recorded 31.

### Y-maze test for spatial memory assessment

The Y-maze apparatus consisted of three symmetrically arranged enclosed arms (typically labeled Arms A, B, and C), each positioned 120° apart. At the start of each trial, the subject was placed in Arm A and allowed to move freely through the maze for 8 min. Spatial working memory was evaluated by calculating the spontaneous alternation rate, using the following formula:

Accuracy rate of spontaneous alternation (%) = (Total number of arm entries - 2) / Actual number of correct alternations × 100%

### Open field test (OFT) to assess the locomotor activity

Following a 2 h acclimation period, the rats were within an open field cubic box (100 × 100 × 40 cm^3^) and allowed 5 min of unrestricted movement. The travel distance and the time spent in the central area were recorded 32.

### Morris water maze (MWM) test to evaluate memory retention

The spatial learning and memory of rats were assessed using the Morris water maze (MWM) from post-operative days 23 to 26. Over four consecutive days, animals underwent training trials in which they were required to locate a submerged escape platform within 60 s. Each rat was gently placed into the water facing the pool wall. If the rat found the platform and remained on it for 10 s, the escape latency was recorded. If the platform was not found within 60 s, the rat was guided onto it and allowed to stay for 10 s, and the latency was recorded as 60 s. Following the training phase, the platform was removed, and a visual spatial memory test lasting for 60 s was conducted to assess the retention of memory. The number of platform-location crossings and the time spent in the target quadrant were recorded and analyzed 33.

### Transmission electron microscopy (TEM)

After the cells were treated in accordance with the method described in before, they were collected by trypsinization, washed with PBS and centrifuged to collect the cell precipitate, and finally fixed by glutaraldehyde (P1126, Solarbio, China). Afterward, a TEM was utilized to observe the mitochondrial morphology ultrathin sections. The cerebral cortex around the infarction area was also immersed in the fixative after perfusion with PBS and TEM imaging.

### Biochemical index assay

The anesthetized-animals were transcardially perfused with 0.9% saline *in vivo*. The ischemic penumbral tissue was isolated from rats and grinded in PBS. Similarly,* in vitro*, the cells were digested with pancreatic enzymes and placed in PBS. Next, repeatedly freeze and thaw three times in liquid nitrogen and 37 °C water. Sample supernatant was taken for testing according to the MDA or GSH assay kit instructions (S0131M or S0053, Beyotime, China).

Glutamic-pyruvic transaminase (GPT) kits (ml092635, mlbio, China), glutamic oxaloacetic transaminase (GOT) kits (ml092714, mlbio, China), creatinine (CRE) kits (M12C4L, mlbio, China) and blood urea nitrogen (BUN) kits (ml076479, China) were used to detect the serum of rats in order to evaluate liver and kidney functions. Male and female rats were randomly divided into Sham group, Cpd.51 group and Oma group. Doses were administered once daily at 10 mg/kg and body weights were recorded accordingly. Sham groups were given the same volume of solvent. After 7 days, the mice were sacrificed, serum was collected and tested according to the instructions.

During the 28-day long-term toxicity study, rats were euthanized following the completion of continuous intragastric administration. Livers and kidneys from both male and female rats in each group were photographed for macroscopic examination, and serum samples were collected for subsequent kit-based analysis. The liver/kidney index was obtained by dividing the weight of the liver/kidney by the body weight.

### Molecular docking

To elucidate the key pharmacophores of Cpd.51, we performed molecular docking to evaluate its binding mode with the target protein Keap1. Using the crystal structure of the Keap1-BTB domain in complex with CDDO (PDB ID: 4CXT) as a template, Cpd.51 and the target protein were energy-minimized and subjected to docking using Schrodinger. The docking between Cpd.51 and DHRS3 (Uniprot ID: O75911) was the same as described above.

### Nrf2-DHRS3 protein-protein interaction modeling

To rigorously assess the potential protein-protein interaction (PPI) interface between Nrf2 and DHRS3, we performed a new structural prediction using the AlphaFold 3 server (https://alphafoldserver.com). The full-length FASTA sequences for human Nrf2 (Uniprot ID: Q16236) and human DHRS3 (Uniprot ID: O75911) were submitted as input. The resulting model was critically evaluated based on the predicted TM-score (pTM) and, most importantly, the interface-predicted TM-score (ipTM) 27.

### Molecular dynamics simulation

Molecular dynamics simulations can model the trajectories of molecules, yielding information on properties such as binding free energy, system stability, bond types, and flexibility of amino acid residues. In a simulated aqueous physiological environment, we employed the Gromacs software and associated program packages to investigate the stability of the complex formed by Cpd.51 and DHRS3. These results further validated and supported the findings obtained from molecular docking studies.

### Surface plasmon resonance (SPR)

The binding affinity of Cpd.51 or Oma to Keap1 proteins were measured using a Biacore T200. The overall experimental procedures were based on previous reports 9, 28. Human recombinant Keap1 proteins or truncated form of Keap1 (residues 48-180 and residues 48-149) were captured on a CM5 chip by a standard amine coupling procedure. Binding sensorgrams were recorded by injecting various concentrations of Cpd.51 over the immobilized proteins surface. The dissociation constant was obtained by globally fitting the data (to the 1:1 Langmuir binding model using the Biacore Insight evaluation software (Cytiva, Marlborough, MA, USA). Affinity tests for Cpd.51 and DHRS3 were also performed using the aforementioned method.

### LC-MS/MS

Male SD rats were intravenous injection with Cpd.51, blood and brain tissues were collected 15 min, 1 h, 4 h, 6 h, 8 h and 24 h after drug administration. Samples were measured by a Shimadzu chromatography system (Shimadzu, Kyoto, Japan) coupled with an AB Sciex triple quadrupole 4500 mass spectrometer (SCIEX Triple Quad™ 4500 LC-MS/MS, Applied Biosystems Sciex, Ontario, Canada) using Analyst 1.4.2 software. A HPLC column (ACQUITY UPLC BEH C18, 2.1 × 50 mm, 1.7 μm) were used. Protein precipitation method was used for detection: 50 μL plasma sample or brain homogenate sample were added with 200 μL acetonitrile containing internal standard for precipitation, then vortexed for 3 min. The supernatant was injected into system for analysis after centrifugation.

Furthermore, upon completion of the 28-day long-term toxicity study, liver, brain, kidney, and serum samples were randomly collected from the Oma (10 mg/kg) and Cpd.51 (10 mg/kg) dose groups—each comprising 5 males and 5 females—for subsequent tissue distribution analysis. Its operation was the same as that mentioned above.

### Inhibition of CYP450s

The liver microsomal incubation system comprised human, dog, or rat liver microsomal protein (0.75 mg/mL), nicotinamide adenine dinucleotide phosphate (NADPH) generation system, potassium phosphate buffer (0.1 M, pH 7.4), substrate, and inhibitor. The assay was conducted by first preparing a potassium phosphate buffer. Compounds and liver microsomes were then diluted and pre-incubated at 37 °C before initiating the reaction with NADPH. Aliquots were quenched with acetonitrile containing internal standard at specified time points. After sample processing (shaking and centrifugation), the supernatants were analyzed by LC-MS/MS to determine metabolic stability.

### Metabolite identification

*In vitro* identification of metabolites was performed by incubating Cpd.51 (parent compound, final concentration 10 µM) with human liver microsomes (final protein concentration, 1 mg/mL) at 37 °C in a 100 mM potassium phosphate buffer containing 5 mM MgCl_2_. Samples were quenched with methanol and analyzed using a Waters G2-XS Q-Tof UPLC-MS system employing positive-ion and negative-ion electrospray ionization. The LC-MS extract ion chromatograms to identify the major putative metabolites. MS/MS spectra of Cpd.51 and its metabolites were acquired during both positive-ion and negative-ion electrospray. The potential chemical structures of the metabolites were inferred from their MS/MS spectra and retention times. Metabolic pathways of Cpd.51 in human liver microsomes were subsequently proposed.

### Western blotting

The western blotting analysis was conducted as we previously described 35. First, the total protein was extracted using RIRA buffer and then the protein concentrations were determined using the BCA kit (P0012, Beyotime, China). The extraction of mitochondrial proteins was carried out according to the instructions (C3601, Beyotime, China). The samples were separated electrophoretically and then wet-transferred onto 0.2 μm PVDF membranes (88520, Thermo Scientific, USA). After blocking with 5% non-fat dry milk in Tris-buffered saline (TBS) (pH 7.4) containing 0.1% Tween 20 at room temperature for 2 h, the protein membranes were sequentially immersed with the primary antibody at 4 °C overnight and secondary antibody at room temperature for 1 h. Finally, the membranes were quantified using image analysis system. All unprocessed western blotting images and primary antibodies were displayed in Supplementary Document.

### Immunofluorescence and confocal analysis

Assay of ROS generation production: cellular ROS were examined using an ROS Assay Kit (S0033S, Beyotime, China). Cells were incubated with DCFH-DA for 20 min at 37 °C in the dark, and then washed three times with PBS. The fluorescence intensity was measured by Laser Scanning Confocal Microscope (CLSM; ZEISS LSM 800, Germany).

Mitochondrial membrane potential (MMP) and MPTP assessment: MMP changes were measured by a mitochondrial membrane potential assay kit with JC-1 (C2003S, Beyotime, China) according to the manufacturer's instructions. Higher ratios of red to green fluorescence were correlated with higher mitochondrial membrane polarization 36. In addition, MPTP opening was assessed by Calcein-AM assays (Y237214-1mg, Beyotime, China). When the MPTP opened, calcein was lost from the mitochondria and showed a decrease in fluorescence intensity. For detailed instructions, please refer to the manual.

Immunostaining: cells were fixed with 4% paraformaldehyde for 20 min and then incubated with blocking solution (10% normal goat serum, 0.5% Triton X-100 in PBS) for 1 h at room temperature. Then, the samples were incubated overnight with primary antibodies: anti-Nrf2 (1:200, CST), anti-DHRS3 (1:300, Proteintech), anti-NeuN (1:500, Servicebio). When evaluating the extraction efficiency of primary cortical neurons, we performed co-staining with β-tubulin (1:200, Abcam) and Synaptophysin (1:300, ABclonal). After being washed with PBST three times, the samples were incubated with the corresponding secondary antibodies for 2 h in the dark. The nuclei were stained with DAPI (C1006-200mL, Beyotime, China) for 20 min. Finally, images were captured with a fluorescence microscope. Fluorescence integrated density was measured by ImageJ as previously described 37.

Brain tissue was fixed with 4% paraformaldehyde and immersed in 15% sucrose and 30% sucrose at 4 °C overnight. Coronal brain sections (thickness = 20 μm) were incubated with blocking solution for 1.5 h at room temperature followed by incubation with primary antibodies: anti-Nrf2 (1:200, CST), anti-DHRS3 (1:300, Proteintech), anti-NeuN (1:500, Servicebio), anti-GFAP (1:500, Abcam) and anti-Iba-1 (1:500, Abcam). Subsequent immunostaining assays were performed as previously described of cells. Primary antibodies and dilution rate were displayed in Supplementary Document Part II Table S5 Antibody Information.

### Real-time quantitative PCR

To detect the levels of gene expression, the total RNA of SH-SH5Y and brain tissues were extracted by using SPARKeasy Ultra-Pure Total RNA Rapid Extraction Kit (AC0103, Sparkjade, China). Afterward, isolated RNA was reverse-transcribed into cDNA using a cDNA synthesis kit (AG0302-B, Sparkjade, China). Quantitative PCR was performed using synthetic primers and SYBR Green reagent (AH0104-C, Sparkjade, China) according to the standard protocol. The level of GAPDH expression normalized data was used as a reference value to show the expression level change of each gene. The primer pairs in this study were described in Supplementary Document Part II Table S6 Primer Information.

### RNA sequencing and differentially expressed gene analysis

SH-SY5Y cells samples were collected for reference transcriptome sequencing (Oebiotech, Shanghai, China). The fragments per kilobase of exon model per million mapped fragments (FPKM) values of each gene were calculated using Cufflinks, and differential expression analysis was performed using the DESeq R package. Then, we screened the differential protein coding genes according to the multiplicity of differences and the significance of differences test results (Log_2_FoldChange). On significantly differences genes we conducted real-time quantitative PCR verification 38.

### Plasmid transfection

Expression plasmids containing wild type, C151A, G148A and C151A&G148A mutant Keap1 genes, and plasmids targeting at DHRS3 (DHRS3-OE) gene or plasmid of the truncated protein of DHRS3, Nrf2-OE and the negative control (NC-OE) were provided by Genepharma (Shanghai, China).

Hek-293T cells were transiently transfected, using Lipofectamine™ 3000 and P3000™ reagent (L3000075, Invitrogen, USA) according to the manufacturer's instructions, with the wild-type, point-mutant and double-mutations constructs of Keap1, DHRS3 or Nrf2 (each at 0.5 ng/μL), respectively. Similarly, to promote the expression of the DHRS3 gene, a DHRS3 expression plasmids (0.5 ng/μL) was transfected as well as the corresponding negative controls into SH-SY5Y cells for 6 h, and further cultured with fresh medium for 18 h according to the manufacturer's instructions.

To explore the effects of Nrf2 knockdown in the OGD/R model, AAV-shNrf2 virus or AAV-shCon virus at a concentration of 3.5× 10^11^ v.g. /mL to the cells and cultured them for 48 h. The subsequent drug treatment method was the same as before.

### Cellular thermal shift assay (CETSA)

After transfection and drug administration, the Hek-293T cells were collected with PBS. They were heated at 50 °C for 3 min, then equilibrated at 25 °C for 3 min. The sample was freeze-thawed three times with liquid nitrogen, and the supernatant was separated by centrifugation (13,000 × g, 20 min, 4 °C). The collected supernatant was mixed with 5× SDS-PAGE loading buffer and heated at 100 °C for 10 min. Finally, the samples were separated by 10% SDS-PAGE gel.

SH-SY5Y cells were incubated with DMSO or Cpd.51 (100 nM, dissolved in DMSO) under standard culture conditions for 12 h. After washing twice with PBS, the cells were collected and mixed in PBS containing a mixture of protease and phosphatase inhibitor cocktail. They were evenly divided into 7 groups, heated in a preset temperature (37-55 °C) mode for 3 min and balanced at 25 °C for 3 min. The subsequent operations were the same as before.

### Drug affinity responsive target stability (DARTS)

DARTS was performed according to a published protocol. SH-SY5Y cells were treated with Cpd.51 or DMSO for 12 h, and lysed with lysis buffer. The supernatant was collected by centrifugation at 12,000 × g for 10 min at 4 °C and protein concentration was determined by BCA assay. Pronase (10165921001, Merck, Germany) was added proportionally according to the sample protein content and incubated for 30 min at room temperature to full reaction 39. Next, 5× SDS-PAGE loading buffer was added to the samples and heated to 100 °C for 10 min to prepare for western blotting analysis.

### Co-IP and GST pull down

Co-IP experiments were performed as described 34. After SH-SY5Y cells were treated, lysates were collected and incubated with antibodies with rotation for 12 h at 4 °C. Then, they were incubated with protein A/G magnetic beads (88804, Thermo Scientific, USA) as specification. Subsequently, the beads were collected and immune complexes were isolated. Finally, protein complexes were incubated for 5 min at 95 °C after dissolving in the electrophoresis sample buffer, and subjected to western blotting.

We demonstrated a direct physical interaction between Nrf2 and DHRS3 through a pull-down assay. GST-Nrf2 (YB710012, Ybio, China) or GST (Ag0040, Proteintech, China, as control) proteins were mixed with GST Resin (EA-IP-K008, Elabscience Biotechnology, China) for 2 h incubation on the shaker at 4 °C. Subsequently, both Cpd.51 (100 nM) and DHRS3 (YB765100, Ybio, China) were added to the mixture and rotated overnight at 4 °C 40. Then, following the instructions, the sample buffer was added and heated to 100 °C for 10 min. After completing all steps, the mixture was centrifuged, and the supernatant was collected for western blotting analysis.

To determine the Nrf2-binding region within DHRS3, several truncated forms of DHRS3 (1-215 aa, 1-190 aa, 1-100 aa) were constructed and GST pull-down assays were performed. All procedures were performed following the same method as previously described.

### Chromatin immunoprecipitation assays (ChIP)

The promoter region sequence of the DHRS3 gene was identified through the NCBI database and the UCSC Genome Browser database. The potential Nrf2 binding sites within the DHRS3 promoter region were predicted using the JASPAR database. Subsequently, based on these findings, we conducted ChIP experiments for validation.

SH-SY5Y cells were treated with 100 nM Cpd.51 or DMSO for 12 h, and chromatin immunoprecipitation assays were performed using a ChIP assay kit (53040, Active Motif, USA), according to manufacturer instructions. Cells were first cross-linked by adding formaldehyde and the cross-linking was stopped by the addition of glycine. Samples interrupted with sonication followed by ChIP with protein G agarose beads. Finally, DNA fragments were purified and were amplified by real-time PCR using SYBR green, with the *DHRS3* primers (F: 5′-TGGTCAGAGTGGGAAGAGGT-3′, R: 5′-ACTTTGAACTGGGGCTGCTT-3′).

### Statistics

All statistical analysis were performed using GraphPad Prism 8 Software (La Jolla, CA, USA). Data were expressed as the means ± SD. Statistical differences among groups were analyzed by using One-way ANOVA or Two-way ANOVA followed by Tukey's post-hoc test, and Student's t test. Behavior data were analyzed using non-parametric Mann Whitney test. *P* < 0.05 was considered statistically significant.

## Results

### Cpd.51, an Omaveloxolone derivative, exerts its anti-AIS effect by specifically increasing Nrf2 expression in neurons

**Figure 1:**
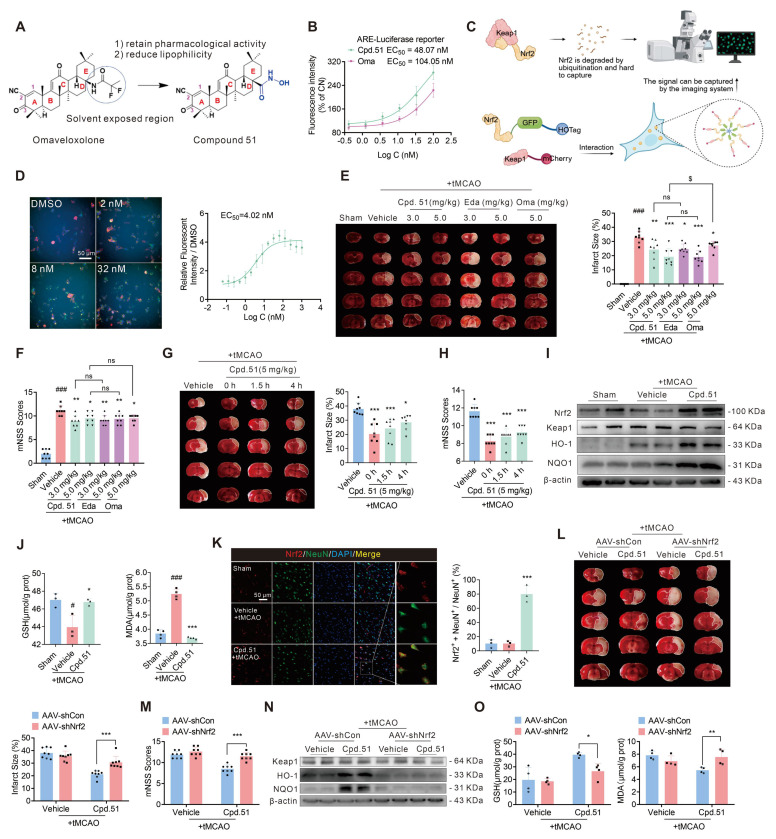
** The anti-AIS effect of Cpd.51 was mediated by the specific activation of neuronal Nrf2.** (A) Structures of Omaveloxolone (Oma) and Compound 51 (Cpd.51). (B) The experimental results of the luciferase reporter gene following indicated concentrations of Cpd.51 and Oma in Hek-293T cell. *n* = 4. (C) Schematic diagram of the design principle for a fluorescent sensor system. (D) The EC_50_ values of Cpd.51 for activation of Nrf2 was 4.02 nM in Hek-293T cell by fluorescent sensor system test.* n* = 6. (E, F) Cpd.51 administration reduced cerebral infarct volume and promoted neurological functions in rats. *n* = 8. (G, H) When intravenously injected at 0 h, 1.5 h and 4 h after ischemia, Cpd.51 (5 mg/kg) consistently alleviated cerebral infarct volume and improved neurological function at 24 h after tMCAO as evaluated by TTC staining and mNSS test. *n* = 8. (I) Western blotting analysis for the protein expression of Nrf2, Keap1, HO-1, and NQO1 in the cortex surrounding the infarct area of the experimental rats at 24 h post tMCAO.* n* = 4. (J) Analysis of GSH and MDA content in peri-infarct cortex.* n* = 3 or 4. (K) Representative immunofluorescence images and quantification of neuron stained with Nrf2, and quantitative analysis of Nrf2^+^ NeuN^+^ cells in the peri-infarct region of rats at 24 h after ischemia. *n* = 3. (L) Representative of TTC-stained brain slices and infarct volume statistics. *n* = 8. (M) Neurobehavioral scores measured by mNSS. *n* = 8. (N) Western blotting analysis for the protein expression of Keap1, HO-1, NQO1 in the cortex surrounding the infarct area of the experimental rats. *n* = 4. (O) Analysis of GSH and MDA content in the cortex surrounding the infarct area of rats. *n* = 4. Results are expressed as mean ± SD; E-K, ^#^*P <* 0.05,^ ###^*P <* 0.001 vs. Sham group. ^*^P < 0.05, ^**^P < 0.01, ^***^P < 0.001 vs. tMCAO group.^*$*^*P <* 0.05 vs. Cpd.51 5 mg/kg group. L-O, ^*^P < 0.05, ^**^P < 0.01, ^***^P < 0.001 vs. Cpd.51 plus tMCAO plus AAV-shCon group. F and H, Behavior data were analyzed using non-parametric Mann Whitney test. E, G, J and K, Statistical differences among groups were analyzed by using one-way ANOVA followed by Tukey's post-hoc test. L-O, Statistical differences among groups were analyzed by using two-way ANOVA followed by Tukey's post-hoc test.

Omaveloxolone (Oma) has demonstrated clinical efficacy but is associated with hepatotoxicity, with ~37% of patients developing liver injury during clinical trials. Consequently, routine liver function monitoring is required, complicating clinical management and narrowing its therapeutic window. Physicochemical property prediction using ACD/Labs software indicated that Oma was highly lipophilic (cLogP = 6.14). In addition, clinical pharmacokinetic studies revealed an extremely large apparent volume of distribution (Vd 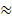
105 L/kg) and a long terminal half-life (T_1/2_ 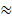
57 h) 9. Together, these features suggested that the hepatotoxicity of Oma might be attributable to hepatic accumulation driven by its high lipophilicity. Based on this rationale, we designed a molecular optimization strategy aimed at reducing lipophilicity while retaining pharmacological activity. We modified C17 amide by sequentially replacing the carbonyl and amino groups and subsequently introducing a hydroxyl functionality, leading to the design of a hydroxylamine derivative (Cpd.51, Purity: 99.1%) (Figure 1A). Prediction of physicochemical properties using ACD/Labs further confirmed that Cpd.51 displayed reduced lipophilicity compared with Oma (Oma: cLogP = 6.19; Cpd.51: cLogP = 4.90).

To determine whether Cpd.51 possessed Nrf2-activating activity, an ARE-luciferase reporter gene assay and a specific fluorescent sensor system were employed. As shown in Figure 1B, Cpd.51 dose-dependently increased ARE fluorescence intensity, exhibiting an EC_50_ of 48.07 nM, which was lower than that of Oma (Purity: 99.29%, 104.05 nM). Nrf2 is constitutively regulated through Keap1-mediated ubiquitination and proteasomal degradation, maintaining low baseline levels under physiological conditions. To further examine the direct effect of Cpd.51 on Nrf2 activation, we utilized a specific fluorescent sensor system (Figure 1C). Immunofluorescence analysis revealed that Cpd.51 treatment inhibited the degradation of Nrf2, manifested as promoting a transition of Nrf2 (green) localization from diffuse cytosolic patterns to discrete, co-localized puncta (approximately 2 µm in diameter) (Figure 1D). These results indicated that our structural modification did not reduce the Nrf2-activating activity.

Building on the potent Nrf2 activation by Cpd.51, we evaluated its therapeutic potential against AIS-induced injury and analyzed possible target cells. Then, the therapeutic potential of Cpd.51 on AIS was measured in rat transient Middle Cerebral Artery Occlusion (tMCAO) model. As shown in Figure 1E, an extensive lesion was found in both striatum and cortex of tMCAO rats. Intravenous injection of 3 mg/kg and 5 mg/kg of Cpd.51 at the same time as ischemia significantly reduced the area of infarction. In terms of improving neurobehavioral outcomes, Cpd.51 significantly inhibited the increase in mNSS scores caused by tMCAO (Figure 1F). Crucially, delayed administration of Cpd.51 at 4 h post-ischemia still significantly diminished infarction volume and improved mNSS scores (Figure 1G, 1H).

As a key transcriptional regulator of redox homeostasis, activated Nrf2 translocates to the nucleus and dimerizes with sMaf proteins, binding to ARE to drive expression of cytoprotective genes involved in detoxification and antioxidant defense 41. Based on this canonical mechanism, we assessed whether Cpd.51 stabilized Nrf2 and enhanced downstream protein expression in the tMCAO model. Quantitative immunoblotting demonstrated that Cpd.51 significantly upregulated Nrf2, Heme oxygenase 1 (HO-1), and NAD(P)H quinone oxidoreductase (NQO1) protein levels in peri-infarct cortex (Figure 1I, S1A). Consistent with Nrf2-mediated antioxidant function, tMCAO-induced oxidative stress was evidenced by depleted glutathione (GSH) and elevated malondialdehyde (MDA) in cortical tissues. Cpd.51 treatment markedly reversed these perturbations, restoring GSH concentrations and suppressing MDA accumulation (Figure 1J).

Nrf2 activity is spatiotemporally regulated across pathological progression and exhibits cell-type dependency 42. In the ischemic penumbra, Nrf2 expression changed in both glial cells and neuronal populations, with astrocytes showing significantly higher levels than neurons 43, 44. To identify the primary cellular target of Cpd.51-induced Nrf2 activation, we performed co-staining for Nrf2 with neuronal (NeuN) and glial (GFAP/Iba-1) markers. Nrf2 was selectively enriched in neurons following Cpd.51 treatment (Figure 1K, S1B and S1C). Collectively, these results demonstrated that Cpd.51 attenuated AIS damage by promoting neuron-specific Nrf2 activation.

To further determine whether Cpd.51's neuroprotective effects were mediated through the Nrf2 pathway, we constructed an Nrf2-knockdown rat model by injection of AAV-mediated shRNA delivery in right lateral ventricle (Figure S1D). Western blotting analysis confirmed significant downregulation of Nrf2 expression in the cerebral cortex of AAV-shNrf2-treated rats (Figure S1E). AAV-mediated Nrf2 knockdown substantially abrogated the protective effects of Cpd.51, evidenced by attenuated reduction in infarct volume (Figure 1L), deteriorated neurobehavioral scores (Figure 1M) and reversed upregulation of Nrf2-dependent antioxidant markers (HO-1, NQO1) in the peri-infarct cortex (Figure 1N, S1F). Concomitantly, Cpd.51-induced modulation of oxidative stress indicators (elevated GSH, reduced MDA) was nullified by Nrf2 silencing (Figure 1O).

### Cpd.51 promoted long-term recovery of neurobehavioral function after ischemia

**Figure 2:**
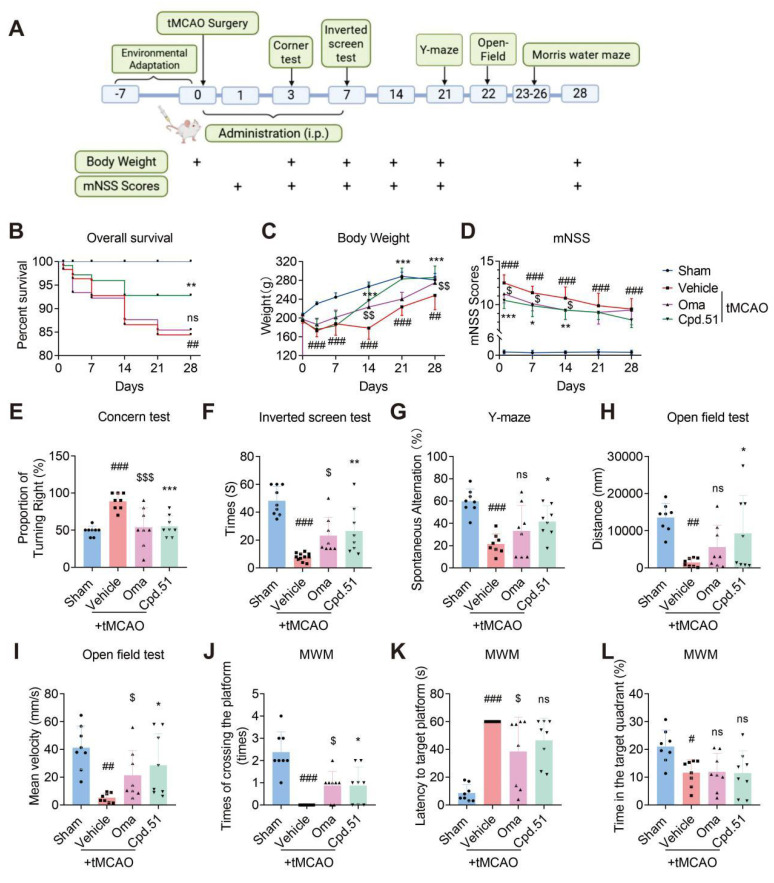
** Cpd.51 promoted Long-term recovery of neurobehavioral function after ischemia.** (A) Structures of the experiment process. (B) Comparison of survival rates among different groups of rats. (C-L) Cpd.51 and Oma promoted long-term functional recovery as evaluated by body weight, mNSS, corner test, inverted screen test, Y maze test, open field test, and Morris water maze test. *n* = 8. Results are expressed as mean ± SD; ^#^*P <* 0.05,^ ##^*P <* 0.01, ^###^*P <* 0.001 vs. Sham group. ^*^P < 0.05, ^**^P < 0.01, ^***^P < 0.001 vs. tMCAO group.^ $^*P <* 0.05, ^$$$^*P <* 0.001 vs. tMCAO group. Statistical differences among groups were analyzed by using non-parametric Mann Whitney test.

To determine and compare the role of Cpd.51 and Oma in long-term outcomes of rats after stroke, we administered via tail vein injection for 7 d, at a dose of 5 mg/kg. We assessed the effects of the two compounds by comparing post-tMCAO survival rate, body weight and multiple behavioral test outcomes. The specific procedures are illustrated in Figure 2A. Results indicate that Cpd.51 and Oma significantly improved body weight and mNSS scores in tMCAO rats, but only Cpd.51 reduced mortality (Figure 2B-D). Furthermore, rats administered Cpd.51 or Oma exhibited improvements in neurological deficits, motor abilities, and spatial working and reference memory during the post-stroke period. These enhancements were evidenced by the results of the corner test, inverted screen test, Y maze test, and open field test, in comparison to the model group (Figure 2E-I). Morris water maze test (MWM), the established gold standard for evaluating spatial learning and memory, revealed that Cpd.51 has only a modest capacity to improve these cognitive functions. In summary, while Cpd.51 promoted long-term neurological recovery in rats following cerebral ischemia, it did not demonstrate significant advantages over Oma. Furthermore, the improvement effect of Cpd.51 on neurological function and motor function seemed to be relatively obvious (Figure 2J-L). Collectively, the comparative benefit of Cpd.51 over Oma appears to be phase-dependent, with greater efficacy observed during the acute phase relative to the later functional recovery stage following ischemic stroke.

### Cpd.51 alleviated oxygen glucose deprivation/reperfusion (OGD/R) induced neuronal damage by activating Nrf2

**Figure 3:**
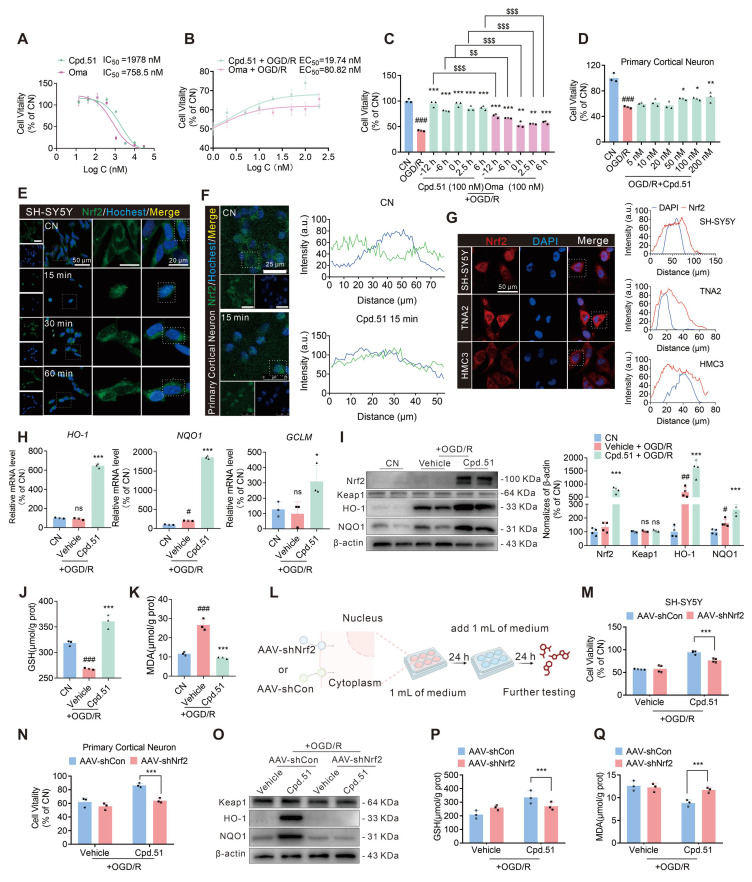
** Cpd.51 alleviated OGD/R induced neuronal damage by activating Nrf2.** (A) IC_50_ assay of Cpd.51 and Oma in SH-SY5Y cells after 24 h incubation. *n* = 3. (B) The EC_50_ values of Cpd.51 and Oma against OGD/R damage in SH-SY5Y cells. *n* = 3. (C) When administered at different time points, Cpd.51 (100 nM) and Oma (100 nM) consistently alleviated OGD/R damage as evaluated by CCK-8, and the effect of Cpd.51 was better than Oma. *n* = 3. (D) Cpd.51 ameliorated damage to primary cortical neurons cells by OGD/R as measured by CCK-8. *n* = 3. (E, F) SH-SY5H cells or primary cortical neurons treated with Cpd.51 (100 nM) for 15 min promote Nrf2 nuclear translocation, as demonstrated by immunofluorescence staining and confocal microscopy imaging. (G) Neurons, astrocytes, microglia cells treated with Cpd.51 (20 nM) for 15 min promote Nrf2 nuclear translocation, as demonstrated by immunofluorescence staining and confocal microscopy imaging. (H) The qRT-PCR analysis for the genes expression of *HO-1*, *NQO1* and *GCLM* in SH-SY5Y cells at 24 h post OGD/R.* n* = 3. (I) Western blotting analysis for the protein expression of Nrf2, Keap1, HO-1, and NQO1 in SH-SY5Y cells at 24 h post OGD/R.* n* = 4 (J-K) Analysis of GSH and MDA content in SH-SY5Y cells at 24 h post OGD/R.* n* = 3. (L) Schematic diagram of Nrf2-knockdown process *in vitro* models. (M, N) CCK-8 assays showed that Nrf2-knockdown reversed the protective effects of Cpd.51 in SH-SY5Y and primary cortical neurons cells with OGD/R. *n* = 4 or 3. (O) Western blotting analysis for the protein expression of Keap1, HO-1, NQO1 in Nrf2-knockdown SH-SY5Y cells.* n* = 3. (P, Q) Analysis of GSH and MDA content in Nrf2-knockdown SH-SY5Y cells. *n* = 3. Results are expressed as mean ± SD; C-K, *^#^P <* 0.05, ^##^*P <* 0.01, ^###^*P <* 0.001 vs. CN group. ^*^P < 0.05, ^**^P < 0.01, ^***^P < 0.001 vs. OGD/R group.^*$$*^*P <* 0.01, *^$$$^P <* 0.001 vs. Cpd.51 plus OGD/R group. Statistical differences among groups were analyzed by using One-way ANOVA followed by Tukey's post-hoc test. M-Q,^ ***^P < 0.001 vs. Cpd.51 plus OGD/R plus AAV-shCon group. Statistical differences among groups were analyzed by using Two-way ANOVA followed by Tukey's post-hoc test.

Given the pronounced neuronal selectivity of Cpd.51, we further validated its neuroprotective effects *in vitro*. Specifically, CCK-8 assays were performed to assess both the compound's cytotoxicity and its efficacy against OGD/R-induced injury in neuron, astrocytes, and microglia (Figure S2A-F). Consistent with *in vivo* observations, Cpd.51 demonstrated superior neuroprotective effects and safety compared to Oma in SH-SY5Y cells (Figure 3A-C). More convincingly, Cpd.51 also demonstrated excellent efficacy in OGD/R-induced primary neuronal damage (Figure 3D, S2G).

To validate that the observed neuroprotection was specifically mediated by Nrf2 activation via Cpd.51, we first performed immunofluorescence (IF). We found that Nrf2 nuclear translocation within 15 min post Cpd.51 treatment in SH-SY5Y cells and primary cortical neurons (Figure 3E, 3F and S2H). Of particular significance was our finding that Cpd.51 induced more pronounced nuclear translocation of Nrf2 in neuronal cells even at the lower dose of 20 nM. This efficacy at lower concentrations may underlie the superior protective effects on neuron observed for Cpd.51 (Figure 3G). Subsequent qRT-PCR and immunoblotting confirmed upregulation of antioxidant genes/proteins downstream of Nrf2 (Figure 3H, 3I), with concurrent restoration of GSH levels and suppression of MDA accumulation (Figure 3J, 3K). Although Nrf2 downstream proteins were upregulated in response to Cpd.51 across all three cell types, the magnitudes were different. Notably, neurons exhibited heightened sensitivity to Cpd.51, reflected by a more pronounced increase in the levels of these proteins (Figure S2I, S2J). Interestingly, following OGD/R injury, glial cells demonstrated a downregulation of Nrf2 downstream protein expression, in contrast to neurons (Figure S2I, S2J). The mechanism underlying this differential response, however, remains to be elucidated.

To further validate the neuron-specific role of this pathway, we employed an *in vitro* model using SH-SY5Y cells and primary cortical neurons. Transduction with AAV-shNrf2 for 48 h significantly reduced Nrf2 expression in both cell types (Figure 3L, S2K). Nrf2 knockdown markedly attenuated the protective effects of Cpd.51 against OGD/R-induced cytotoxicity, as evidenced by reduced cell viability in SH-SY5Y cells and primary cortical neurons (Figure 3M, 3N). Consistent with *in vivo* observations, Nrf2 silencing reversed Cpd.51-mediated upregulation of HO-1, NQO1 and GCLM at mRNA and protein levels (Figure 3O, S2L-Q), suppressed GSH elevation (Figure 3P), and abrogated MDA reduction (Figure 3Q) in OGD/R-injured SH-SY5Y cells. These results demonstrated that Cpd.51 activated the Nrf2 pathway, and provided neuroprotective effects against OGD/R-induced damage *in vitro*. Furthermore, Cpd.51 exhibited enhanced safety and superior efficacy relative to Oma.

### Cpd.51 repaired mitochondrial dysfunction after AIS damage by Nrf2-activation

Mitochondria, as the “cellular power centers”, are essential in cellular energy homeostasis and post-ischemic neurological recovery 45. Given the intimate relationship between mitochondrial damage and the pathological process of AIS, we investigated Cpd.51's effects on mitochondrial function following Nrf2 activation.

**Figure 4:**
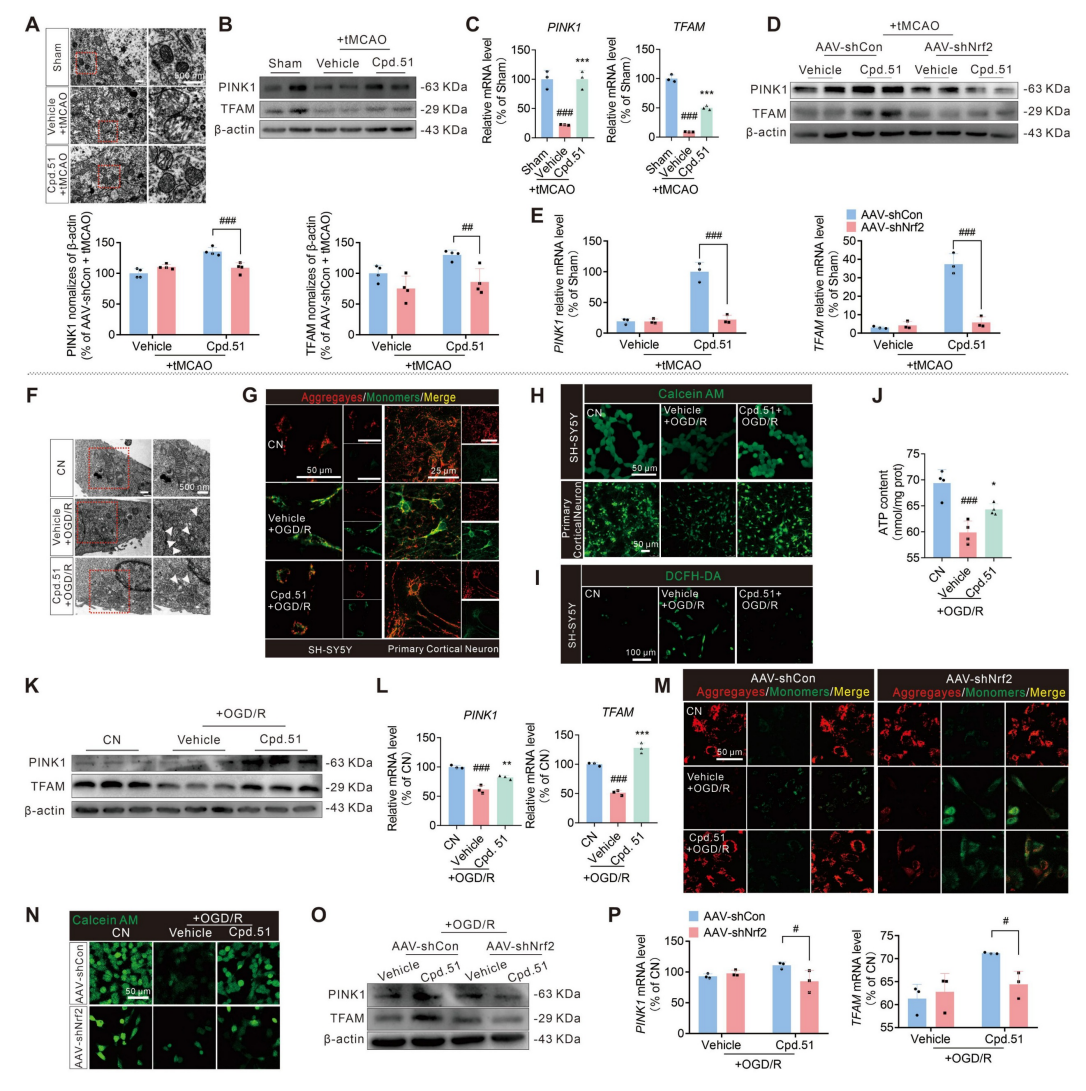
** Cpd.51 repaired mitochondrial dysfunction after AIS damage by Nrf2-actived.** (A) The ultrastructural morphology of mitochondria in the cortex surrounding the infarct area of rats was examined by transmission electron microscopy. (B, D) Western blotting analysis for the protein expression of PINK1 and TFAM in the cortex surrounding the infarct area of the rats. *n* = 4. (C, E) The qRT-PCR analysis for the gene's expression of *PINK1* and *TFAM* in the cortex surrounding the infarct area. *n* = 3. (F) The ultrastructural morphology of mitochondria in SH-SY5Y cells was examined by transmission electron microscopy. (G, H) Cpd.51 protected SH-SY5Y and primary cortical neuronal cells in OGD/R model by improving mitochondrial function, as demonstrated by JC-1 assay for mitochondrial membrane potential and Calcein AM assay for mitochondrial permeability transition pores (MPTP). *n* = 3. (I) DCFH-DA staining revealed that Cpd.51 reduced intracellular ROS production in SH-SY5Y cells following OGD/R. *n* = 3. (J) Cpd.51 restored ATP production in SH-SY5Y cells following OGD/R injury. *n* = 4. (K, O) Western blotting analysis for the protein expression of PINK1 and TFAM in SH-SY5Y cells. *n* = 3. (L, P) The qRT-PCR analysis for the gene's expression of *PINK1* and *TFAM* in SH-SY5Y cells. *n* = 3. (M, N) Nrf2-knockdown reversed the protective effects of Cpd.51 about mitochondrial function in SH-SY5Y cells with OGD/R, as demonstrated by JC-1 assay for mitochondrial membrane potential and Calcein AM assay for MPTP. *n* = 3. Results are expressed as mean ± SD; C,^ ###^*P <* 0.001 vs. Sham group,^ ***^P < 0.001 vs. tMCAO group. J and L, ^###^*P <* 0.001 vs. CN group,^ *^P < 0.05, ^**^P < 0.01, ^***^P < 0.001 vs. OGD/R group. Statistical differences among groups were analyzed by using One-way ANOVA followed by Tukey's post-hoc test. D and E, ^##^*P <* 0.01 ^###^*P <* 0.001 vs. Cpd.51 plus tMCAO plus AAV-shCon group. P, ^**^P < 0.01, ^***^P < 0.001 vs. Cpd.51 plus OGD/R plus AAV-shCon group. Statistical differences among groups were analyzed by using two-way ANOVA followed by Tukey's post-hoc test.

Transmission electron microscopy indicated loss of mitochondrial cristae in peri-infarct cortical regions following tMCAO, which was ameliorated by Cpd.51 (Figure 4A). This finding suggested that Cpd.51 mitigated mitochondrial damage in cortical tissue caused by tMCAO to a certain extent. Studies have shown that PTEN-induced putative kinase 1 (PINK1) mediates mitochondrial autophagy 46, while mitochondrial transcription factor A (TFAM) regulates the maintenance, expression, and transmission of mitochondrial DNA (mtDNA) 47. For severely damaged and irreparable mitochondria, the PINK1 pathway is activated to guide their clearance through the autophagy pathway. To assess Cpd.51's effects on ischemia-reperfusion-impaired mitochondrial function, we quantified PINK1 and TFAM in peri-infarct cortex. Ischemia-reperfusion significantly downregulated PINK1 and TFAM mRNA/protein expression, while Cpd.51 treatment restored these levels (Figure 4B, 4C, S3A), and those results were reversed after Nrf2 knockdown (Figure 4D, 4E).

In the OGD/R model, we performed a more detailed evaluation of mitochondrial function in SH-SY5Y cells and primary cortical neuron cells. Treatment with Cpd.51 significantly ameliorated mitochondrial morphological damage (Figure 4F), enhanced mitochondrial membrane potential (Figure 4G, S3B and S3C), reduced mitochondrial permeability (Figure 4H, S3D and S3E), decreased reactive oxygen species production (Figure 4I, S3F) and increased ATP production (Figure 4J). Furthermore, the expression patterns of both mRNA and protein for PINK1 and TFAM supported the role of Cpd.51 in maintaining mitochondrial function during OGD/R injury (Figure 4K, 4L, S3G). Consistent with the *in vivo* results, the mitochondrial protective effect of Cpd.51 was abrogated in Nrf2-knockdown SH-SY5Y cell model (Figure 4M-P, S3H-K). The results above indicated that Cpd.51 mitigated ischemia-reperfusion-induced mitochondrial damage in neurons through an Nrf2-dependent mechanism.

### Cpd.51 activates Nrf2 partially by binding to glycine 148 and cysteine 151 of Keap1

**Figure 5:**
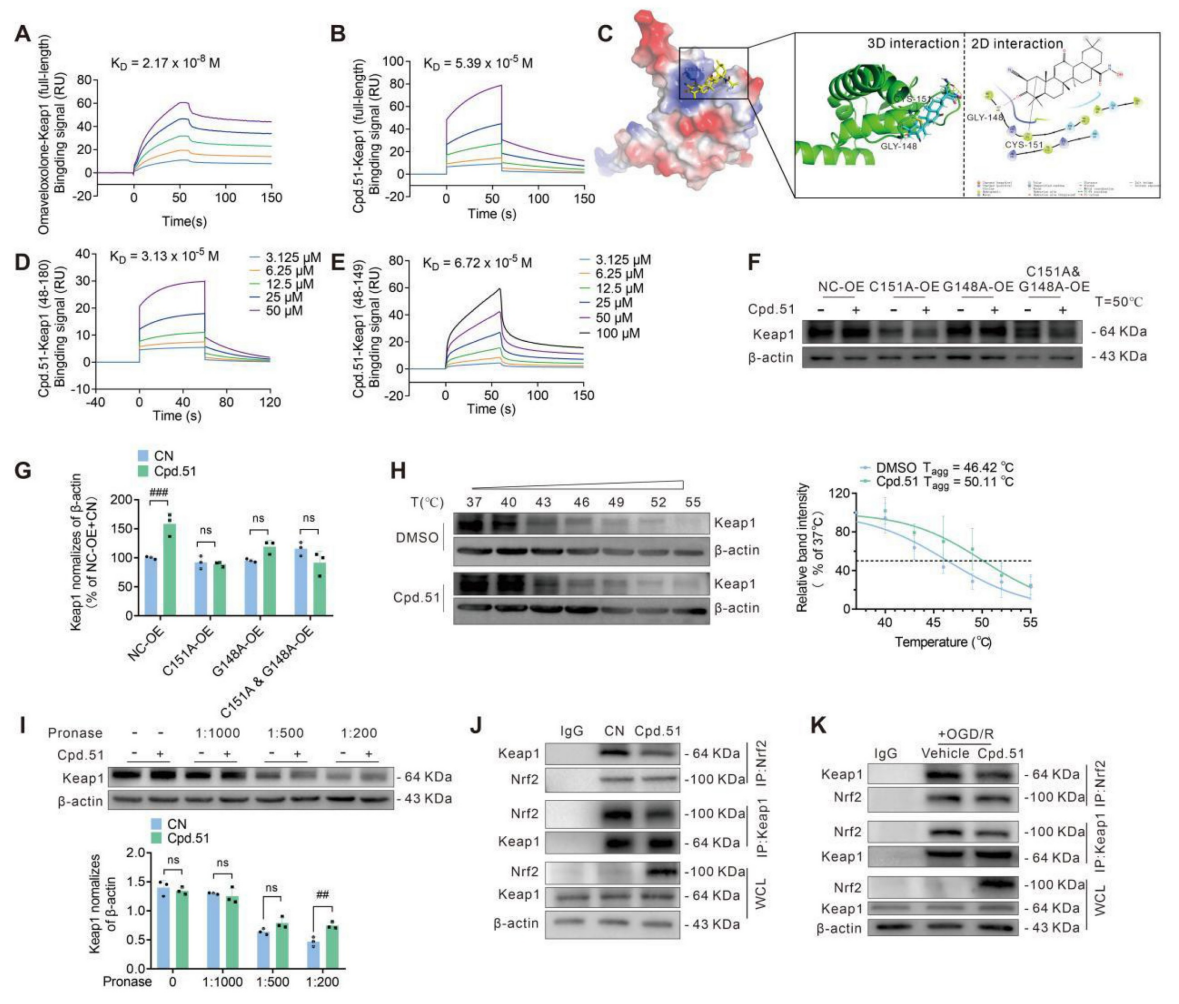
** Cpd.51 exerted Nrf2-activating effects by binding to the Keap1 Gly148 and Cys151 positions.** (A) Surface Plasmon Resonance (SPR) was employed to determinate of the binding affinity between Oma and the Keap1 protein. (B, D, E) SPR was employed to determinate of the binding affinity between Cpd.51 and the Keap1 protein. (C) Three-dimensional and two-dimensional structural models of Cpd.51 and Keap1 proteins, as well as the specific binding interfaces mediating their direct interaction, were generated using molecular docking approaches. (F, G) CETSA analysis of intracellular binding between Cpd.51 and Keap1 was performed at 50 °C in Hek-293T cells transfected with Keap1 plasmid.* n* = 3. (H) CETSA analysis of intracellular binding between Cpd.51 (100 nM) and Keap1 at different temperature in SH-SY5Y cells.* n* = 3. (I) SH-SY5Y cells were lysed and incubated with Cpd.51 (100 nM) or DMSO for 30 min at 25 °C. DARTS assays were performed with pronase (20 ng/μg protein), and lysates were analyzed by immunoblotting.* n* = 3. (J, K) Immunoprecipitation assay of the interaction in whole cell lysate between Nrf2 and Keap1 in physiological or OGD/R conditions, and SH-SY5Y cells treated with Cpd.51 (100 nM) or Vehicle (DMSO). *n* = 3. Results are expressed as mean ± SD; ^##^*P <* 0.01, ^###^*P <* 0.001. Statistical differences among groups were analyzed by using one-way ANOVA followed by Tukey's post-hoc test.

Keap1 serves as a key negative regulator of Nrf2 via direct binding, making it a highly attractive target for pharmacological intervention aimed at Nrf2 activation 48. To elucidate how Cpd.51 activates Nrf2, our initial focus was placed on characterizing its binding affinity for Keap1. Firstly, we quantified the binding kinetics of Cpd.51 and Oma to Keap1 using surface plasmon resonance (SPR). Intriguingly, SPR measurements indicated that Cpd.51 exhibits moderate affinity for Keap1 (K_D_ ≈ 5.39 × 10^⁻5^ M, Figure 5B), and it is significantly lower than Oma (K_D_ ≈ 2.17 × 10^⁻8^ M, Figure 5A).

Secondly, to elucidate the key pharmacophores of Cpd.51, we performed molecular docking to evaluate its binding mode with the target protein Keap1 (Figure 5C). Using the crystal structure of the Keap1-BTB domain in complex with CDDO (PDB ID: 4CXT) as a template, Cpd.51 and the target protein underwent energy optimization and were subjected to docking operations. The docking results showed that the C1-C2 α, β-unsaturated double bond of Cpd.51 formed an irreversible covalent bond with Cys151, thereby anchoring the molecule in the binding pocket. The C3 carbonyl group served as a hydrogen bond acceptor for Gly148, stabilizing the binding conformation. Moreover, the pentacyclic scaffold (rings A-E) of Cpd.51 engaged in extensive hydrophobic interactions with the protein, further enhancing binding stability (Figure 5C). We also designed two truncated version of the Keap1 protein to allow for a precise assessment of binding to these specific residues (Cys151 and Gly148) by SPR analysis. Cpd.51 exhibited high-affinity binding to full-length Keap1 and the BTB domain (48-180) of Keap1 (K_D_ ≈ 3.13 × 10⁻⁵ M), whereas the affinity for the BTB domain fragment (48-149) was reduced (K_D_ ≈ 6.72×10⁻⁵ M) (Figure 5B, 5D, 5E). However, this difference, was relatively small. This led us to conclude that although the key binding site for Cpd.51 with Keap1 was Cys151, the role played by the Gly148 position could not be overlooked. Besides, the response curve decreased after 100 s of experiment, suggesting that the binding was reversible. This is entirely different from the binding curve of Oma with Keap1, suggesting that the interaction between Cpd.51 and the Cys151 site of Keap1 may be non-covalent.

To functionally validate the contributions of Cys151 and Gly148 to Cpd.51 binding and activity, we also generated Keap1 point / double mutants (C151A, G148A, and C151A & G148A) and assessed compound responsiveness via CETSA in Hek-293T cells. All mutations significantly attenuated Cpd.51-induced thermal stabilization of Keap1 compared to wild-type controls (Figure 5F, 5G, S4A-C). Notably, the C151A mutation and C151A/G148A double mutant nearly abolished compound efficacy, while the G148A mutant also showed substantial reduction. These data demonstrated that Cpd.51 activated Nrf2 through modification of Keap1 at Cys151 and Gly148.

Next, to further confirm the Cpd.51-Keap1 interaction in a neuronal context, CETSA and DARTS assays were employed to demonstrate the binding between Cpd.51 and Keap1. The results validated enhanced thermal and enzymatic stability of Keap1 in neural cells after Cpd.51 treatment versus DMSO controls (Figure 5H, 5I). To further analyze whether the interaction between Cpd.51 and Keap1 affects the amount of Keap1-bound Nrf2, we assessed the Keap1-Nrf2 binding under both physiological and OGD/R conditions by co-immunoprecipitation (Co-IP). Cpd.51 significantly promoted the expression of Nrf2 in cells and reduced the amount of Nrf2 bound to Keap1 (Figure 5J, 5K and S4D-K).

Collectively, the data indicated that Cpd.51 activated Nrf2 by engaging Keap1 at C151 and G148, and this activity was preserved under OGD/R. Notably, although Cpd.51 showed reduced binding affinity for Keap1 relative to Oma, it retained strong Nrf2 activation efficacy. This observed divergence between binding affinity and functional output implied the involvement of a non-classical activation pathway.

### DHRS3 participated in the repair of mitochondrial function by Cpd.51

**Figure 6:**
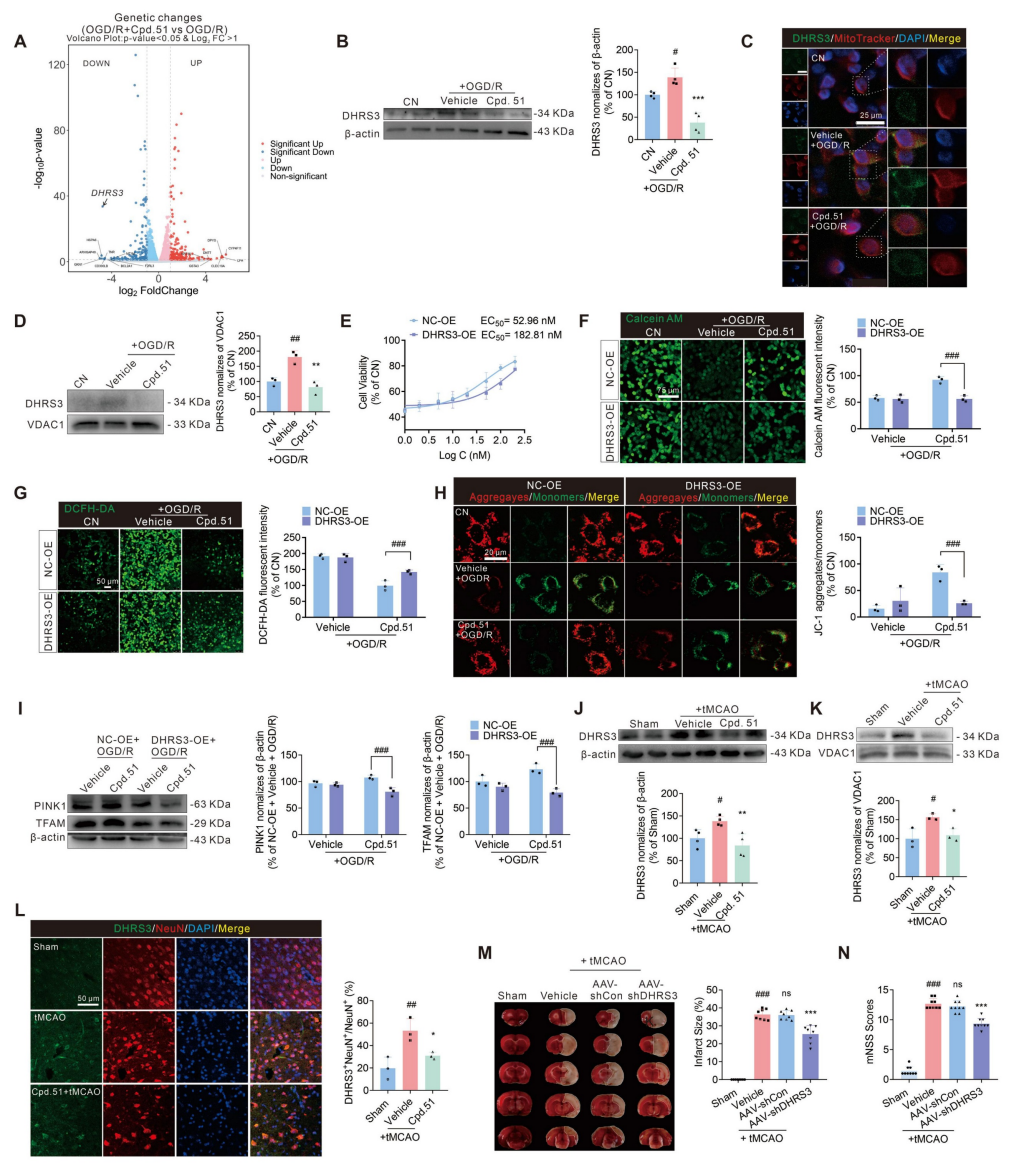
** DHRS3 participated in the repair of mitochondrial function by Cpd.51.** Volcano plots displayed the distribution of differentially expressed genes. OGD/R plus Cpd.51 compared with OGD/R.* n* = 3. (B) Western blotting analysis for the protein expression of DHRS3 in SH-SY5Y cells. *n* = 4. (C) Immunofluorescence assay of SH-SY5Y cells were co-stained for Mitotracker and DHRS3. (D) Western blotting analysis of DHRS3 protein expression in mitochondria of SH-SY5Y cells.* n* = 3. (E) DHRS3-overexpression reversing the protective effects of Cpd.51 in SH-SY5H cells, as evidenced by a decrease in EC_50_. *n* = 3. (F-I) DHRS3-overexpression reverses the protective effect of Cpd.51 on mitochondria in SH-SY5Y cells following OGD/R injury, as evaluated by MPTP detection, ROS detection, JC-1 staining, PINK1 protein levels and TFAM protein levels. *n* = 3. (J) Western blotting analysis for the protein expression of DHRS3 in peri-infarct cortical tissue.* n* = 4. (K) Western blotting analysis of DHRS3 protein expression in mitochondria of peri-infarct cortical tissue. *n* = 3. (L) Representative immunofluorescence images of neuron staining with DHRS3 and quantitative analysis of DHRS3^+^ NeuN^+^ cells in the peri-infarct region of the ischemic rats. *n* = 3. (M, N) Knocking down DHRS3 reduced the area of cerebral infarction and improved neurological function impairment. *n* = 8. Results are expressed as mean ± SD.^ #^*P <* 0.05, ^##^*P <* 0.01, ^###^*P <* 0.001 vs. CN or Sham group, ^*^P < 0.05, ^**^P < 0.01, ^***^P < 0.001 vs. OGD/R or tMCAO group. B, D and J-M, Statistical differences among groups were analyzed by using one-way ANOVA followed by Tukey's post-hoc test. F-I, Statistical differences among groups were analyzed by using two-way ANOVA followed by Tukey's post-hoc test. N, Behavior data were analyzed using non-parametric Mann Whitney test.

Despite weak binding affinity to the Keap1 BTB domain, Cpd.51 exhibited enhanced Nrf2 activation *in vitro* and *in vivo* relative to Oma. This discrepancy implied that Cpd.51 might engage non-canonical Nrf2 activation mechanisms. In order to further explore the neuroprotective mechanisms of Cpd.51, we performed RNA-seq on SH-SY5Y cells subjected to three conditions: (1) untreated controls (CN), (2) under OGD/R injury, (3) OGD/R with Cpd.51 treatment. Hierarchical clustering and volcano plot analysis revealed that compared to the CN group, OGD/R induced significant transcriptomic alterations, upregulating 179 genes and downregulating 54 genes versus, while Cpd.51 treatment upregulated 98 genes and downregulated 89 genes compared to the OGD/R group (Figure S5A). Intersection analysis identified 11 OGD/R-downregulated genes that were significantly rescued by Cpd.51 upregulation, and 26 OGD/R-upregulated genes suppressed by Cpd.51 (Figure S5B). From these, we validated seven Cpd.51-upregulated and nine Cpd.51-downregulated genes exhibiting the most pronounced fold-changes and functional relevance to neuroprotection. Notably, DHRS3 emerged as the top Cpd.51 target under both physiological and pathological conditions (Figure 6A, S5C-F). Consistent with the transcriptomic findings, OGD/R significantly upregulated DHRS3 protein expression, whereas Cpd.51 treatment substantially attenuated this induction (Figure 6B). This finding implicated DHRS3 as a potential mediator of Cpd.51-induced neuronal survival.

While DHRS3 is key for retinoic acid catabolism, and retinoic acid itself has demonstrated neuroprotective effects in AIS models, the direct relationship between DHRS3 and stroke has not been investigated 17, 29. Following this discovery, we focused on elucidating the mechanistic involvement of DHRS3 in the treatment of AIS. Subcellular localization study confirmed DHRS3 co-localization with mitochondrial marker in SH-SY5Y cells (Figure 6C). Cpd.51 treatment significantly reduced mitochondrial DHRS3 levels in OGD/R-injured SH-SY5Y cells (Figure 6D). To further validate whether DHRS3 participated in the neuroprotective effect of Cpd.51 and its improvement of mitochondrial function, an overexpression plasmid of DHRS3 was used in the subsequent experiments (Figure S5G). Transfection with DHRS3 overexpression plasmid significantly attenuated Cpd.51-induced cytoprotection, elevating the EC_50_ from 52.96 nM to 182.81 nM (Figure 6E). Furthermore, DHRS3 overexpression abrogated the mitochondrial protective effects of Cpd.51, as demonstrated by increased permeability (Figure 6F), elevated mitochondrial ROS production (Figure 6G), reduced mitochondrial membrane potential (Figure 6H), and suppressed PINK1 and TFAM mRNA (Figure S5H) and protein expression (Figure 6I).

*In vivo* studies revealed that tMCAO significantly increased DHRS3 expression in the cortical area surrounding the cerebral infarction, while Cpd.51 attenuated this effect (Figure 6J). The expression pattern of DHRS3 in mitochondria is similar to that in cortical tissue overall, and Cpd.51 significantly reduced DHRS3 expression in cortical mitochondria in the penumbral region following cerebral infarction (Figure 6K). Immunofluorescence analysis further revealed that DHRS3 was markedly elevated in neurons of the peri-infarct cortex following I/R injury, suggesting its potential involvement in neuronal damage pathogenesis (Figure 6L). Critically, Cpd.51 suppressed this neuronal DHRS3 upregulation. Next, we employed AAV-shDHRS3 to knock down DHRS3 expression in rat brain to validate the ameliorative effect of reduced DHRS3 expression on AIS (Figure S5I). Similar to expectations, the reduction in DHRS3 expression significantly reversed the increase in cerebral infarction volume and neurobehavioral deficits caused by tMCAO in rats (Figure 6M, 6N). These results collectively indicated that DHRS3 inhibition might constitute a key mechanism underlying the neuroprotective effects of Cpd.51, and moreover, establish a previously unrecognized functional link between DHRS3 and AIS pathology.

### Cpd.51 reverses DHRS3's inhibitory effect on Nrf2 binding to the ARE element by binding to DHRS3

**Figure 7:**
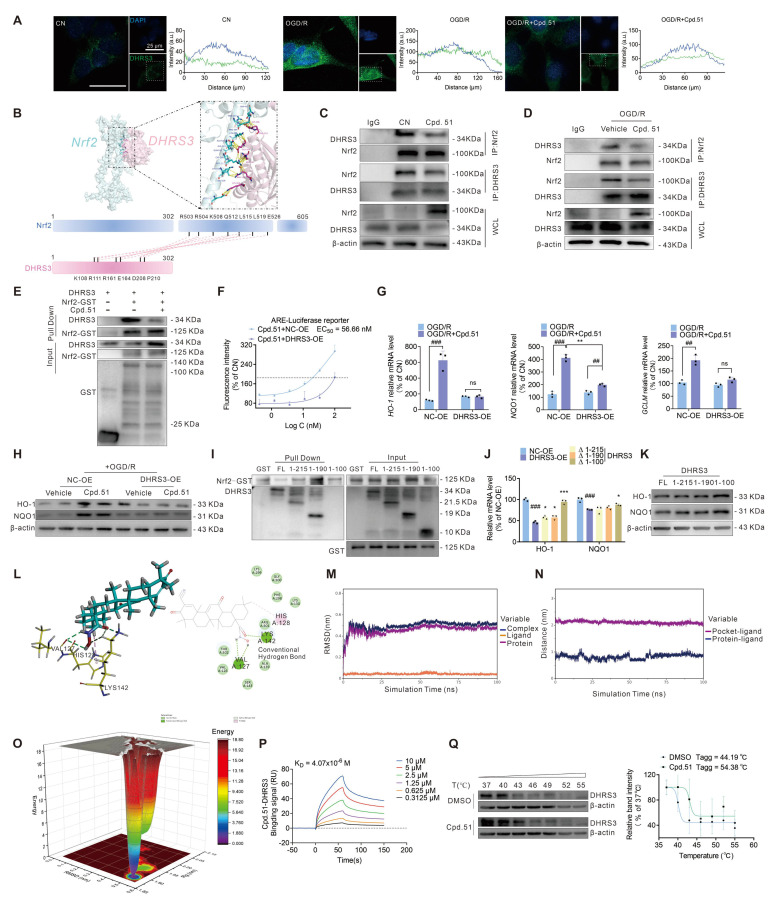
** DHRS3 bound to Nrf2 and inhibited its binding to the ARE element, which was inhibited by Cpd.51.** (A) Co-staining with DHRS3 and DAPI in SH-SY5Y cells demonstrated that OGD/R increased nuclear localization of DHRS3, which Cpd.51 effectively inhibited. (B) Three-dimensional structural models of DHRS3 and Nrf2 proteins, as well as the specific binding interfaces mediating their direct interaction, were generated using molecular docking approaches. (C, D) Immunoprecipitation assay of the interaction in whole cell lysate between Nrf2 and DHRS3 in physiological or OGD/R conditions, and SH-SY5Y cells treated with Cpd.51 (100 nM) or Vehicle (DMSO). *n* = 3. (E) Cpd.51 inhibited the interaction between Nrf2 and DHRS3 detected by GST pulldown. (F) DHRS3-overexpression inhibited the activation effect of Cpd.51 on the ARE luciferase reporter gene. *n* = 6. (G, H) DHRS3-overexpression suppressed the expression of downstream target gene and protein of Nrf2. *n* = 3 or 4. (I) The GST pull-down assay demonstrated that Nrf2 specifically bound to the region of DHRS3 encompassing amino acids 100 to 190. (J, K) Overexpression of DHRS3 inhibited the expression of downstream target genes and proteins of Nrf2, except for DHRS3 (1-100 aa). *n* = 3. (L) Three-dimensional and two-dimensional structural models of Cpd.51 and DHRS3 proteins, as well as the specific binding interfaces mediating their direct interaction, were generated using molecular docking approaches. (M) RMSD of complexes, proteins and small molecule ligands. (N) The distance between the binding sites of proteins and small molecules. (O) Free Energy Landscape. (P) SPR was employed to determinate of the binding affinity between Cpd.51 and DHRS3. (Q) CETSA analysis of intracellular binding between Cpd.51 (100 nM) and DHRS3 at different temperature.* n* = 3. Results are expressed as mean ± SD. J, ^###^*P <* 0.001 vs. NC-OE group. ^*^P < 0.05, ^***^P < 0.001 vs. DHRS3-OE group. Statistical differences among groups were analyzed by using one-way ANOVA followed by Tukey's post-hoc test. G, ^##^*P <* 0.01, ^###^*P <* 0.001. ^*^P < 0.05, ^**^P < 0.01, ^***^P < 0.001. Statistical differences among groups were analyzed by using two-way ANOVA followed by Tukey's post-hoc test.

The above results clearly demonstrated that DHRS3 was involved in the anti-AIS process of Cpd.51. However, these findings still failed to explained why Cpd.51 exhibits weak binding affinity for the Keap1 protein yet possessed potent Nrf2 activation activity. Immunofluorescence analysis revealed that OGD/R stimulation significantly promoted nuclear translocation of DHRS3, whereas Cpd.51 treatment attenuated this translocation (Figure 7A). This observation prompted us to investigate potential protein-protein interactions between DHRS3 and Nrf2, particularly within the nuclear compartment. We performed a complex prediction using AlphaFold 3 for the full-length Nrf2 (Uniprot ID: Q16236) and DHRS3 (Uniprot ID: O75911). Nrf2-DHRS3 protein-protein docking model simulations identified potential interaction sites between them, indicating that the binding site was located at DHRS3 (ASP208, PRO210, GLU164, ARG161, ARG111, LYS108) and Nrf2 (ARG503, ARG504, LYS508, GLN512, ARG515, LEU519, GLU526) (Figure 7B). To determine whether Cpd.51 modulated the Nrf2-DHRS3 interaction under physiological and OGDR conditions, we performed co-immunoprecipitation (Co-IP) assays in SH-SY5Y cells, and demonstrated that Cpd.51 substantially reduced DHRS3-Nrf2 complex formation under both physiological and OGD/R conditions (Figure 7C, 7D, S6A-F). To determine whether this interaction involves direct physical binding, we performed GST pull-down assays using purified GST-tagged Nrf2 and DHRS3 proteins. Results confirmed a direct DHRS3-Nrf2 interaction, and this binding affinity was significantly reduced by Cpd.51 (Figure 7E).

Collectively, these data supported a model wherein AIS-induced nuclear translocation of DHRS3 directly bound to Nrf2, thereby inhibiting Nrf2-dependent transcriptional activation of downstream antioxidant genes and compromising mitochondrial protection. To validate this hypothesis, we first assessed the impact of DHRS3 overexpression on ARE-driven transcriptional activity. As shown in Figure S6G, DHRS3 overexpression significantly suppressed ARE luciferase intensity relative to the control group, reducing it to <1% of baseline levels. This striking phenomenon suggested that DHRS3 exerted a significant negative regulatory effect on Nrf2 activity. Consistent with this, DHRS3 overexpression attenuated Cpd.51-induced enhancement of ARE-Luc fluorescence intensity (Figure 7F). Concordantly, DHRS3 overexpression suppressed Cpd.51-mediated upregulation of Nrf2 downstream targets (HO-1, NQO1 at mRNA/protein levels; *GCLM* at mRNA level) (Figure 7G, 7H, S6H). These results suggested that Cpd.51 attenuated the negative regulation of Nrf2 by DHRS3.

The NCBI database (https://uud.ncbi.nlm.nih.gov/home/genes/) identifies the 100-280 region of DHRS3 as the peptide-binding interface, which is consistent with our simulation results (Figure 7B). In an effort to confirm that the Nrf2-binding site was localized predominantly within residues 108-210 of DHRS3, we designed a set of DHRS3 fragment (1-215 aa, 1-190 aa, and 1-100 aa) for subsequent GST pull-down analysis. We observed a marked decrease in Nrf2 binding to the DHRS3 fragment (1-100 aa), while the DHRS3 fragment (1-215 aa) and DHRS3 fragment (1-190 aa) retained binding comparable to the full-length protein. This identifies the 100-190 region as essential for the interaction, which aligns with our predicted binding site (Figure 7I). To determine the effect of DHRS3 overexpression at different fragments on Nrf2's ARE-promoting activity, we overexpressed Nrf2 in Hek-293T cells (Figure S6I) and then separately transfected plasmids containing different DHRS3 fragments (Figure S6J). A DHRS3 (1-215 aa, 1-190 aa) over expression significantly inhibited the expression of downstream Nrf2 targets, a finding that aligned with the results obtained from the full-length DHRS3 overexpression plasmid (Figure 7J, 7K and S6K). Notably, the overexpression plasmid of the DHRS3 fragment (1-100 aa) continued to sustain a high level of expression of downstream Nrf2 gene/proteins (Figure 7J, 7K and S6K). This finding confirmed that Nrf2 binding to the DHRS3 (100-190 aa) region functions to inhibit Nrf2 transcriptional activity, thereby identifying this segment as a key regulatory domain.

In investigating the mechanism by which Cpd.51 impaired the DHRS3-Nrf2 interaction, we conducted molecular docking, and identified multiple binding sites between the structurally modified portion of Cpd.51 and DHRS3 (Figure 7L). Molecular dynamics simulations revealed stable binding of the small molecules to the target proteins. Analysis of the binding energy indicated that van der Waals interactions were the dominant stabilizing force, while electrostatic and hydrophobic contributions were secondary (Figure 7M-O). SPR analysis confirmed a strong binding affinity between Cpd.51 and DHRS3 (Figure 7P). Moreover, we were pleasantly surprised to discover that the binding mechanism between Cpd.51 and DHRS3 differed entirely from that of Keap1. High concentrations of Cpd.51 exhibited a robust bond with DHRS3, showing no signs of dissociation over time. Consistent with this result, CETSA demonstrated that Cpd.51 significantly enhances the thermal stability of DHRS3, providing orthogonal validation of the direct target engagement (Figure 7Q). In summary, Cpd.51 may form a robust interaction with DHRS3, with the binding site located at the interface between DHRS3 and Nrf2, thereby disrupting the interaction between DHRS3 and Nrf2.

Collectively, our findings demonstrated that Cpd.51 activated Nrf2 through dual mechanisms: (1) allosteric modulation of Keap1 by targeting Gly148 and Cys151 residues, which disrupted Keap1-mediated Nrf2 ubiquitination, and (2) inhibition of the direct DHRS3-Nrf2 protein interaction, thereby promoted the activation of the downstream targets of Nrf2.

### Cpd.51 enhances the inhibitory effect of Nrf2 on DHRS3 expression

**Figure 8:**
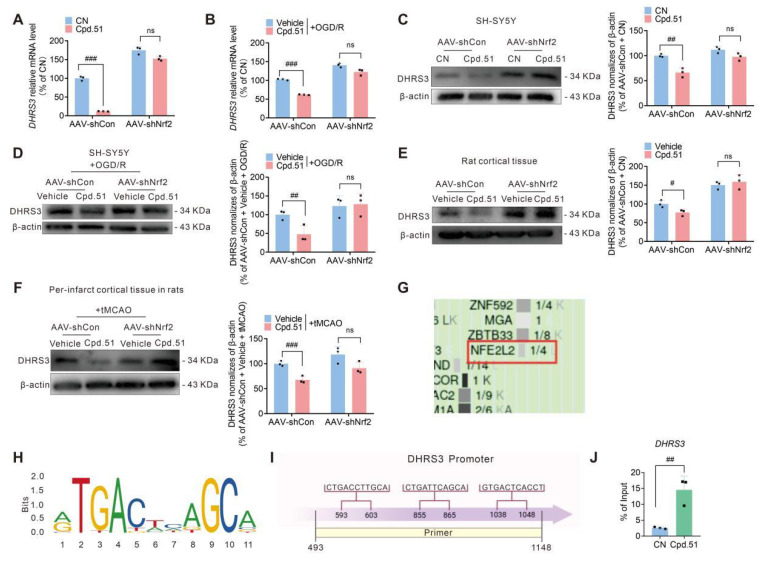
** Cpd.51 promoted Nrf2 binding to the DHRS3 promoter region, thereby inhibiting DHRS3 mRNA and protein expression.** (A, B) Nrf2-knockdown suppressed mRNA expression levels of DHRS3 in SH-SY5Y cells under physiological or OGD/R condition.* n* = 3. (C, D) Nrf2-knockdown suppressed DHRS3 protein expression in SH-SY5Y cells with physiological or OGD/R. *n* = 3. (E, F) Nrf2-knockdown suppressed DHRS3 protein expression in the cortex of rats under physiological or in the cortex surrounding the infarct area of rats with tMCAO. *n* = 3. (G) The UCSC Genome Browser database showed that Nrf2 might exert transcriptional regulation on DHRS3 expression. (H) The JASPAR database showed the sequence of the Nrf2 transcription factor DNA, which bound to the DHRS3 promoter. (I) The position of the ChIP primer in the DHRS3 promoter. (J) ChIP assay combined with qRT-PCR was performed using the Nrf2 antibodies in SH-SY5Y cells. *n* = 3. Results are expressed as mean ± SD. A-F, ^#^*P <* 0.05, ^##^*P <* 0.01, ^###^*P <* 0.001. Statistical differences among groups were analyzed by using Two-way ANOVA followed by Tukey's post-hoc test. J, ^##^*P <* 0.01 vs. CN group. Statistical differences among groups were analyzed by using Student's t test.

The UniProt database (https://www.uniprot.org/) identified Lys142 as the principal ubiquitination site on DHRS3. Should Cpd.51 bind at Lys142, it would be predicted to increase DHRS3 stability by preventing its ubiquitin-mediated degradation. However, our dates showed that Cpd.51 significantly suppressed DHRS3 expression at both transcriptional and protein levels. This discrepancy indicated that direct blockade of Lys142 ubiquitination was unlikely to be the predominant regulatory mechanism of Cpd.51, suggesting instead the involvement of alternative or additional pathways in modulating DHRS3. Given the established role of Cpd.51 as a potent Nrf2 activator, we investigated whether Nrf2 signaling mediated its suppression of DHRS3. Notably, Nrf2 knockdown markedly reversed Cpd.51-induced downregulation of *DHRS3* mRNA in SH-SY5Y cells under both physiological and OGD/R conditions (Figure 8A, 8B). Concordantly, the inhibitory effect of Cpd.51 on DHRS3 protein expression in SH-SY5Y cells in a physiological or OGD/R state (Figure 8C, 8D). Similar conclusions were also obtained in normal rat cortex and the peri-infarct cortex of tMCAO rats was abrogated by Nrf2 silencing (Figure 8E, 8F).

To elucidate the mechanistic basis of this regulation, we examined direct transcriptional control of DHRS3 by Nrf2. Firstly, we analyzed potential transcription factors regulating DHRS3 using the UCSC Genome Browser database. The results indicated that Nrf2 might exert transcriptional regulation on DHRS3 expression (Figure 8G). Secondly, bioinformatics analysis via JASPAR (https://jaspar.genereg.net) predicted three ARE motifs within the DHRS3 promoter (from + 493 bp to + 1148 bp) (Figure 8H, 8I). Chromatin immunoprecipitation (ChIP) assay confirmed Nrf2 occupancy at the DHRS3 promoter (Figure 8J), indicating that Nrf2 directly suppressed DHRS3 transcription through ARE binding.

### Stability, druggability, and toxicity analysis of Cpd.51

The toxicity of Oma primarily stems from off-target effects due to electrophilic reactions with proteins containing active cysteine residues. Our results indicated that Cpd.51 exhibited weak binding affinity to Keap1 and displayed reversible characteristics. This suggested that the reduced binding capability of Cpd.51 to active cysteine residues might lower its potential for toxic effects.

To assess acute toxicity, compounds were administered intravenously at a daily dose of 10 mg/kg for 7 days. Both Oma and Cpd.51 suppressed weight gain in rats (Figure S7A, S7B). However, rats treated with Oma showed marked liver hypertrophy and elevated serum levels of glutamic-pyruvic transaminase (GPT) and glutamic oxaloacetic transaminase (GOT), indicating hepatic injury. In contrast, these signs of hepatotoxicity were not observed in the Cpd.51-treated group (Figure S7C, S7D). Furthermore, Oma administration increased serum creatinine (CRE) and blood urea nitrogen (BUN) levels, suggesting renal impairment, although kidney volume remained unchanged (Figure S7E). Cpd.51 did not induce these changes. Collectively, these findings demonstrate that Oma induced both hepatic and renal toxicity, whereas Cpd.51 exhibited a superior safety profile with no observed organ damage.

Secondly, the long-term toxicity of Oma and Cpd.51 was evaluated over a 28-day period in accordance with FDA guidelines. Rats received daily intragastric administrations of Oma (10 mg/kg) or Cpd.51 (1, 3, and 10 mg/kg). Although neither compound significantly affected body weight (Figure S8A, S8H), Oma markedly increased the liver and kidney indices in both female and male rats (Figure S8B-E, S8I-L). Consistent with organ index results, Oma significantly elevated plasma markers of hepatic and renal injury, whereas Cpd.51 showed no such effects at any dose tested (Figure S8F-G, S8M-N).

Thirdly, we hypothesized that the lipophilic nature of Oma contributes to its accumulation in tissues such as the liver and kidneys, potentially underlying its toxicity. To test whether the structural modification in Cpd.51, replacing carbonyl and amino groups with hydroxyl groups, could reduce this accumulation, we performed tissue distribution analysis after the 28-day toxicity study. As shown in Figure S8O, Cpd.51 did not exhibit significant accumulation in the liver or kidneys compared to Oma, despite its lower plasma concentration. Pharmacokinetic data from a single oral dose revealed that Cpd.51 has an oral bioavailability of only 11.5% (Table S1). However, following intravenous administration, Cpd.51 showed relatively effective distribution to brain tissue (Table S2).

Finally, the results of *in vitro* hepatic microsomal stability indicated that Cpd.51 exhibited moderate metabolic stability in microsomes (Table S3). Therefore, we proceeded to analyze the metabolites of Cpd.51. Cpd.51 was transformed into 10 major metabolites in this study, and the biotransformations occurred include desaturation, hydrogenation, dimerization, and others (Table S4).

## Discussion

Acute ischemic stroke (AIS) is a common cerebrovascular disease characterized by high incidence and disability rates, placing a significant burden on global healthcare systems 49. At present, there are no high-efficiency therapeutic drugs for AIS in clinical treatment, intravenous thrombolysis and mechanical thrombectomy are still the main treatment methods 50, 51. Intravenous thrombolysis is limited in its therapeutic opportunities due to the time window and the risk of fatal complications, while mechanical thrombectomy has drawbacks such as high operational difficulty, device limitations, and a high risk of complications, resulting in a limited number of patients receiving treatment 50, 51. The therapeutic limitations of current strategies necessitate novel neuroprotective agents, and Nrf2 activation emerges as a promising approach due to its ability to mitigate ischemia-reperfusion injury via functioning as a master transcriptional regulator orchestrating cellular defense against oxidative stress by binding to antioxidant response elements (ARE) and driving the expression of cytoprotective genes involved in redox homeostasis, detoxification, neuroinflammation, ferroptosis and mitochondrial integrity maintenance 6, 52, 53.

Due to the cell-protective effects, several Nrf2 activators have been identified. One class of these agents belongs to electrophilic activators, which interfere with the ubiquitination process of Nrf2 by covalently modifying the cysteine 151 site of the Keap1 protein 25, 54. However, their reactive electrophilic motifs non-specifically adduct cysteine residues in off-target proteins, provoking dose-limiting toxicities such as hepatorenal damage 55. In contrast, direct competitive inhibitors of the Keap1-Nrf2 protein-protein interaction (PPI) avoid covalent modifications by sterically blocking the Keap1 Kelch domain. Despite their improved specificity, these compounds typically exhibit high molecular weights and poor blood-brain barrier (BBB) penetration, limiting their utility for central nervous system disorders 56, 57. Consequently, no direct Nrf2 activators have achieved clinical approval to date, underscoring the unmet need for CNS-penetrant, non-electrophilic Nrf2 therapeutics. Therefore, developing safer and more effective Nrf2 activators for clinical use remains a challenge.

In this study, we initially planned to synthesize a series of compounds that mitigated hepatotoxicity while ensuring Nrf2 activation. Docking results indicated that the 2,2-difluoropropionamide group at position C17 oriented toward the solvent-exposed region and maintained a considerable distance from the key binding site of Keap1. This suggested that structural modification at this site would likely be tolerated without substantial loss of activity. Consequently, we first considered replacing the hydrophobic fluorine and methyl substituents with more polar groups to reduce lipophilicity. An initial design involved introducing a carboxyl group at this position. However, this strategy was abandoned due to the potential risk of impaired blood brain barrier penetration associated with carboxyl moieties. Further analysis indicated that the C17 amide functionality does not participate in critical interactions with Keap1. We therefore modified this site by sequentially replacing the carbonyl and amino groups and subsequently introducing a hydroxyl functionality, leading to the design of a hydroxylamine derivative.

To ensure the safety of Cpd.51, we conducted a comprehensive assessment comparing its toxicity profile with that of Oma, evaluating both acute toxicity and long-term safety. FDA nonclinical reviews indicate that Oma achieves significant brain penetration post-oral administration, with a half-life ranging from 6 to 30 h 58. However, the FAD report and research papers indicated that 28-day toxicity testing in rats induced hepatotoxicity with oral administration of 10 mg/kg, as evidenced by increased liver weight and elevated plasma alanine aminotransferase (ALT) levels 59. In contrast, our data demonstrated that Cpd.51 maintained comparable brain penetration, with an oral half-life of 6.36 h, yet did not induce hepatomegaly or elevate serum glutamate-pyruvate transaminase (GPT)/glutamic-oxaloacetic transaminase (GOT) (equivalent to ALT) levels in rat models. More importantly, our therapeutic dose was only half of the toxicity test dose, which better ensured the safety of Cpd.51 in the treatment of AIS. Collectively, these findings suggested that Cpd.51 retained brain bioavailability while mitigating hepatotoxicity relative to Oma. The tissue distribution profile revealed that Cpd.51 exhibited a higher propensity for hepatic accumulation compared to Oma. Correspondingly, the reduction in its liver toxicity was likely attributable to the introduced structural modification.

We assessed the activation of Nrf2 by Cpd.51 using an ARE luciferase reporter assay and an Nrf2 sensor system. The results demonstrated that Cpd.51 enhanced fluorescence intensity with a lower EC_50_ compared to Oma. At 100 nM, Cpd.51 exhibited a trend toward increased fluorescence intensity. Further, Nrf2 sensor assay showed that Cpd.51 significantly inhibited Nrf2 clearance with an EC_50_ at 4.02 nM. These data all indicate that Cpd.51 still retains the desirable Nrf2 activation after our structural modification. Surprisingly, in the rat tMCAO model, Cpd.51 significantly reduced the infarct volume, improved neurobehavioral scores, and demonstrated superior efficacy compared to Oma. In peri-infarct area of cortical tissue, Cpd.51 significantly promoted the expression of Nrf2 and its downstream genes HO-1, NQO1, and GSH, while reducing MDA expression. These results suggested that Cpd.51 promoted the activation of Nrf2 *in vivo* and exerted an excellent protective effect against AIS injury. In addition, the positive effect of cpd51 has also been verified in the OGD/R model.

The pathological progression of AIS involves multicellular interactions within the neurovascular unit, driving ongoing research focused on microglial inflammation dynamics 35, 60, 61, neuronal survival mechanisms 62, 63, and BBB repair strategies 64, 65. Nrf2 exhibits cell type-specific expression patterns. Astrocytes maintain high basal Nrf2 levels, enabling critical regulation of glucose metabolism and antioxidant defense systems 66, 67. In contrast, neuronal Nrf2 expression is constitutively suppressed under physiological conditions due to Keap1-mediated ubiquitination, and ischemic injury triggers Nrf2 nuclear translocation in neurons 8, 68. Similarly, microglia respond to pathological stimuli by adjusting Nrf2 activity 69. Consequently, the cellular targets of Cpd.51 were further investigated. *In vivo* immunofluorescence and *in vitro* experiments on SH-SY5Y cells and primary cortical neurons indicated that Cpd.51 selectively activates Nrf2 in neurons. Even at low concentrations, Cpd.51 could also specifically facilitate the nuclear translocation of Nrf2 in neurons as opposed to glial cells. Furthermore, the enhanced upregulation of Nrf2 downstream protein expression in neurons compared to glial cells. The neuron-specific activation of Nrf2 by Cpd.51 might explain its enhanced protective effect on neurons after OGD/R. To determine whether Cpd.51's neuroprotective effects depend on Nrf2 activation rather than off-target mechanisms, Nrf2-knockout models were established via AAV-shNrf2 administration. The ameliorative effect of Cpd.51 on brain injury in tMCAO rats and its neuroprotective action were significantly reversed by Nrf2 depletion, which was consistent with the results obtained *in vitro*, confirming that Cpd.51-mediated neuroprotection requires Nrf2 activation in neurons.

Mitochondria-targeted therapeutics represent a prominent research focus in cerebral ischemia intervention, with neuroprotective mechanisms centered on mitochondrial functional restoration, oxidative stress mitigation, and apoptosis suppression. Idebenone, which enhances mitochondrial electron transport chain activity and demonstrates therapeutic potential in ischemia-associated neurological disorders 70, 71. Cyclosporine A, which attenuates mitochondrial permeability transition pore (MPTP) over-activation, thereby reducing ROS generation and neuronal/microglial apoptosis following ischemia/reperfusion injury 72. Traditional Chinese medicine (TCM) and phytochemical constituents further expand this therapeutic landscape through multi-target modulation of mitochondrial dysfunction. TCM-based strategies align with the pathophysiological complexity of post-ischemic mitochondrial damage via synergistic regulation of redox homeostasis, bioenergetic recovery, and death signaling pathways 73. Crucially, targeting the transcription factor Nrf2, rather than mitochondrial components directly, provides a more integrative approach to mitochondrial protection 74, 75. Nrf2 activation transcriptionally coordinates *de novo* antioxidant synthesis, mitochondrial biogenesis, and quality control mechanisms, collectively preserving mitochondrial homeostasis after ischemic insult 76-78. Our findings indicated that Cpd.51 significantly ameliorated mitochondrial damage induced by AIS, and these effects were reversed by Nrf2 knockdown. Based on the above findings, we conclude that Cpd.51 mediated mitochondrial protection by targeting and activating Nrf2 in neurons.

To determine whether Cpd.51's mechanism resembles that of Oma, we performed molecular docking, surface plasmon resonance (SPR), and cellular thermal shift assays (CETSA) with Keap1 mutant plasmids (C151A, G148A, and C151A & G148A). These experiments revealed that Cpd.51 binds to Cys151 and Gly148 within the Keap1 BTB domain. However, Cpd.51 exhibited a higher dissociation constant (K_D_ = 53.9 μM) when bound to full-length Keap1 protein. Although its binding affinity improved for the Keap1 BTB domain fragment (K_D_ = 31.3 μM), this value remained significantly higher than the K_D_ of Nrf2-Keap1 binding (19 nM) 79, and higher than the K_D_ of Oma (21.7 nM). Collectively, these findings indicated that Cpd.51 activated Nrf2 through a distinct mechanism from Oma.

Given Cpd.51's low affinity for the Keap1 protein and its excellent Nrf2 activation effects, we employed multiple approaches, including transcriptomics, overexpression plasmids, ChIP assays, Co-IP, and Pull down, to analyze other potential targets regulated by Cpd.51. Among candidate targets, short-chain dehydrogenase/reductase family member 3 (DHRS3) emerged as the most significantly regulated by Cpd.51 and demonstrated the strongest functional association with mitochondrial repair and Nrf2 pathway modulation. DHRS3, a conserved metabolic enzyme within the SDR superfamily, catalyzes the reduction of retinaldehyde to retinol, thereby limiting retinoic acid synthesis, thereby participating in multiple pathological processes such as BBB disruption and inflammatory responses 16, 17. In amyotrophic lateral sclerosis models, DHRS3 upregulation synergistically correlates with disease progression and exacerbates neuronal degeneration through activation of the complement cascade in the immune system 80. During tumorigenesis and progression, the expression levels and activity of DHRS3 are regulated by epigenetic mechanisms, while its abnormal expression is associated with various cancers 13, 19, 81. DHRS3 forms a retinoid oxidoreductase complex (ROC) with retinaldehyde dehydrogenase 10 (RDH10), dynamically balancing retinaldehyde and retinol interconversion to govern retinol storage in lipid droplets 12, 82, 83. In this study, we discovered that reducing DHRS3 by AAV led to a significant decrease in cerebral infarction size and an improvement in neurological function impairment. *In vitro* experiments revealed that overexpression of DHRS3 significantly reversed the protective effects of Cpd.51 on SH-SY5Y cells and mitochondria. The above findings highlight DHRS3 as a potential new therapeutic target for AIS.

Importantly, our data demonstrated a direct interaction between Nrf2 and DHRS3, confirmed at both the protein and cellular levels. To verify the functional consequence of this interaction on Nrf2 pathway activation, we employed a DHRS3 overexpression plasmid. Results showed that DHRS3 overexpression significantly attenuated the fluorescence intensity of the ARE luciferase reporter and effectively counteracted the enhancement induced by Cpd.51. Furthermore, the mRNA and protein levels of key downstream antioxidant genes were also downregulated following DHRS3 overexpression. Further investigation of the binding regions between Nrf2 and DHRS3 revealed that DHRS3 (1-190) and DHRS3 (1-215) exhibited binding activity with Nrf2, whereas DHRS3 (1-100) did not interact with the Nrf2 protein. These suggested that Nrf2 and DHRS3 predominantly interacted within the region of DHRS3 (100-190), aligning with our simulation findings. We found that Cpd.51 inhibited the interaction between Nrf2 and DHRS3 by directly binding to DHRS3, particularly affecting the DHRS3-Nrf2 protein-protein interaction interface. This disruption led to the dissociation of Nrf2 from DHRS3, consequently increasing the activation efficacy of Nrf2.

To further investigate the relationship between Nrf2 and DHRS3, as well as to elucidate the mechanism by which Cpd.51 downregulated DHRS3, we performed a chromatin immunoprecipitation (ChIP) assay. Our results indicated that Nrf2 directly bound to the DHRS3 promoter region. Cpd.51 treatment significantly enriched Nrf2-bound DNA fragments at DHRS3 regulatory sites. Typically, Nrf2 bound to the regulatory regions of target genes, thereby enhancing their transcription. However, Nrf2's role extended beyond this function. There existed an alternative regulatory mechanism that did not depend on AREs. Specifically, the activation of Nrf2 could impede the recruitment of RNA polymerase II (Pol II) to the target gene loci, consequently inhibiting the transcription of these genes 84. The regulation of DHRS3 by Nrf2 might occur through a mechanism that was independent of ARE, necessitating further experimental validation. Knockdown of Nrf2 in both *in vitro* and *in vivo* significantly upregulated DHRS3 mRNA and protein levels. Concurrently, Cpd.51-mediated suppression of DHRS3 was abolished in Nrf2 knock down system. These results demonstrated that Cpd.51 might have inhibited the expression of DHRS3 by activating Nrf2.

In summary, Cpd.51 activated Nrf2 through two pathways. It both bound to Keap1, reducing its mediation of Nrf2 ubiquitination, and inhibited the binding between Nrf2 and DHRS3, thereby alleviating DHRS3's negative regulation of Nrf2, thereby preserving mitochondrial function specifically in neurons and mitigating ischemic damage. Key findings include: (1) Nrf2 pathway activation: We confirmed the Nrf2-activating activity of Cpd.51 through ARE-luciferase reporter gene, Nrf2 fluorescent sensor system, the expression of downstream antioxidant elements (HO-1, NQO1, and CGLM), and oxidative indicators such as GSH and MDA. (2) Neuron-specific efficacy: Cpd.51 promoted dose- and time-dependent Nrf2 activation in neurons, attenuating ischemia-reperfusion injury, and these effects abrogated by Nrf2 knockdown. (3) Binding specificity to Keap1: Molecular docking, SPR of full-length/truncated Keap1, and CETSA with Cys151/Gly148/ mutants confirmed Cpd.51's selective engagement with Keap1 residues. (4) The relationship between DHRS3 and mitochondrial dysfunction after AIS: We discovered for the first time that reducing DHRS3 was beneficial for the recovery of AIS, by regulating mitochondrial function. (5) Interaction between Nrf2 and DHRS3: Cpd.51 reduced mitochondrial damage via Nrf2-mediated suppression of DHRS3 transcription and disruption of the Nrf2-DHRS3 protein complex. (6) Superior safety and pharmacokinetics: Cpd.51 exhibited favorable brain bioavailability, and compared with the marketed Nrf2 activator Oma, Cpd.51 was safer and did not induce liver or kidney damage.

Several limitations remain in the present study. First, since Cpd.51 was a derivative of Oma, we have only identified its binding interaction with residues Cys151 and Gly148 within the BTB domain of the Keap1 protein, while whether Cpd.51 interacted with other Keap1 regions remains unknown. Second, although we suggested that Cpd.51 specifically activated Nrf2 in neurons, the mechanism underlying this specificity remained unclear. Third, our data suggested that the hepatotoxicity of Cpd.51 was significantly lower than that of Oma, potentially due to its reduced binding affinity for Keap1. However, this hypothesis necessitates further experimental validation. Moreover, a comprehensive comparative toxicological profile, particularly regarding off-target effects, also requires further independent research. Nevertheless, our study identified DHRS3 as a novel druggable target that directly bound and inhibited Nrf2, revealing a new regulatory axis beyond Keap1 and opening paths for targeted neuroprotection in ischemic stroke and other oxidative diseases.

## Supplementary Material

Supplementary Document Part I.

Supplementary Document Part II.

## Figures and Tables

**Figure 9:**
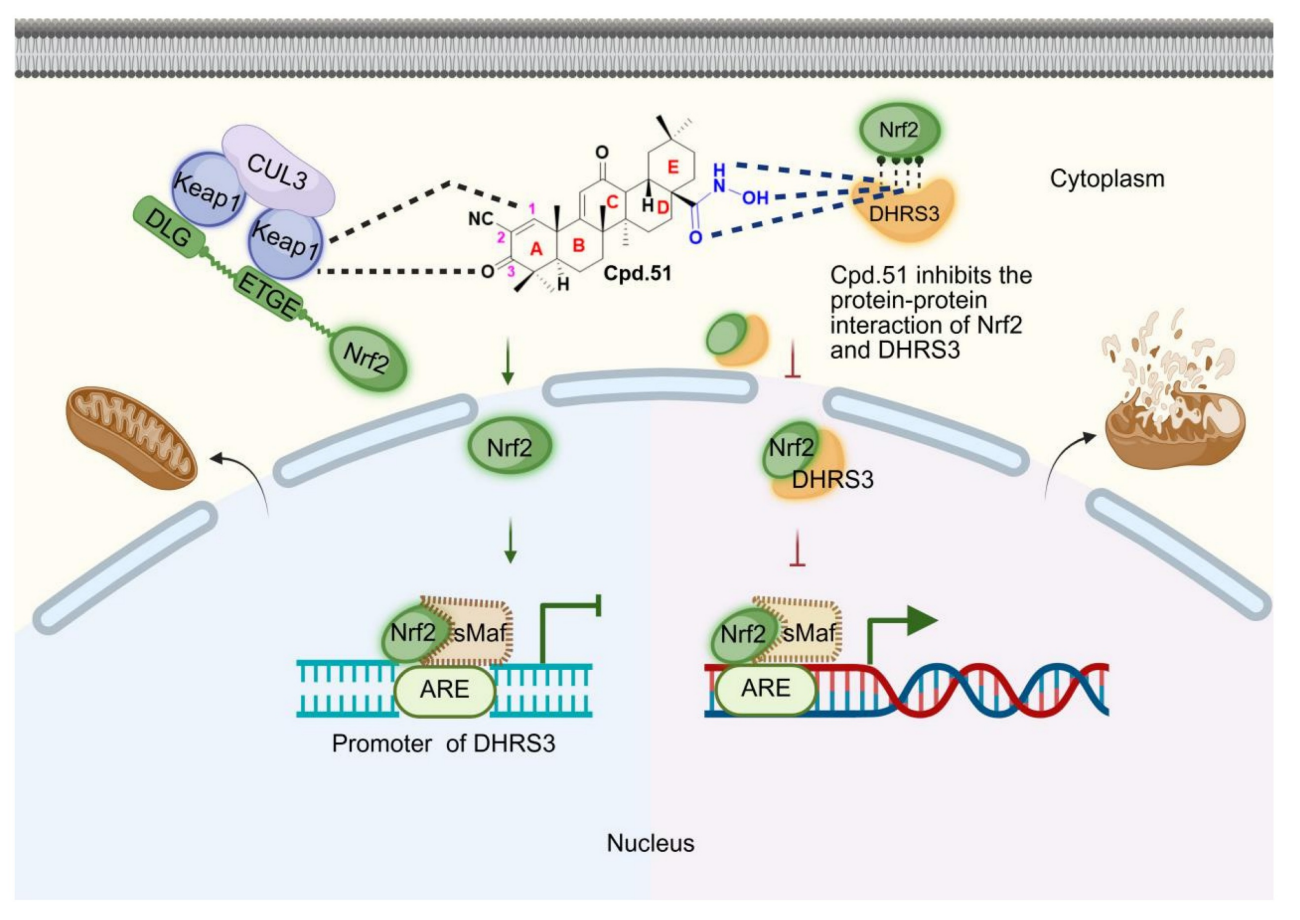
** Graphical abstract**.

## Abbreviations

AIS: acute ischemic stroke
ARE: antioxidant response elements
BUN: blood urea nitrogen
CCK-8: cell counting kit-8
CRE: creatinine
Cpd.51: Compound 51
DALYs: disability-adjusted life years
DMF: dimethyl fumarate
DHRS3: short-chain dehydrogenase/reductase family member 3
FDA: Food and Drug Administration
GPT: glutamic-pyruvic transaminase
GOT: glutamic oxaloacetic transaminase
I/R: ischemia/reperfusion
Nrf2: nuclear factor erythroid 2-related factor 2
Oma: Omaveloxolone
OGD/R: oxygen glucose deprivation/reperfusion
PPI: protein-protein interaction
ROS: reactive oxygen species
rt-PA: recombinant tissue-type plasminogen activator
Keap1: Kelch-like ECH-associated protein 1
K_D_: equilibrium dissociation constant value
RA: retinoic acid
sMAF: small MAF proteins
SPR: surface plasmon
