# Supplementary material for: Discovery of a novel Nrf2 activator that modulates mitochondrial function in neurons by regulating DHRS3-Nrf2 interaction after ischemic stroke
Source: Theranostics. 2026 Mar 30;16(10):5713–40. doi: 10.7150/thno.128602 (PMC13081163; doi:10.7150/thno.128602)
Supplement: Supplementary file 1 — Supplementary Document Part I. [file thnov16p5713s1.pdf]

Supporting Information 1

Discovery of a novel Nrf2 activator that modulates mitochondrial function in neurons by regulating DHRS3-Nrf2 interaction after ischemic stroke

Authors

Xiaohui Sun[1#] , Zhaofeng Liu[1#] , Huanhuan An[1], Hengwei Xu[1,2], Fangxia Zou[1,2], Jing Lu[1,2], Xiaofan Zhang[1], Xinyu Han[1], Ziwei Song[1,2], Yanli Sun[3], Wenyan Wang[1,2], Hongbo Wang[1], Jianzhao Zhang[1,2], Yunjie Wang[1]*, Jingwei Tian[1,2]*

Affiliations

[1]School of Pharmacy, Key Laboratory of Molecular Pharmacology and Drug Evaluation (Yantai University), Ministry of Education, Collaborative Innovation Center of Advanced Drug Delivery System and Biotech Drugs in Universities of Shandong, Yantai University, Yantai, 264005, China

[2]State Key Laboratory of Advanced Drug Delivery and Release Systems, Shandong Luye Pharmaceutical Co., Ltd., Yantai, Shandong 264003, PR China

3School of Life Science and Technology, ShanghaiTech University, Shanghai 201210, China

*Correspondence: Yunjie Wang, wangyunjie@ytu.edu.cn; Jingwei Tian, tianjingwei618@163.com

[#] These authors contributed equally to this work

Part I: Synthesis of Omaveloxolone derivative Cpd.51

1. Synthesis of Compound 51

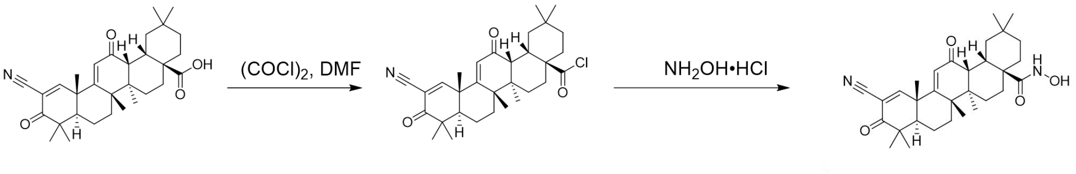

1 2 Cpd.51

Add N, N-dimethylformamide (7.43 mg, 101.70 μmol, 7.82 μL, 0.1 eq) and oxalyl chloride (387.25 mg, 3.05 mmol, 267.07 μL, 3 eq) to a solution of compound 1 (Bardoxolone, CAS No. : 218600-44-3, Purity: 99.50%, 500 mg, 1.02 mmol, 1 eq) in dichloromethane (8 mL). The reaction mixture was allowed to react at 25 °C for 1 h. LC-MS monitoring confirmed complete disappearance of starting materials and a major peak corresponding to the target product. The reaction mixture was concentrated under reduced pressure to afford compound 2 (500 mg, 980.19 μmol, 96.38% yield), a pale yellow solid. At 0 °C, compound 2 (500 mg, 980.19 μmol, 1 eq) was added to a solution of hydroxylamine hydrochloride (81.74 mg, 1.18 mmol, 1.2 eq) and N,N-diisopropylethylamine (380.04 mg, 2.94 mmol, 512.18 μL, 3 eq) in a 5 mL solution of dichloromethane. The reaction mixture was heated to 20 °C and reacted for 2 h. LC-MS monitoring indicated that the starting material was not completely consumed and product formation occurred. The reaction mixture was filtered and concentrated. The crude product was purified by reverse-phase preparation chromatography [water (formic acid)-acetonitrile system] and lyophilized to afford Compound 51 (136.20 mg, 266.39 μmol, 27.18% yield, 99.1% purity).

Prep-HPLC: column: Phenomenex luna C18 150*40 mm* 15 μm; mobile phase: [water (FA)-ACN]; gradient: 45%-75% B over 15 min.

LCMS: Rt = 1.339 min, 507.3 $[M+H]^+$ ESI pos.

HPLC: Rt = 1.816 min

^{1}H NMR: (400 MHz, CHLOROFORM-d) δ = 9.39 - 9.07 (m, 1H), 8.03 (s, 1H), 7.68(br s, 1H), 6.04 (s, 1H), 2.97 (d, J = 4.6 Hz, 1H), 2.92 - 2.84 (m, 1H), 2.01 - 1.92 (m, 1H), 1.85 - 1.79 (m, 1H), 1.78 - 1.71 (m, 4H), 1.70 - 1.62 (m, 3H), 1.57 - 1.45 (m, 4H), 1.42 (s, 3H), 1.31 (br d, J = 4.0 Hz, 1H), 1.28 (s, 3H), 1.26 - 1.23 (m, 1H), 1.21 (s, 3H), 1.12 (s, 3H), 0.97 (d, J = 5.8 Hz, 6H), 0.88 (s, 3H).

The relevant spectrogram report can be found in Supporting Information 2.

Part II: Supplementary tables

Table S1. Pharmacokinetics of Cpd.51 in male rats

Compound	C_0 (ng/mL)	C_{max} (ng/mL)	AUC_{0-t} (h*ng/mL)	C_L_obs (mL/h/kg)	$T_{1/2}$ (h)	MRT_{last} (h)	Vss_obs (mL/kg)	F (%)
Cpd. 51 (5 mg/kg, i.v.)	3410 ± 45	2117 ± 75	991 ± 70	5010 ± 354	1.49 ± 0.31	0.843 ± 0.054	4703 ± 299	/
Cpd. 51 (30 mg/kg, i.g.)	/	124 ± 37	685 ± 111	/	6.36 ± 2.32	5.17 ± 0.72	/	11.5 ± 1.9

Table S2. Cpd.51 is capable of crossing the blood-brain barrier

	Time (h)	Plasma (ng/mL)	Brain (ng/g)	Brain/Plasma
Cpd. 51 (5mg/kg, i.v.)	0.25	1287 ± 68	86.7 ± 49.3	0.07 ± 0.04
	1	198 ± 44	29.5 ± 17.5	0.16 ± 0.12
	4	39.5 ± 10.4	10.3 ± 4.0	0.28 ± 0.14

Table S3. Microsomal metabolic stability in liver microsomes from different species.

Compound	species	Remainig (%, t = 60 min)	$T_{1/2}$ (min)	CL_{int} (liver) mL/min/kg
51	Rat	51.1	60.8	41.0
	Dog	32.9	39.2	51.0
	Human	28.9	36.7	34.0

Table S4. Major metabolites of Cpd.51 incubated in human liver microsomes in the presence of NADPH

Peak ID	Found *m/z*	Mass Shift	Biotransformation	R.T. (min)	Human liver Relative	MS peak
Parent (T_0)	507.3223<br>505.3066	n/a	n/a	5.60	100.00%	1.19E+04<br>1.59E+04
Parent (T_5)	507.3223<br>505.3066	n/a	n/a	5.60	54.06%	6.69E+03<br>8.97E+03
M504	505.3064<br>503.2909	-2.0157	Desaturation	5.91	1.09%	1.11E+02<br>ND
M508	509.3382<br>507.3222	2.0156	Hydrogenation	5.32	11.61%	2.40E+03<br>2.59E+03
M522a	523.3130<br>521.2999	15.9933	Hydroxylation	4.33	+	6.00E+01<br>1.08E+02
M522b	523.3158<br>521.3034	15.9968	Hydroxylation	4.90	8.13%	7.86E+02<br>9.78E+02
M524a	525.3320<br>523.3166	18.0100	Hydroxylation + Hydrogenation	3.96	+	3.90E+01<br>5.00E+0
M524b	525.3320<br>523.3130	18.0064	Hydroxylation + Hydrogenation	4.29	+	1.00E+01<br>1.20E+0
M524c	525.3319<br>523.3155	18.0089	Hydroxylation + Hydrogenation	5.09	+	5.90E+01<br>1.00E+02
M537	538.3284<br>536.3135	31.0069	+ NHO	4.85	+	5.50E+01<br>4.30E+01
M538	539.3121<br>537.2973	31.9907	2 × Hydroxylation	3.49	0.69%	2.50E+01<br>4.10E+01
M1012	1013.633<br>1011.619	506.3124	Dimer	7.83	5.16%	7.87E+02<br>8.54E+02

For detailed data, please refer to Supplementary Material 2.

Table S5. Antibody information

Primary antibodies	Dilution rate	Manufacturer	Citation	Cat.no.
Nrf2	1 : 1000 / 1 : 100	Proteintech	WB/CO-IP	16396-1-AP
Nrf2	1 : 200	Cell Signaling	IF	D9J1B
Keap1	1 : 1000 / 1 : 100	Proteintech	WB/CO-IP	10503-2-AP
HO-1	1 : 3000	Proteintech	WB	66743-1-1g
NQO1	1 : 10000	Abcam	WB	AB80588
DHRS3	1 : 1000 / 1 : 300 / 1 : 100	Proteintech	WB/IF/CO-IP	15393-1-AP
PINK1	1 : 2000	Immunoway	WB	YM8583
TFAM	1 : 2000	Immunoway	WB	YM8380
V3DAC1	1 : 1000	Immunoway	WB	YM8582
NeuN	1 : 500	Serviebio	IF	GB120017-50
Iba-1	1 : 500	Abcam	IF	AB283319
GFAP	1 : 500	Abcam	IF	AB7260
β-tubulin	1 : 200	Abcam	IF	AB52623
Synaptophysin	1 : 300	ABclonal	IF	A6344
Alexa Fluor® 488-conjugated Goat Anti-Mouse IgG (H+L)	1 : 500	Serviebio	IF	GB25301
Cy3 conjugated Donkey Anti-Mouse IgG (H+L)	1 : 500	Serviebio	IF	GB21301
Goat Anti-Mouse IgG（H+L）HRP	1 : 5000	SparkJade	WB	EF0001
Goat Anti-Rabbit IgG(H+L) HRP	1 : 5000	SparkJade	WB	EF0002
β-actin	1 : 1000	Beyotime	WB	AF0003

Table S6. Primer information

Primer names	Forward	Reverse
CYP4F11	CATCTCCCGATGTTGCACG	TCTCTTGGTCGAAACGGAAGG
CFH	GTGAAGTGTTTACCAGTGACAGC	AACCGTACTGCTTGTCCAAAA
DPYS	ATTGATTTCGCCATTCCTCAGAA	GCTGTAGTCGCAGCAAACTTT
CLEC19A	TCCCTCTCAATAAGACCTGGG	AGTCCATTCAAACTGCCCTTC
KRT16	GACCGGCGGAGATGTGAAC	CTGCTCGTACTGGTCACGC
DNTT	TAGCAGAGAACAACTCGGGTT	CAGCCAGGAGACATCGAGGA
ARHGAP40	CTCGCTCAGTGCGAAGACAA	CATTCTCTGACGACATTTTCCC
HSPA6	CAAGGTGCGCGTATGCTAC	GCTCATTGATGATCCGCAACAC
GKN1	CTGTCCACTGCTTTCGTGAAG	GTCCCATCCGTTGTTATTGTCAA
DHRS3	ACTGAGTGCCATTACTTCATCTG	CATCACTGTCCATTAGGCTCTTC
CD300LB	GGTCCCTGACGGTTCAATG	GATGGACACACGGTCACTCTT
TNR	AAGAATTGCTCGGAGCCCTAC	GCTGTACTCGCTGTCACAGAT
BCL2A1	TACAGGCTGGCTCAGGACTAT	CGCAACATTTTGTAGCACTCTG
F2RL3	GCTGCTGCATTACTCGGAC	ACGTAGGCACCATAGAGGTTG
MT1M	GAGATCTCCAGCCTTACCGC	AGGAGCAGCAGCTCTTCTTG
GSTA3	GCAGCTGGAGTGGAGTTTGAA	AAAGCTTTTGCATCTGCGGG
GAPDH	GGAGCGAGATCCCTCCAAAAT	GGCTGTTGTCATACTTCTCATGG
TFAM	ATGGCGTTTCTCCGAAGCAT	TCCGCCCTATAAGCATCTTGA
PINK1	GCCTCATCGAGGAAAAACAGG	GTCTCGTGTCCAACGGGTC
Keap1	CTGGAGGATCATACCAAGCAGG	GGATACCCTCAATGGACACCAC
TFAM (rat)	GGCGTGCTAAGAACACTGGG	ACAGATAAGGCTGACAGGCGAG
PINK1 (rat)	CCATGGGCAGGAACACTATT	CCTACACACAGCCCTCACCT
GAPDH (rat)	GGTCGGAGTCAACGGATTTG	ATGAGCCCCAGCCTTCTCCAT

Table S7. Critical commercial assays information

Critical commercial assays	Manufacturer	Cat.no.
Bright-One Step Luciferase Assay Kit	Yeasen	11412ES81
ARE Luciferase Reporter Plasmid	Yeasen	11548ES03
Polyethylenimine Linear (PEI) MW40000	Yeasen	40816ES01
Lipofectamine™ 3000	Invitrogen	L3000075
Cell Counting Kit-8	Beyotime	C0039
ATP assay kits	Beyotime	S0027
Luminescence ATP Detection Assay System	PerkinElmer	0RT0769
GSH assay kit	Beyotime	S0053
MDA assay kit	Beyotime	S0131M
GPT assay kit	Mlbio	ml092635
GOT assay kit	Mlbio	ml092714
CRE assay kit	Mlbio	M12C4L
BUN assay kit	Mlbio	ml076479
BCA protein assay	Beyotime	P0012
ROS Assay Kit (DCFH-DA)	Beyotime	S0033S
JC-1 Assay Kit	Beyotime	C2003S
Cell Mitochondria Isolation Kit	Beyotime	C3601
SPARKeasy Ultra-Pure Total RNA Rapid Extraction Kit	Sparkjade	AC0103
SPARKscript II 1st Strand cDNA Synthesis Kit (With gDNA Eraser)	Sparkjade	AG0302-B
2×SYBR Green qPCR Mix (With ROX)	Sparkjade	AH0104-C
Lipofectamine™ 3000 and P3000™ reagent	Invitrogen	L3000015
Pierce™ Classic Magnetic Bead Method IP/Co-IP Kit	Thermo Scientific	88804
GST pull-downn Kit (Agarose)	Elabscience Biotechnology	EA-IP-K008
ChIP-IT High Sensitivity	Active Motif	53040

Table S8. Chemicals and recombinant proteins information

Chemicals and recombinant proteins	Manufacturer	Cat.no.
Opti-MEM	Gibco	51985034
Advanced MEM	Gibco	12492013
DMEM	Gibco	11965092
FBS	Gibco	A5256701
HMC3 Cell Complete Medium	Procell	CM-0620
CTX TNA2 Cell Complete Medium	Sunncell	SNLM-552
0.25% Trypsin digestive solution	ABclonal	BR00083
TTC	TCI	298-96-4
Poly-D-lysine	Solarbio	P2100
Serum-free neurobasal	Gibco	10888022
Glutamax	Gibco	35050061
B27 supplement	Gibco	17504044
Cytosine arabinoside	Sigma	V900339
Glutaraldehyde	Solarbio	P1126
PVDF membranes	Thermo Scientific	88520
Calcein-AM	Beyotime	Y237214-1mg
DAPI	Beyotime	C1006-200mL
Triton X-100	Beyotime	C1715-100mL
DMSO	Sigma	D8418
Solutol HS-15	MCE	61909-81-7
Omaveloxolone	MCE	1474034-05-3
Pronase	Merck	10165921001
Keap1 protein	Sinobiological	ME18DE0203
Nrf2 protein	Ybio	YB710012
DHRS3 protein	Ybio	YB765100
Recombinant GST Tag protein	Proteintech	Ag0040

Part III: Supplementary figures

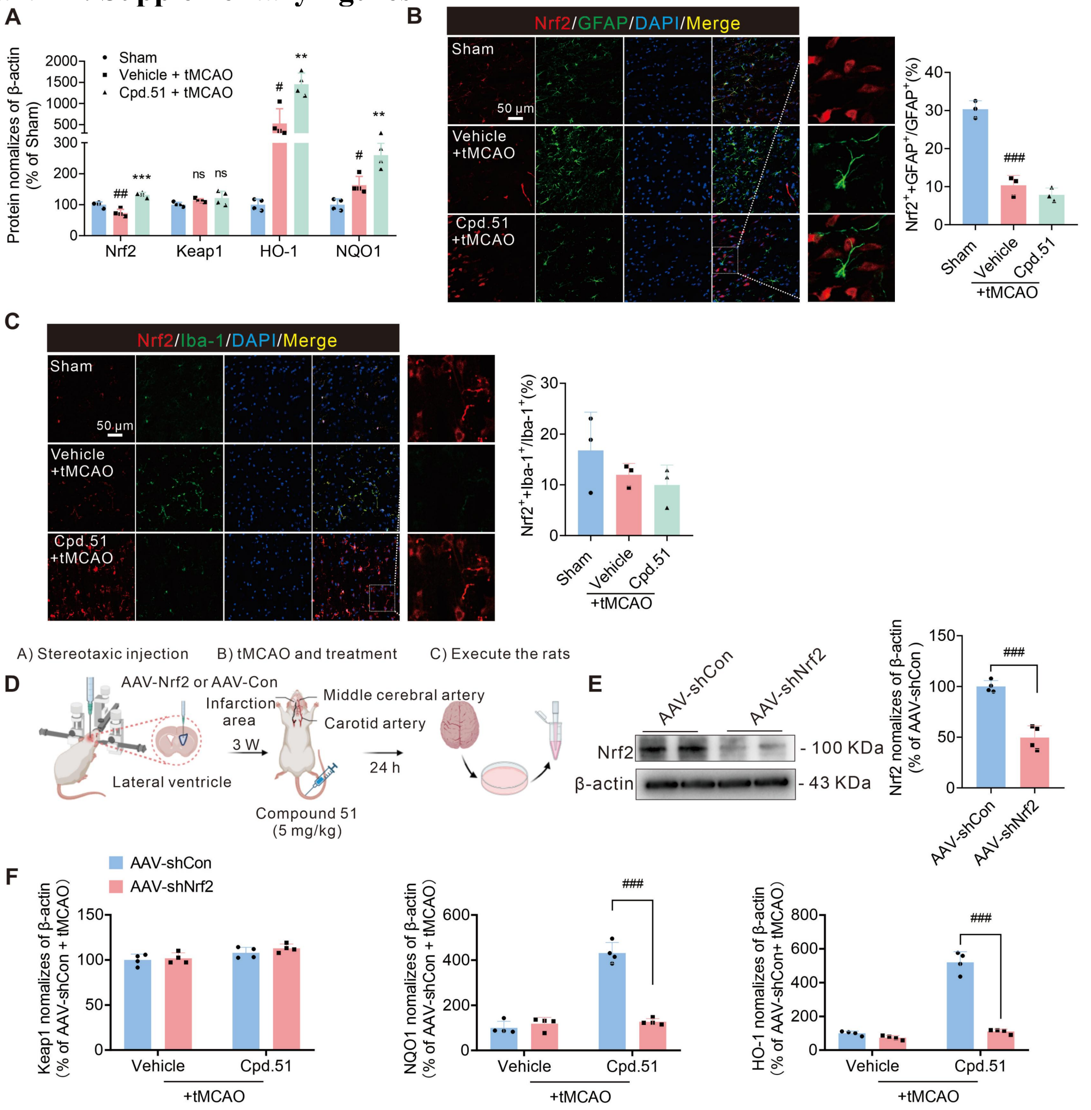

Figure S1. **Cpd.51 exerted an anti-AIS effect by promoting the activation of Nrf2 in neurons.**
(A) Cpd.51 promoted the expression of Nrf2, HO-1, and NQO1 in the cortex surrounding the infarct area. *n* = 4. (B–C) Representative immunofluorescence images and quantification of astrocyte or microglia stained with Nrf2. And quantitative analysis of Nrf2$^+$ GFAP$^+$ or Nrf2$^+$ Iba-1$^+$ cells in the peri-infarct region of rats at 24 h after ischemia. *n* = 3. (D) Schematic diagram of Nrf2-knockdown process *in vivo* models. (E) Western blotting bands of Nrf2-knockdown in the cortex surrounding the infarct area of rats and the quantitative analysis of it. *n* = 4. (F) Western blotting images quantification of Keap1, HO-1 and NQO1 in the cortex surrounding the infarct area of rats. *n* = 4. Results are expressed as mean ± SD. A–C, $^{\#}P$<0.05, $^{\#\#}P$<0.01, $^{\#\#\#}P$<0.001 vs. Sham group. $^{**}P$<0.01, $^{***}P$<0.001 vs. tMCAO group. Statistical differences among groups were analyzed by using One-way ANOVA followed by Tukey's post-hoc test. E and F, $^{\#\#\#}P$<0.001 vs. Cpd.51 plus tMCAO plus AAV-shCon group. E, Statistical differences among groups were analyzed by using Student's t test. F, Statistical differences among groups were analyzed by using Two-way ANOVA followed by Tukey's post-hoc test.

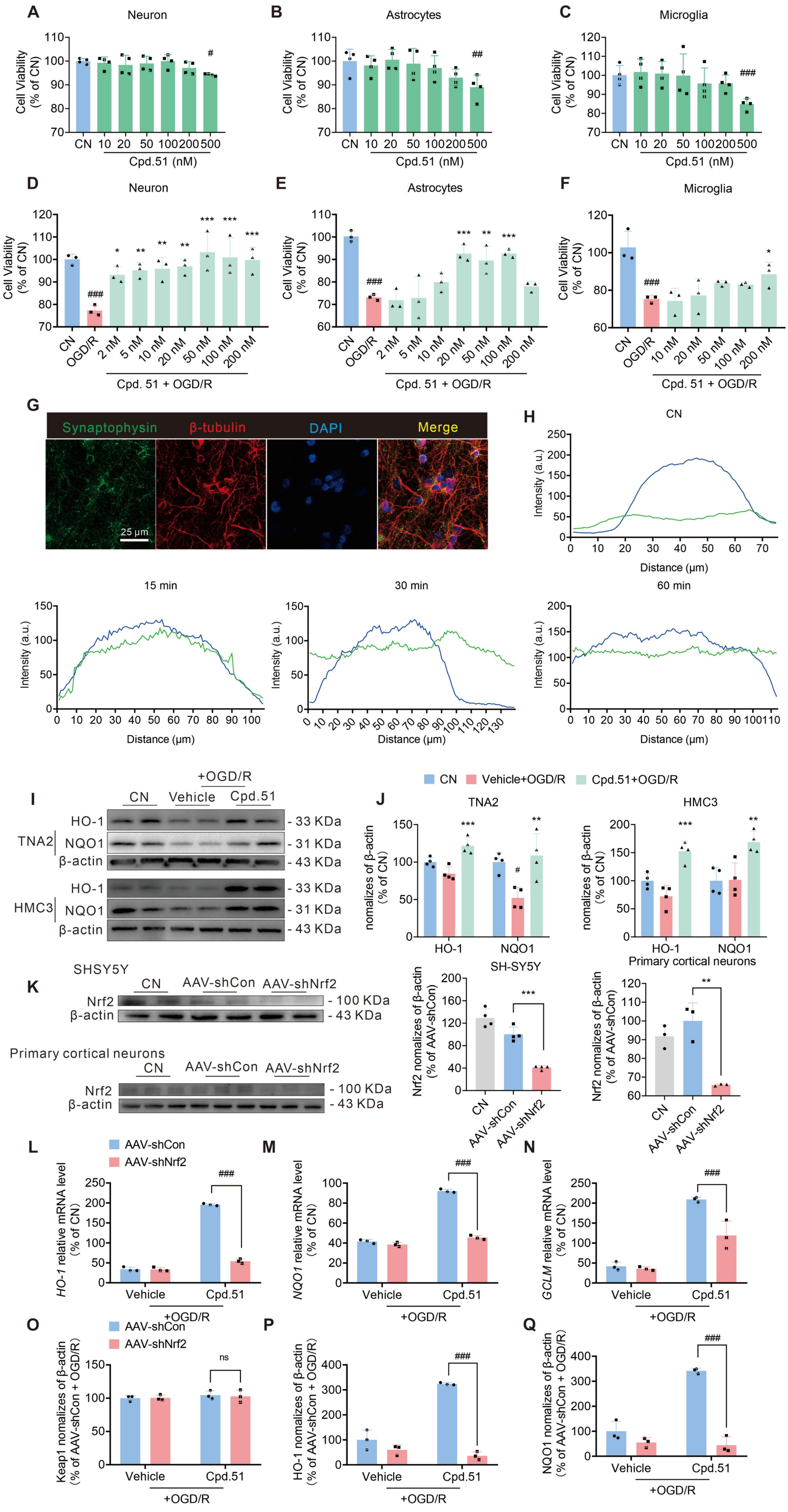
A
Neuron
Cell Viability (% of CN)
CN 10 20 50 100 200 500
Cpd.51 (nM)
B
Astrocytes
C
Microglia
D
Neuron
E
Astrocytes
F
Microglia
CN
OGD/R
2 nM
5 nM
10 nM
20 nM
50 nM
100 nM
200 nM
Cpd. 51 + OGD/R
G
Synaptophysin
β-tubulin
DAPI
Merge
25 μm
H
CN
15 min
30 min
60 min
Intensity (a.u.)
Distance (μm)
I
+OGD/R
CN
Vehicle
Cpd.51
TNA2
HMC3
HO-1
NQO1
β-actin
- 33 KDa
- 31 KDa
- 43 KDa
J
CN
Vehicle+OGD/R
Cpd.51+OGD/R
TNA2
HMC3
nomalizes of β-actin (% of CN)
Primary cortical neurons
K
SHSY5Y
CN
AAV-shCon
AAV-shNrf2
Nrf2
- 100 KDa
- 43 KDa
Primary cortical neurons
SH-SY5Y
Nrf2 nomalizes of β-actin (% of AAV-shCon)
L
AAV-shCon
AAV-shNrf2
HO-1 relative mRNA level (% of CN)
Vehicle
Cpd.51
+OGD/R
M
NQO1 relative mRNA level (% of CN)
N
GCLM relative mRNA level (% of CN)
O
Keap1 nomalizes of β-actin (% of AAV-shCon + OGD/R)
ns
P
HO-1 nomalizes of β-actin (% of AAV-shCon + OGD/R)
Q
NQO1 nomalizes of β-actin (% of AAV-shCon + OGD/R)

Figure S2. Cpd.51 exerted superior neuroprotective effects and promotes Nrf2 nuclear translocation.

(A–C) Cells were incubated with various concentrations of Cpd.51 for 24 h to investigate the cytotoxicity. $n = 4$. (D–F) Cpd.51 ameliorated damage to cells by OGD/R, as measured by CCK-8. $n = 3$. (G) Representative immunofluorescence images of Synaptophysin with β-tubulin about primary cortical neurons. (H) Treatment with Cpd.51 (100 nM) for 15 min promotes Nrf2 nuclear translocation. (I, J) Cpd.51 promotes the expression of Nrf2, HO-1, and NQO1 in TNA2 and HMC3 cells with OGD/R. $n = 4$. (K) Western blotting bands of Nrf2-knockdown in SH-SY5Y or primary cortical neurons and the quantitative analysis of it. $n = 4$ or 3. (L–N) The qRT-PCR analysis of *HO-1*, *NQO1* and *GCLM* gene expression in SH-SY5Y. $n = 3$. (O–Q) Western blotting images quantification of Keap1, HO-1, NQO1 in SH-SY5Y. $n = 3$. Results are expressed as mean ± SD. A–J, $^{\#}P<0.05$, $^{\#\#}P<0.01$, $^{\#\#\#}P<0.001$ vs. CN group. $^{*}P<0.05$, $^{**}P<0.01$, $^{***}P<0.001$ vs. OGD/R group. K–Q, $^{**}P<0.01$, $^{***}P<0.001$ vs. AAV-shCon group. $^{\#\#\#}P<0.001$ vs. Cpd.51 plus OGD/R plus AAV-shCon group. A–K, Statistical differences among groups were analyzed by using One-way ANOVA followed by Tukey's post-hoc test. L–Q, Statistical differences among groups were analyzed by using Two-way ANOVA followed by Tukey's post-hoc test.

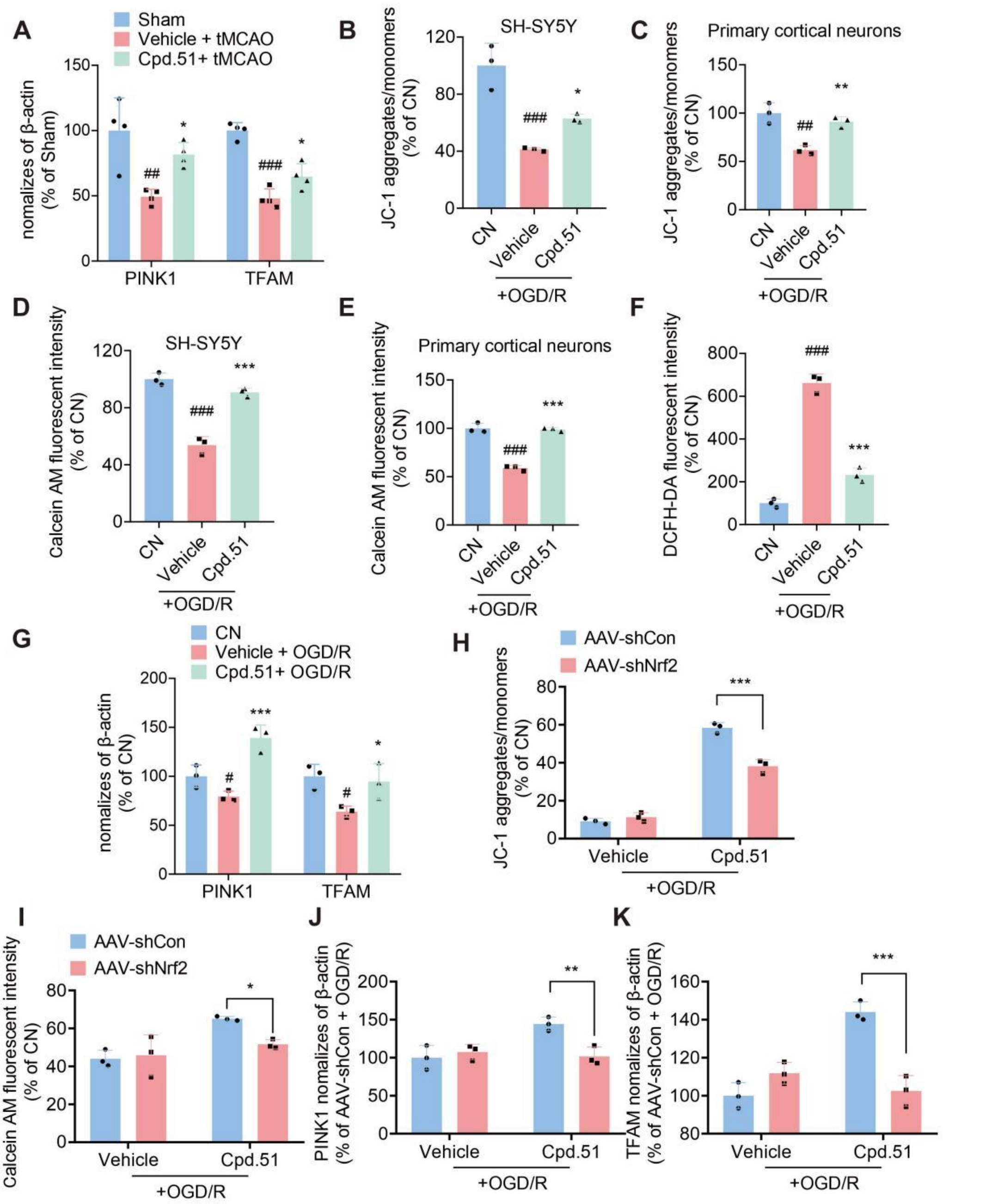

Figure S3. **Cpd.51 improved mitochondrial damage after ischemic stroke.**
(A) Cpd.51 reduced the protein expression of PINK1 and TFAM in the cortex surrounding the infarct area. *n* = 4. (B, C) Statistical analysis of JC-1 staining detected by immunofluorescence in SH-SY5Y cells or primary cortical neurons cells. *n* = 3. (D, E) Statistical analysis Calcein AM fluorescence intensity to test the state of the MPTP in SH-SY5Y cells or primary cortical neurons cells. *n* = 3. (F) By quantitatively analyzing the fluorescence intensity of DCFH-DA, evaluate the effect of Cpd.51 on ROS levels in SH-SY5Y cells following OGD/R-induced injury. *n* = 3. (G) Cpd.51 reduced the protein expression of PINK1 and TFAM in the SH-SY5Y cells with OGD/R. *n* = 3. (H, I) Knockdown of Nrf2 reversed the protective effect of Cpd.51 on mitochondria, as demonstrated by JC-1 assay for mitochondrial membrane potential and Calcein AM assay for MPTP. (J, K) Western blotting images quantification of PINK1 and TFAM in the cortex surrounding the infarct area of SD rats. *n* = 3. Results are expressed as mean ± SD. A–G, $^{\#}P<0.05$, $^{\#\#}P<0.01$, $^{\#\#\#}P<0.001$ vs. Sham group or CN group. $^{*}P<0.05$, $^{**}P<0.01$, $^{***}P<0.001$ vs. tMCAO group or OGD/R group. Statistical differences among groups were analyzed by

using One-way ANOVA followed by Tukey's post-hoc test. H–K, *P<0.05, **P<0.01, ***P<0.001 vs. Cpd.51 plus OGD/R plus AAV-shCon group. Statistical differences among groups were analyzed by using Two-way ANOVA followed by Tukey's post-hoc test.

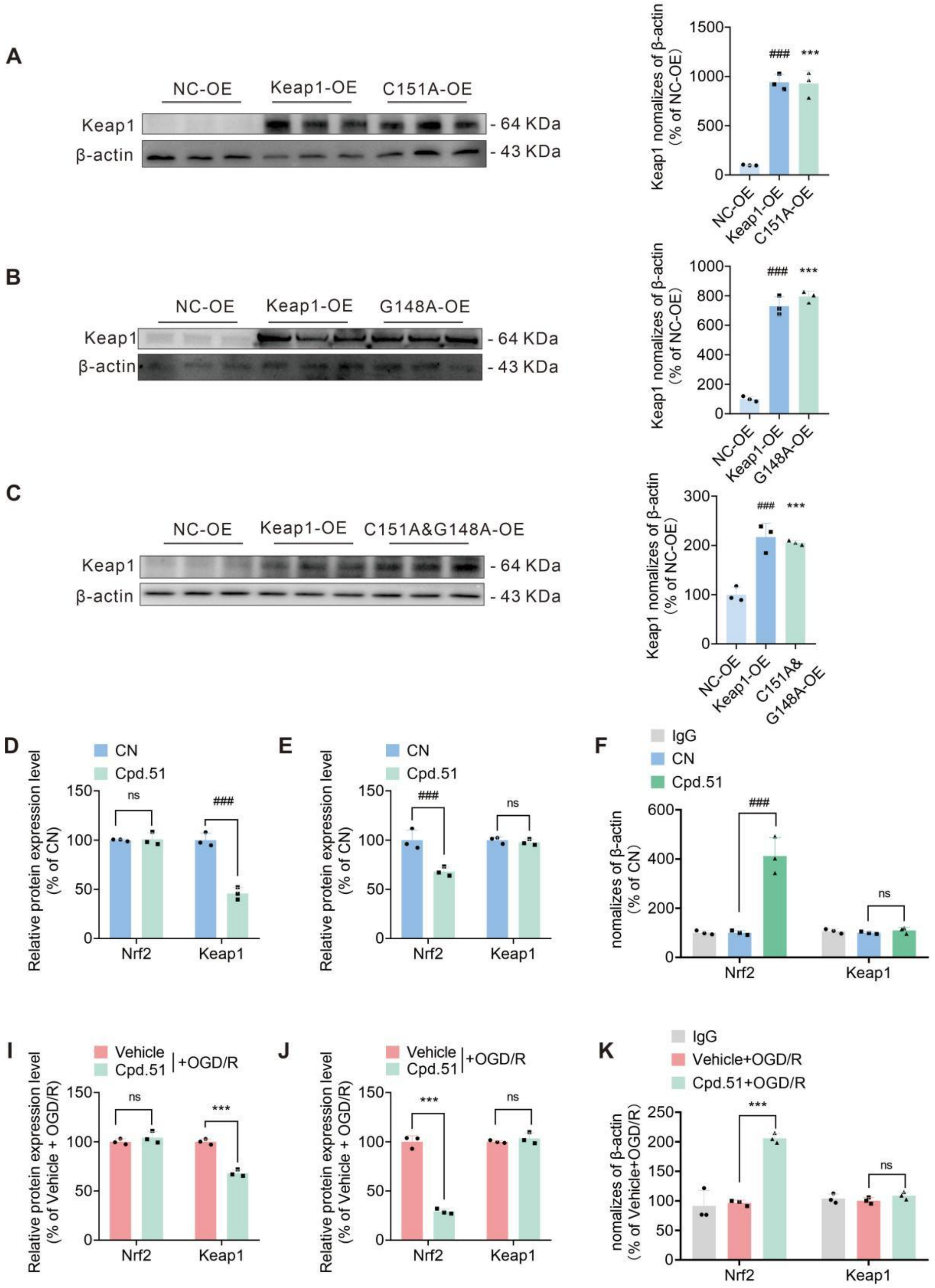

Figure S4. Cpd.51 exerted Nrf2-activating effects by binding to the Keap1 Gly148 and Cys151 positions.

(A–C) The Keap1 plasmid transfection increased protein expression of Keap1 in Hek-293T cells. n = 3. ###P<0.001 vs. NC-OE, ***P<0.001 vs. Keap1-OE. (D–K) Western blotting images and quantification were performed to detect the effect of Cpd.51 on the interaction between Nrf2 and Keap1 under physiological or OGD/R conditions. n = 3. ###P<0.001 vs. CN

group, $^{***}P$<0.001 vs. OGD/R group. All statistical differences among groups were analyzed by using One-way ANOVA followed by Tukey's post-hoc test.

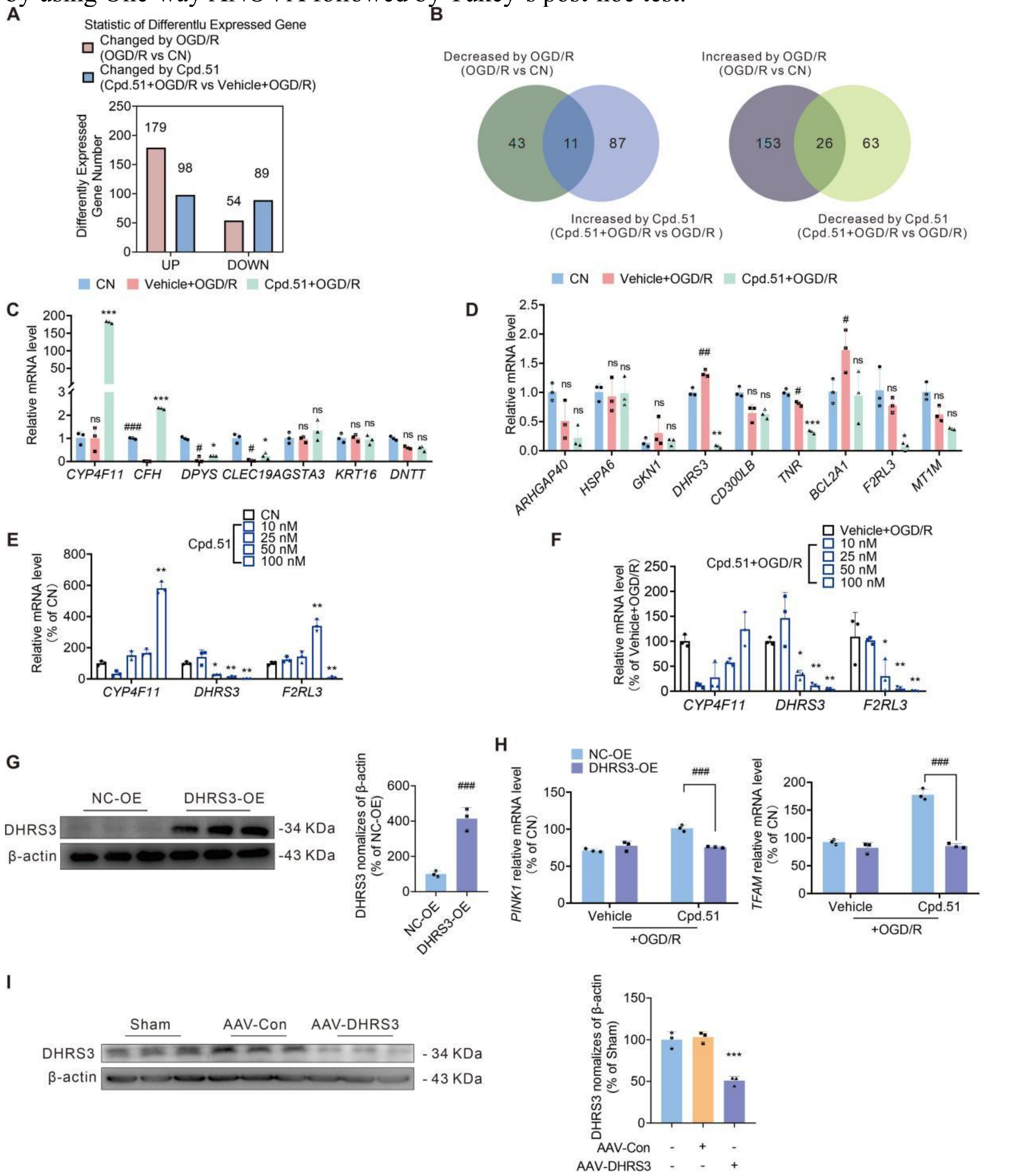

Figure S5. KEGG enrichment analysis of RNA sequencing data.

(A–B) The statistical results of RNA sequencing. (C–D) Select genes showing significant changes based on transcriptomic results for validation through qRT-PCR analysis. $n = 3$. (E–F) The qRT-PCR analysis of *CYP4F11*, *DHRS3* and *F2RL3* gene expression in SH-SY5Y cells. $n = 3$. (G) Western blotting bands of DHRS3-overexpression in SH-SY5Y and the quantitative analysis of it. $n = 3$. (H) mRNA Expression Assay for *PINK1* and *TFAM* genes. $n = 3$. (I) Western blotting bands of DHRS3-knockdown in the cortex surrounding the infarct area of rats and the quantitative analysis of it. $n = 3$. Results are expressed as mean ± SD. C–F, $^{\#}P$<0.05, $^{\#\#}P$<0.01, $^{\#\#\#}P$<0.001 vs. CN group. $^{*}P$<0.05, $^{**}P$<0.01, $^{***}P$<0.001 vs. OGD/R group. I, $^{***}P$<0.001 vs. AAV-Con group. Statistical differences among groups were analyzed by using One-way ANOVA followed by Tukey's post-hoc test. G, $^{\#\#\#}P$<0.001 vs. NC-OE group. Statistical differences among groups were analyzed by using Student's t test. H,

$^{###}P$<0.001, Statistical differences among groups were analyzed by using Two-way ANOVA followed by Tukey's post-hoc test.

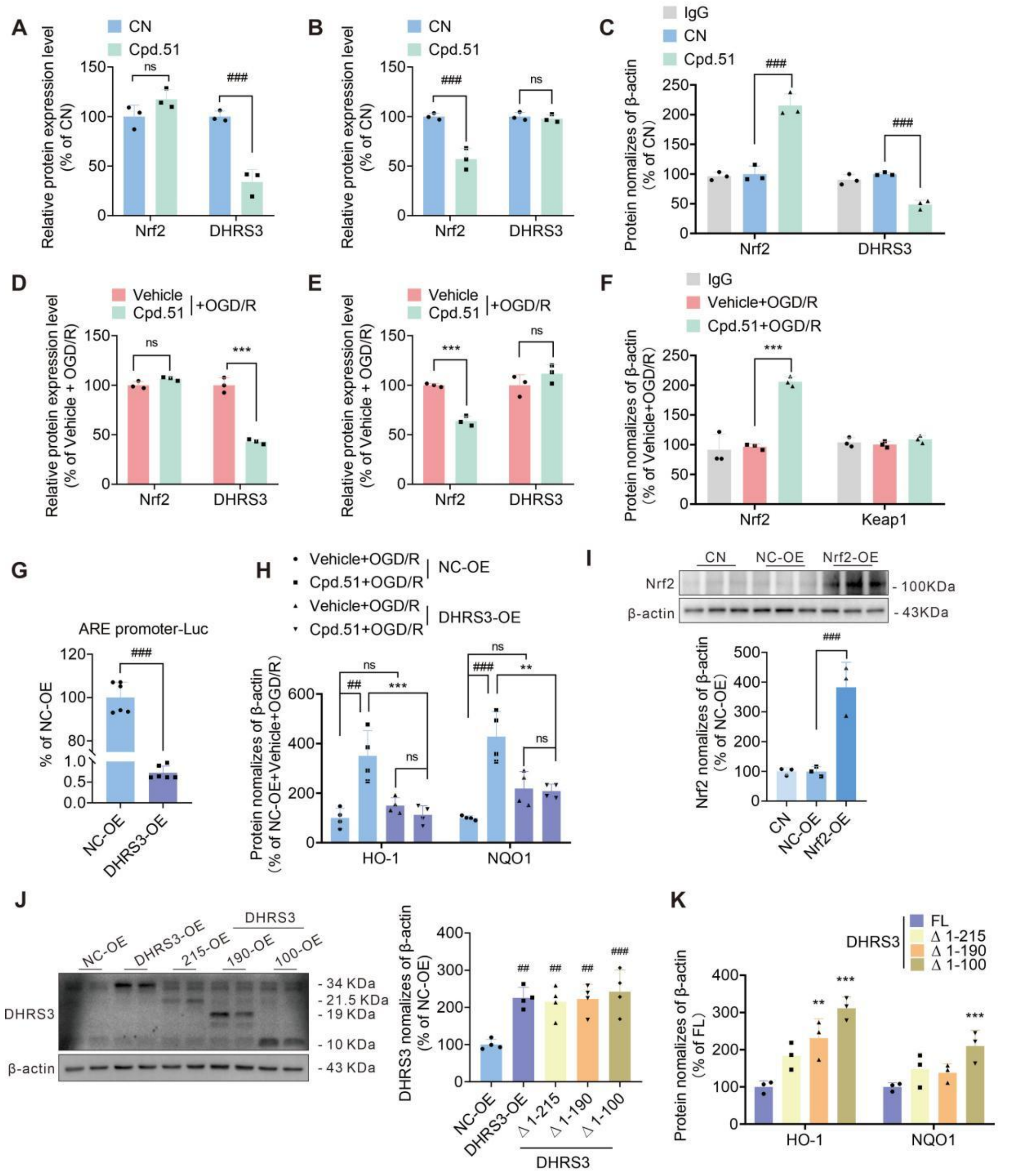

Figure S6. Cpd.51 inhibited the interaction between Nrf2 and DHRS3.
(A–C) Quantification of western blotting images was performed to detect the effect of Cpd.51 on the interaction between Nrf2 and DHRS3 under physiological conditions. n = 3. (D–F) Quantification of western blotting images was performed to detect the effect of Cpd.51 on the interaction between Nrf2 and DHRS3 under OGD/R condition. n = 3. (G) DHRS3-overexpression inhibited ARE luciferase reporter activity in Hek-293T cells under physiological conditions. n = 6. (H) Western blotting images quantification of HO-1 and NQO1 in SH-SY5Y cells. n = 4. (I) Western blotting bands of overexpress-Nrf2 in Hek-293T cells and the quantitative analysis of it. n = 3. (J) Overexpression - DHRS3 truncation form in Western blotting bands of Hek-293T cells and the quantitative analysis of it. n = 4. (K) Quantification of western blotting images in Figure 7K. n = 3. Results are expressed as mean ± SD. $^{##}P$<0.01, $^{###}P$<0.001. $^{**}P$<0.01, $^{***}P$<0.001. Statistical differences among groups were

analyzed by using One-way ANOVA followed by Tukey's post-hoc test. G, Statistical differences among groups were analyzed by using Student's t test.

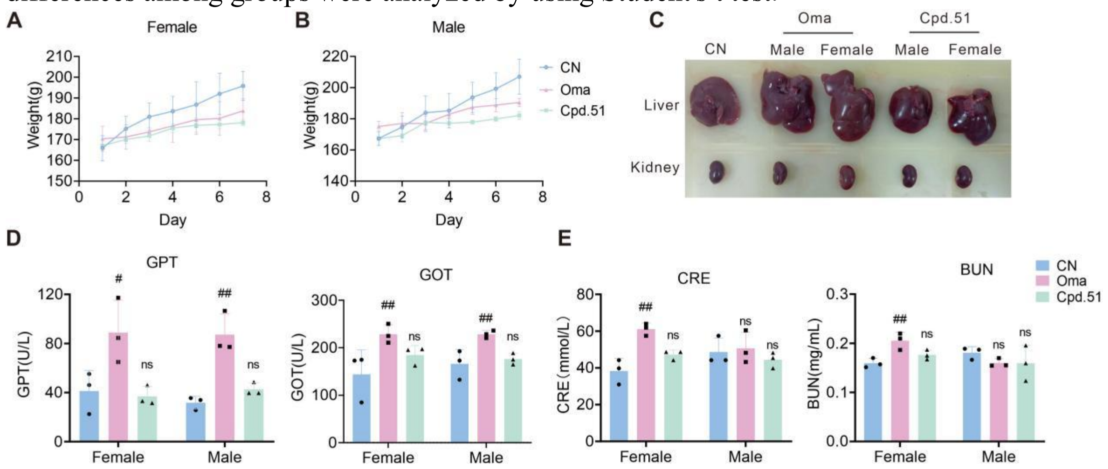

Figure S7. Comparison of acute toxicity between Cpd.51 and Oma.
(A, B) After administration (10 mg/kg), the body weight increased progressively in female and male SD rats. n = 3. (C) Representative liver and kidney morphology after 7 days of administration. (D, E) ELISA analysis of GPT, GOT, CRE and BUN in serum from female and male SD rats administrated with Cpd.51, Oma or Vehicle. n = 3. Results are expressed as mean ± SD; $^{\#}P<0.05$, $^{\#\#}P<0.01$ vs. CN group. Statistical differences among groups were analyzed by using One-way ANOVA followed by Tukey's post-hoc test.

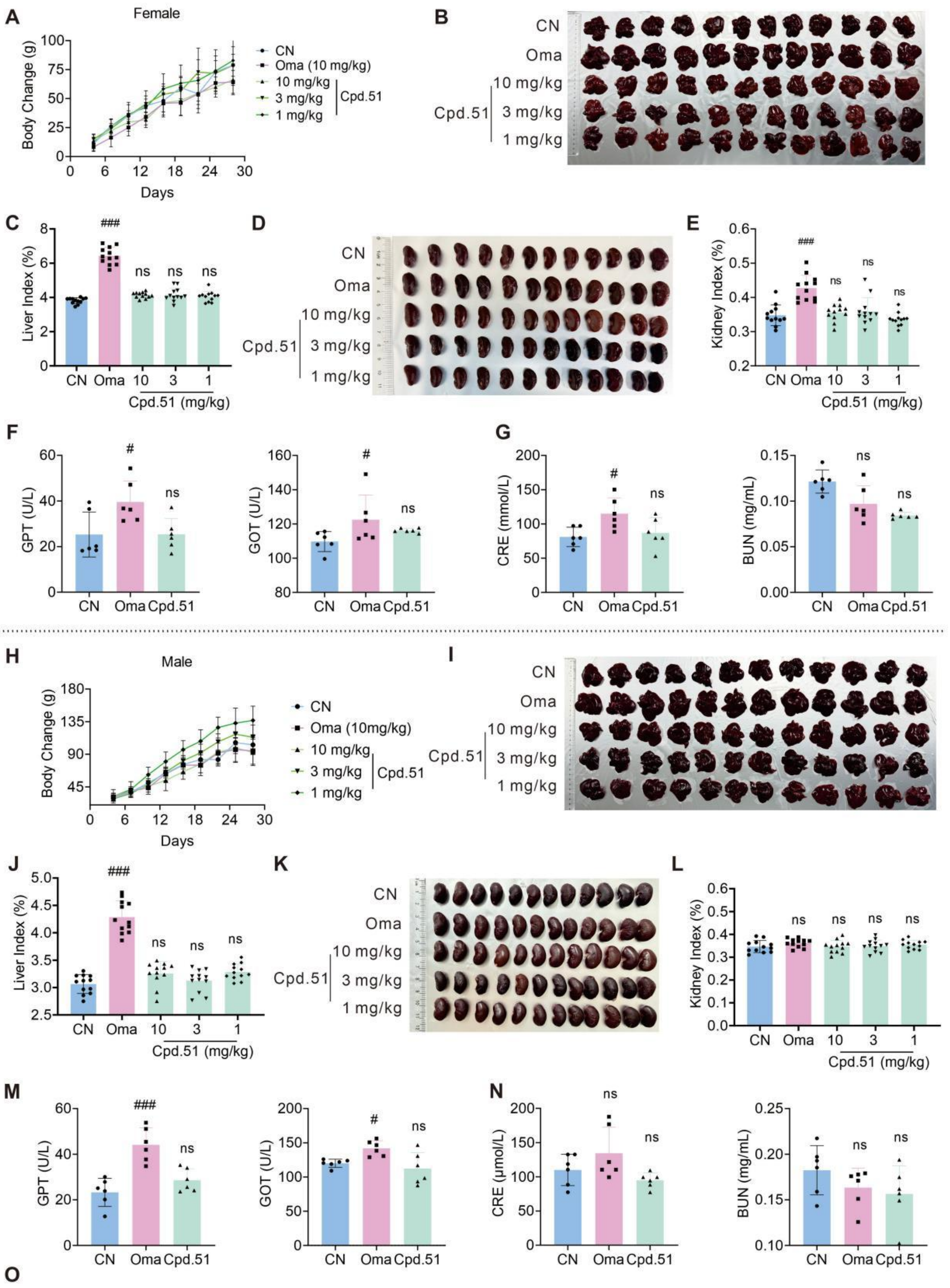

Group	Sex	Plasma (ng/mL)	Brain (ng/g)	Brain/ Plasma	Liver (μg/g)	Liver/ Plasma	Kidney (μg/g)	Kidney/ Plasma
Cpd.51	Female	170.6 ± 42.7	54.5 ± 27.4	0.10 ± 0.04	26.0 ± 14.4	53.0 ± 32.7	5.1 ± 1.4	10.0 ± 2.5
	Male	77.8 ± 18.4	15.1 ± 2.5	0.07 ±0.02	10.1 ± 3.4	43.6 ± 9.9	2.9 ± 0.8	12.6 ± 2.3
Oma	Female	1585.7 ± 397.0	2149.1 ± 398.7	0.42 ± 0.09	88.0 ± 54.0	17.8 ± 12.0	34.0 ±5.4	6.9 ± 2.5
	Male	997.8 ± 211.6	692.6 ±85.2	0.25 ± 0.05	30.5 ±16.1	9.8 ± 3.9	26.5 ±2.5	9.1 ± 2.3

Figure S8. **Comparison of long-term toxicity between Cpd.51 and Oma.**
(A, H) After administration, the body weights of both female and male SD rats gradually increased. *n* = 12. (B, C, I, J) The liver condition and liver index of rats 28 days after administration. *n* = 12. (D, E, K, L) The state of the kidneys in female/male rats 28 days after

administration. n = 12. (F, G, M, N) ELISA analysis of GPT, GOT, CRE and BUN in serum from female and male SD rats administrated with Cpd.51 (10 mg/kg), Oma or Vehicle. n = 12. (O) Detection of tissue distribution of Cpd.51 and Oma. n = 5. Results are expressed as mean ± SD; $^{\#}P<0.05$, $^{\#\#\#}P<0.001$ vs. CN group. Statistical differences among groups were analyzed by using One-way ANOVA followed by Tukey's post-hoc test.
