# Supplementary material for: Discovery of a novel Nrf2 activator that modulates mitochondrial function in neurons by regulating DHRS3-Nrf2 interaction after ischemic stroke
Source: Theranostics. 2026 Mar 30;16(10):5713–40. doi: 10.7150/thno.128602 (PMC13081163; doi:10.7150/thno.128602)

Compound ID: 51

EW53902-30-P1A CDCl3 ZKNJ_018_400MHz

药明康德 WuXi AppTec

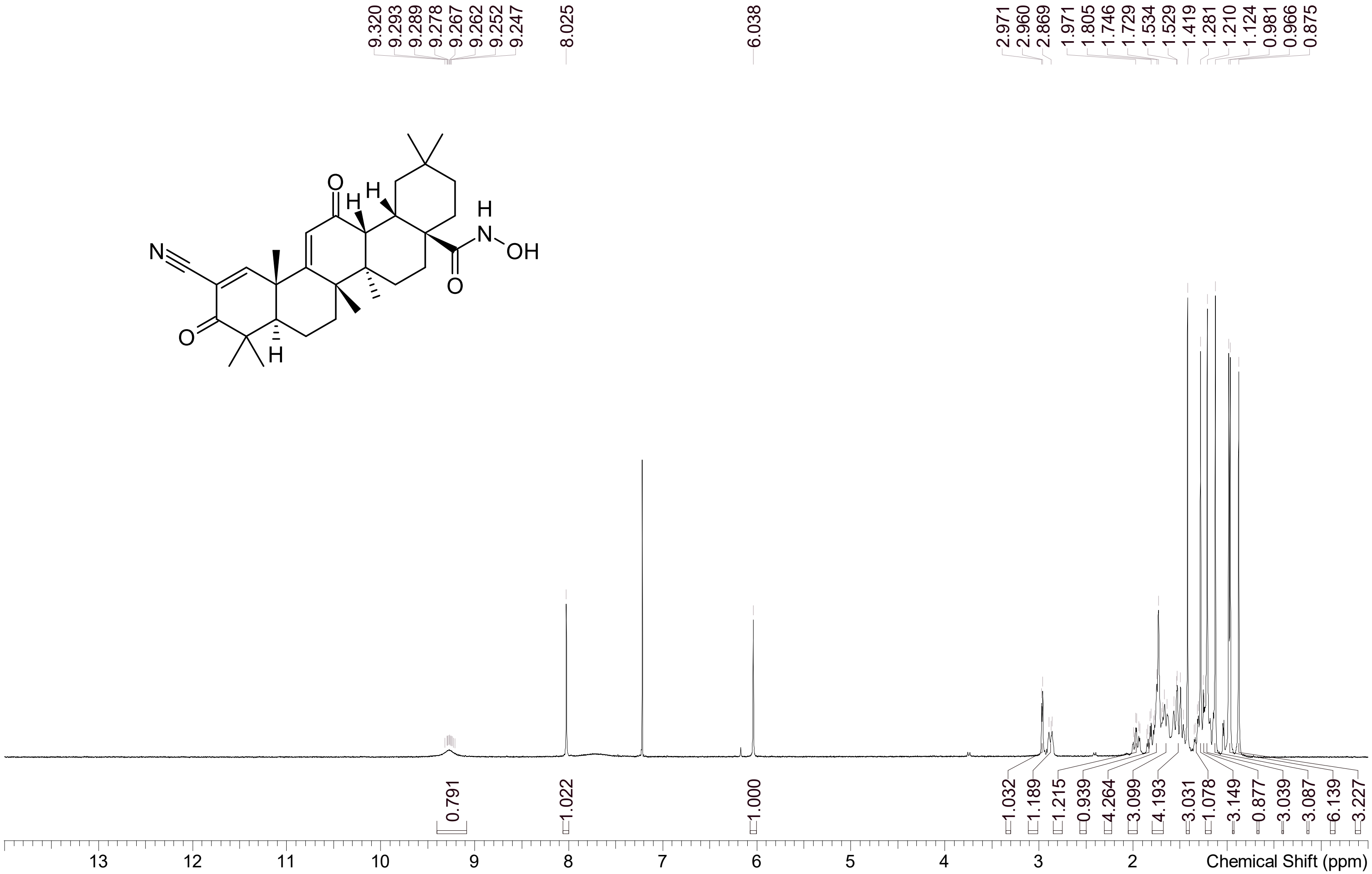

Acquisition Time (sec)	3.0002
Comment	EW53902-30-P1A CDCl3 ZKNJ_018_400MHz
Date	04 Mar 2024 03:00:42 (GMT)
Frequency (MHz)	399.9340
Nucleus	1H
Number of Transients	8
Origin	QUANTUM-I
Original Points Count	24038
Owner	admin
Points Count	65536
Pulse Sequence	s1pul30
Receiver Gain	70.28
SW(cyclical) (Hz)	8012.00
Solvent	CHLOROFORM-d
Spectrum Offset (Hz)	2399.6038
Spectrum Type	undefined
Sweep Width (Hz)	8011.88
Temperature (degree C)	25.550

Confidential. For research information only

Operator:

Date:

Confidential. For research information only

LCMS REPORT

Compound ID : 51
Sample ID : EW53902-30-P1B
Injection Vol : 3ul
Location : vial34
Tray Name :2
Acq Method : D:\method\5-95AB_3min_220&254.lcm
Org DataFile : D:\DATA\2024\2403\240304\EW53902-30-P1B.lcd
Injection Date : 3/4/2024 11:26:47
Instrument :LCMS-070

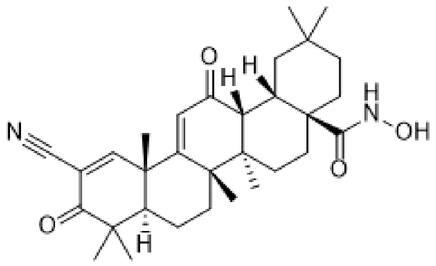

Exact Mass: 506.31

Chromatogram

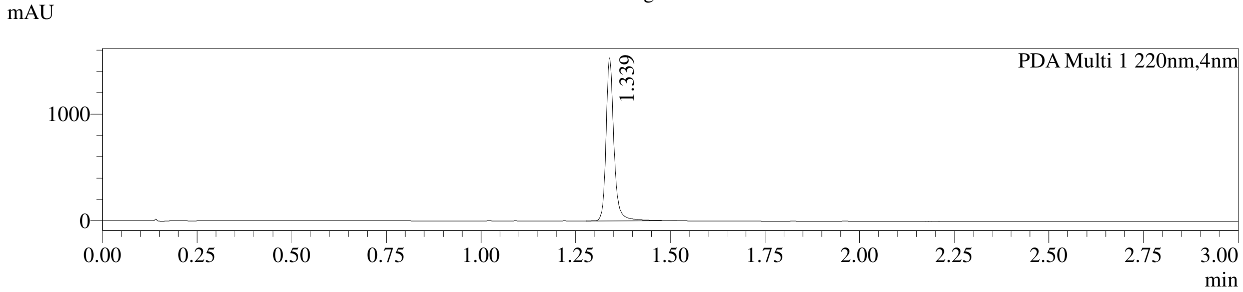

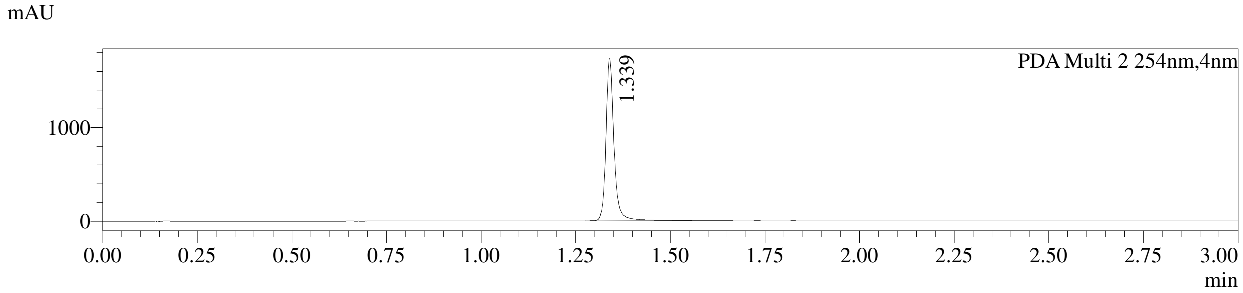

1 PDA Multi 1 / 220nm,4nm
2 PDA Multi 2 / 254nm,4nm

MS Chromatogram

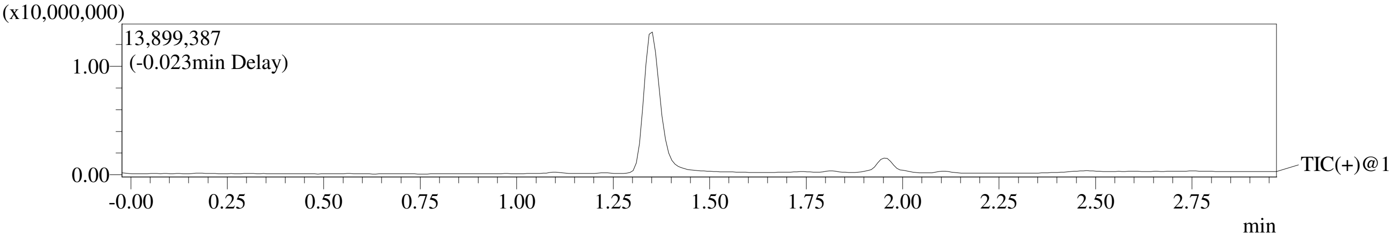

Integration Result

Peak Table

PDA Ch1 220nm

Peak#	Ret. Time	Height	Height%	USP Width	Area	Area%
1	1.339	1529746	100.000	0.037	2235521	100.000

Peak Table

PDA Ch2 254nm

Peak#	Ret. Time	Height	Height%	USP Width	Area	Area%
1	1.339	1739577	100.000	0.038	2582084	100.000

Confidential. For research information only

Operator:______________

Date:_________________

Confidential. For research information only

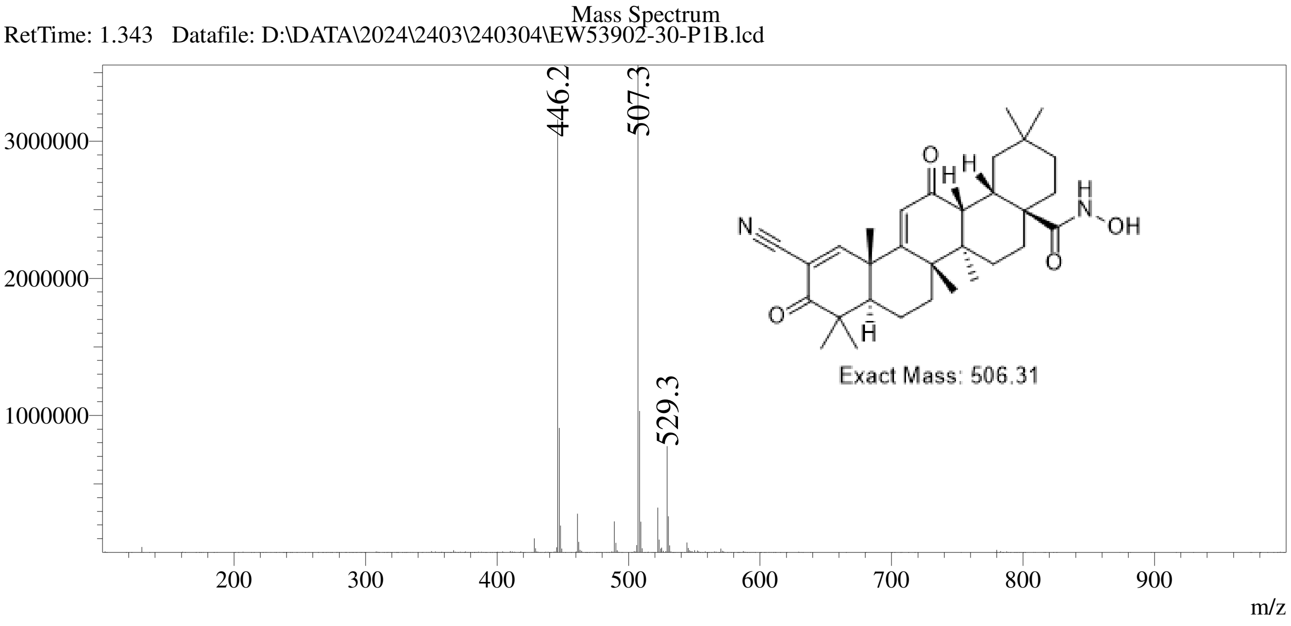

Confidential. For research information only

HPLC REPORT

Compound ID : 51
Sample ID : EW53902-30-P1D
Vial# : 27
Injection Volumn : 3
Filename : D:\DATA\2024\2403\240304\EW53902-30-P1D.lcd
Method Name : D:\method\10-80AB_3min.lcm
Instrument : HPLC-087
Run time : 03/04/2024 10:22:13

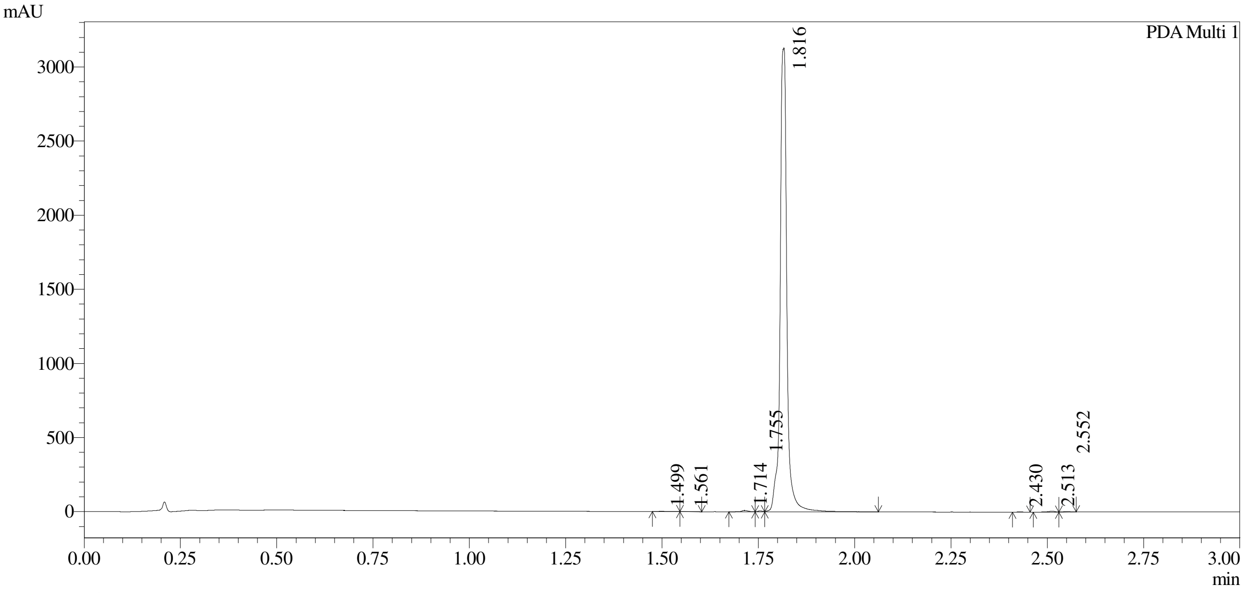

1 PDA Multi 1 / 220nm,4nm

Integration result

PeakTable

PDA Ch1 220nm

Peak#	Ret. Time	USP Width	Resolution	Height	Area	Area %
1	1.499	0.026	0.000	2529	3106	0.080
2	1.561	0.024	2.467	1311	1267	0.033
3	1.714	0.029	5.822	9116	11295	0.292
4	1.755	0.027	1.435	5943	5716	0.148
5	1.816	0.026	2.300	3127796	3832109	99.062
6	2.430	0.023	25.097	1555	1496	0.039
7	2.513	0.030	3.126	6536	8508	0.220
8	2.552	0.031	1.297	3982	4895	0.127
Total				3158768	3868394	100.000

Operater : ________________

Date : ________________

Confidential. For research information only

HPLC REPORT

Compound ID : 51
Sample ID : EW53902-30-P1D
Filename : D:\DATA\2024\2403\240304\EW53902-30-P1D.lcd
Method Name : D:\method\10-80AB_3min.lcm
Instrument : HPLC-087
Run time : 03/04/2024 10:22:13

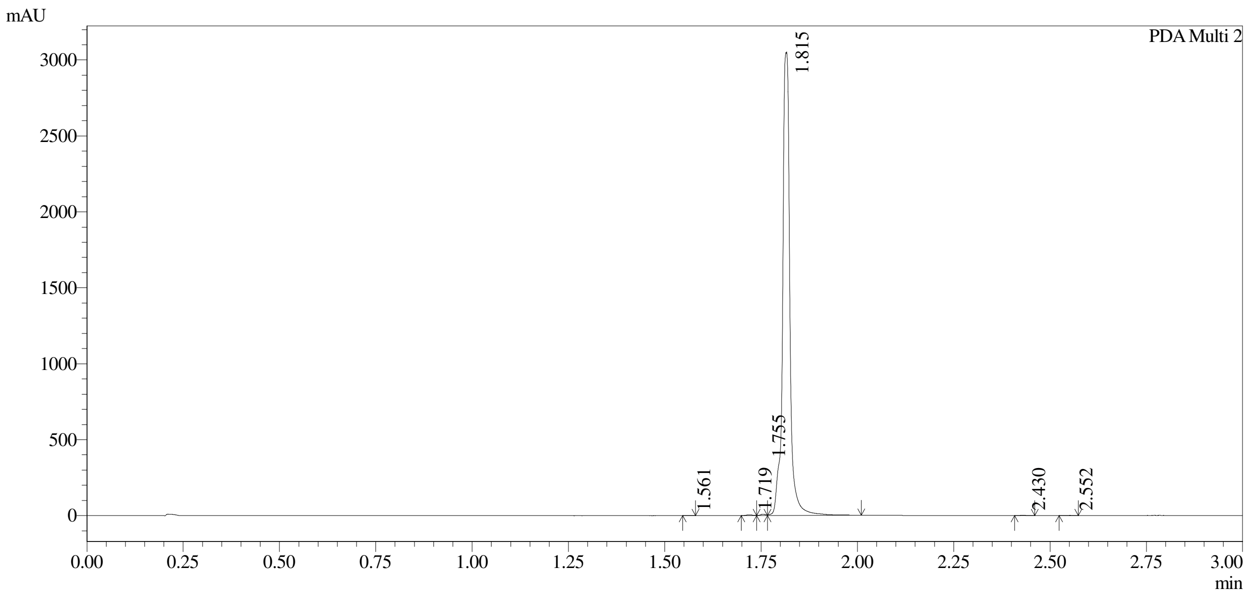

1 PDA Multi 2 / 254nm,4nm

Integration result

PeakTable

PDA Ch2 254nm

Peak#	Ret. Time	USP Width	Resolution	Height	Area	Area %
1	1.561	0.023	0.000	1421	1272	0.031
2	1.719	0.032	5.749	5338	6424	0.159
3	1.755	0.027	1.219	9355	9014	0.223
4	1.815	0.027	2.249	3048181	4029562	99.508
5	2.430	0.024	24.239	2005	1940	0.048
6	2.552	0.025	5.031	1138	1267	0.031
Total				3067439	4049480	100.000

Operater : ________________

Date : ________________

Confidential. For research information only

HPLC REPORT

Compound ID : 51
Sample ID : EW53902-30-P1D
Filename : D:\DATA\2024\2403\240304\EW53902-30-P1D.lcd
Method Name : D:\method\10-80AB_3min.lcm
Instrument &Column : HPLC-087
Run time : 03/04/2024 10:22:13

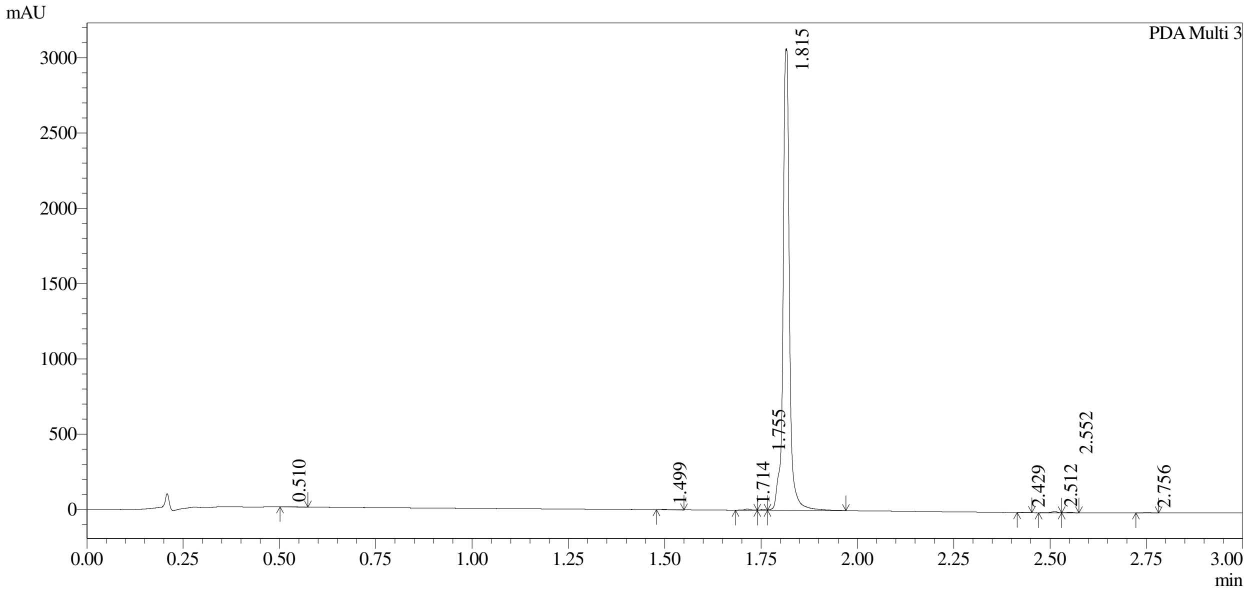

1 PDA Multi 3 / 215nm,4nm

Integration result

PeakTable

PDA Ch3 215nm

Peak#	Ret. Time	USP Width	Resolution	Height	Area	Area %
1	0.510	0.055	0.000	357	1060	0.029
2	1.499	0.026	24.421	2401	2935	0.081
3	1.714	0.028	7.981	7700	8845	0.245
4	1.755	0.025	1.537	5764	5304	0.147
5	1.815	0.025	2.408	3059282	3570172	99.006
6	2.429	0.021	26.900	1191	1064	0.030
7	2.512	0.029	3.337	7963	9930	0.275
8	2.552	0.034	1.268	4535	5338	0.148
9	2.756	0.033	6.067	957	1365	0.038
Total				3090150	3606013	100.000

Operater : ________________

Date : ________________

Chiral SFC Report

Compound ID : 51
Sample ID : EW53902-30-P1A_I1601
Injection Vol : 5ul
Location :Tray1 vial5
Acq Method : D:\METHODS\IC-3-IPA+ACN(DEA)-20-60-3ML-35T.lcm
Raw Data : D:\DATA\2024\202403\20240304\EW53902-30-P1A_I1601.lcd
Injection Date : 3/4/2024 4:54:14 PM
Instrument : CAS-WH-ANA-SFC-I(SHIMADZU LC-30ADsf)

Method details: "Column:Chiralpak IC-3 50*4.6mm I.D.,3um
Mobile phase:Phase A for CO2,and Phase B for IPA+ACN(0.05%DEA);
Gradient elution: From 20% to 60% of IPA+ACN(0.05%DEA) in CO2 ,
Flow rate:3mL/min;Detector:PDA;
Column Temp:35C;Back Pressure:100Bar"

Chromatogram

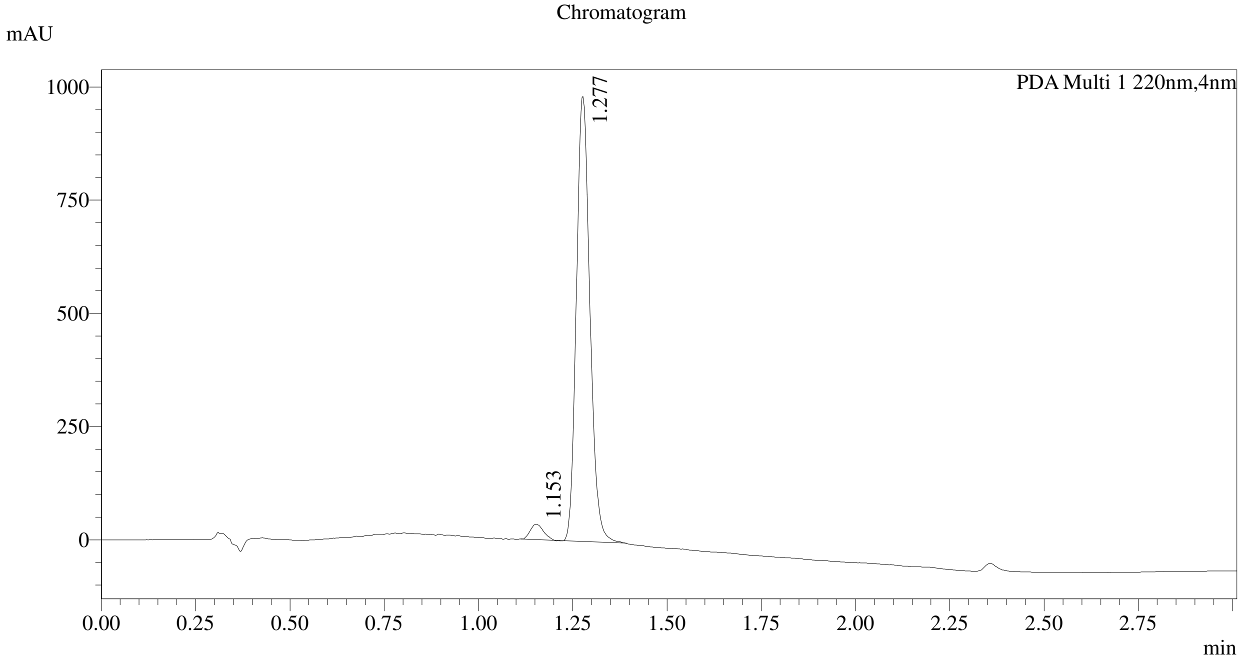

1 PDA Multi 1 / 220nm,4nm

Integration Result

Peak Table

PDA Ch1 220nm

Peak#	Ret. Time	Height	Height%	Resolution(USP)	Area	Area%
1	1.153	33036	3.279	--	83232	3.273
2	1.277	974431	96.721	1.797	2459655	96.727

Operator:______________

Date:_________________

Confidential. For research information only

LCMS REPORT

Compound ID : 1
Sample ID : EW53902-29-P1A
Injection Vol : 3ul
Location : vial02
Tray Name :2
Acq Method : D:\method\5-95CD_1.5min_220&254_POS.lcm
Org DataFile : D:\DATA\2024\2402\240229\EW53902-29-P1A.lcd
Injection Date : 02/29/2024 12:13:39
Instrument : LCMS-053 2-109

Chromatogram

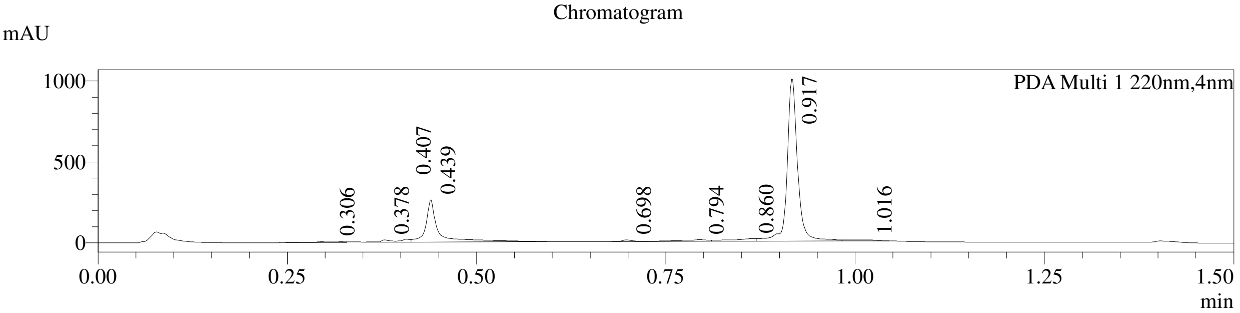

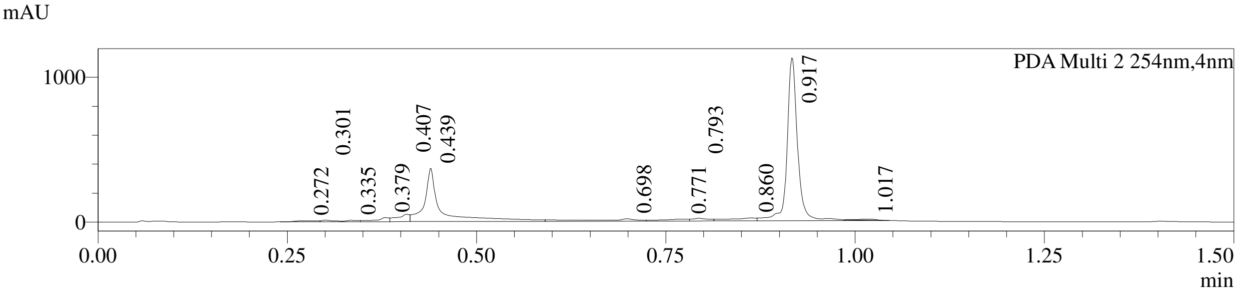

1 PDA Multi 1 / 220nm,4nm
2 PDA Multi 2 / 254nm,4nm

MS Chromatogram

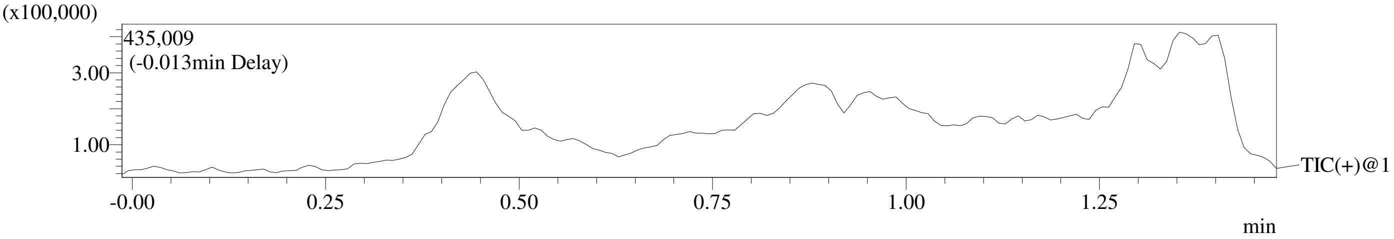

Integration Result

Peak Table

PDA Ch1 220nm

Peak#	Ret. Time	Height	Height%	USP Width	Area	Area%
1	0.306	8241	0.613	0.051	18296	1.299
2	0.378	14750	1.097	0.024	14364	1.020
3	0.407	17695	1.316	0.059	15138	1.075
4	0.439	259802	19.317	0.022	298568	21.196
5	0.698	9760	0.726	0.026	9412	0.668
6	0.794	10436	0.776	0.060	28023	1.989
7	0.860	15102	1.123	0.000	36080	2.561
8	0.917	1001985	74.500	0.025	969333	68.814
9	1.016	7169	0.533	0.191	19422	1.379

Peak Table

Confidential. For research information only

PDA Ch2 254nm

Peak#	Ret. Time	Height	Height%	USP Width	Area	Area%
1	0.272	7560	0.453	0.151	15931	0.809
2	0.301	10610	0.635	0.043	13142	0.668
3	0.335	9811	0.587	0.052	11046	0.561
4	0.379	27971	1.675	0.095	33984	1.726
5	0.407	49609	2.970	0.127	57820	2.937
6	0.439	365682	21.894	0.024	542425	27.553
7	0.698	16636	0.996	0.039	66536	3.380
8	0.771	12440	0.745	0.786	32800	1.666
9	0.793	18686	1.119	0.063	28184	1.432
10	0.860	19409	1.162	0.684	51490	2.616
11	0.917	1124282	67.312	0.025	1103405	56.049
12	1.017	7557	0.452	0.044	11887	0.604

Operator:______________

Date:__________________

Confidential. For research information only

Mass Spectrum

RetTime: 0.303 Datafile: D:\DATA\2024\2402\240229\EW53902-29-P1A.lcd

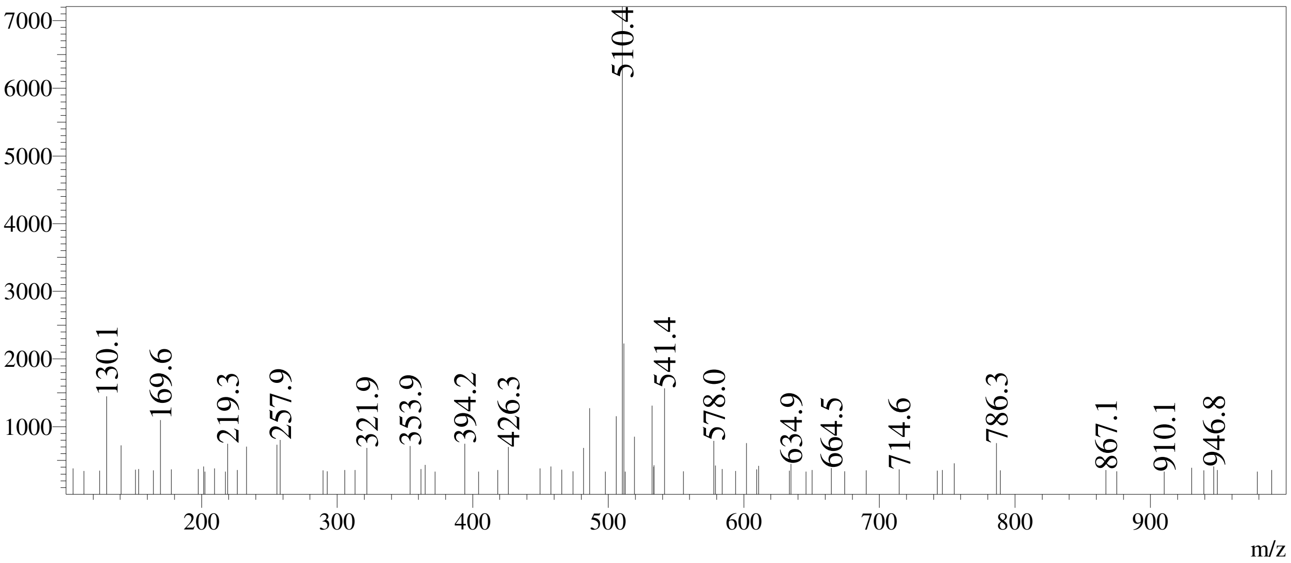

RetTime: 0.437 Datafile: D:\DATA\2024\2402\240229\EW53902-29-P1A.lcd

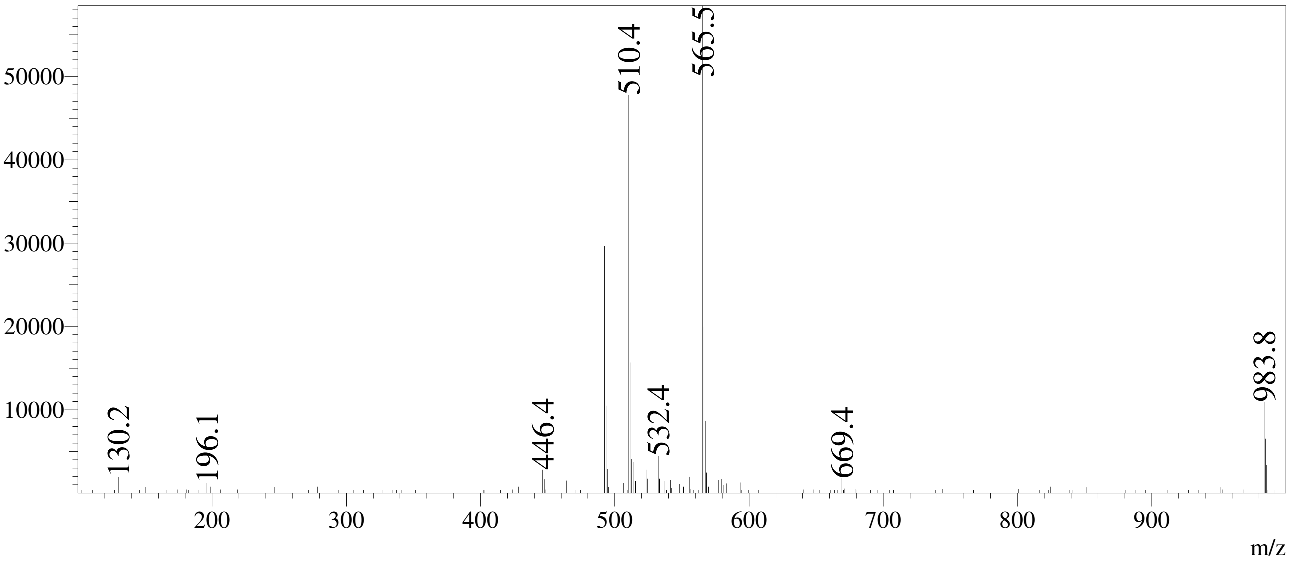

RetTime: 0.795 Datafile: D:\DATA\2024\2402\240229\EW53902-29-P1A.lcd

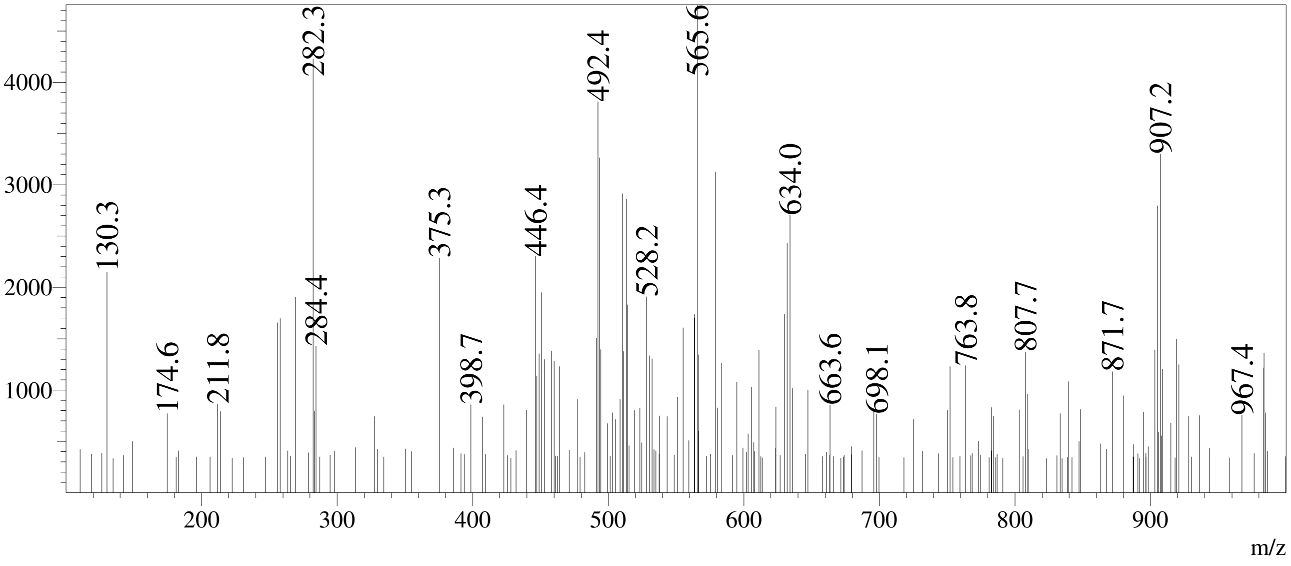

Confidential. For research information only

RetTime: 0.862 Datafile: D:\DATA\2024\2402\240229\EW53902-29-P1A.lcd

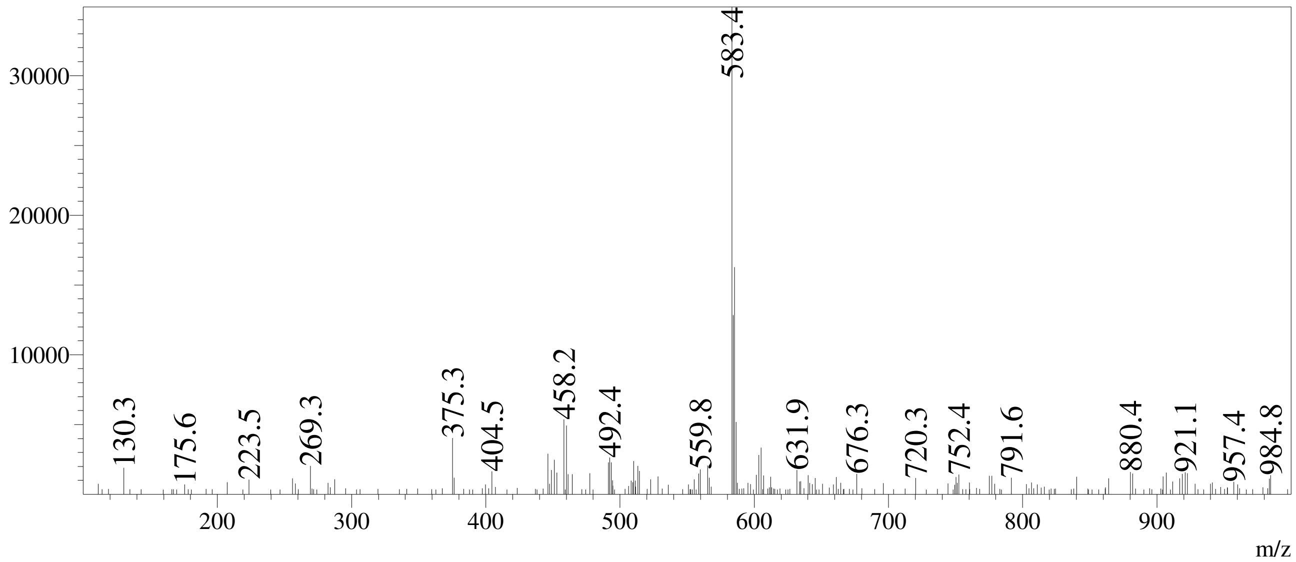

RetTime: 0.920 Datafile: D:\DATA\2024\2402\240229\EW53902-29-P1A.lcd

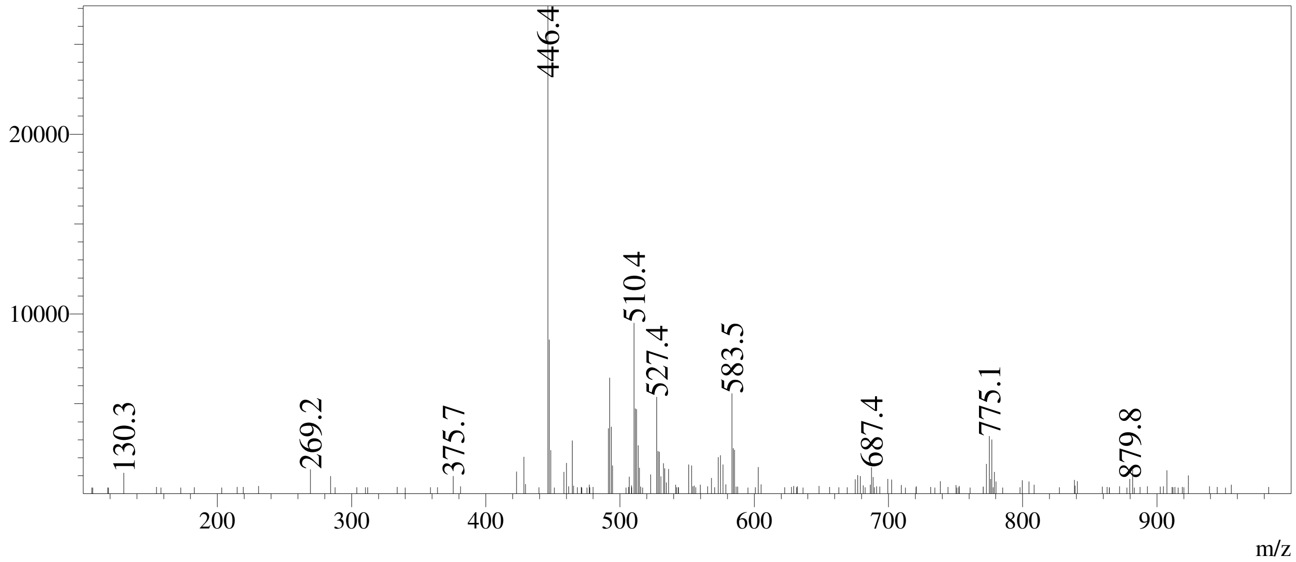

RetTime: 1.020 Datafile: D:\DATA\2024\2402\240229\EW53902-29-P1A.lcd

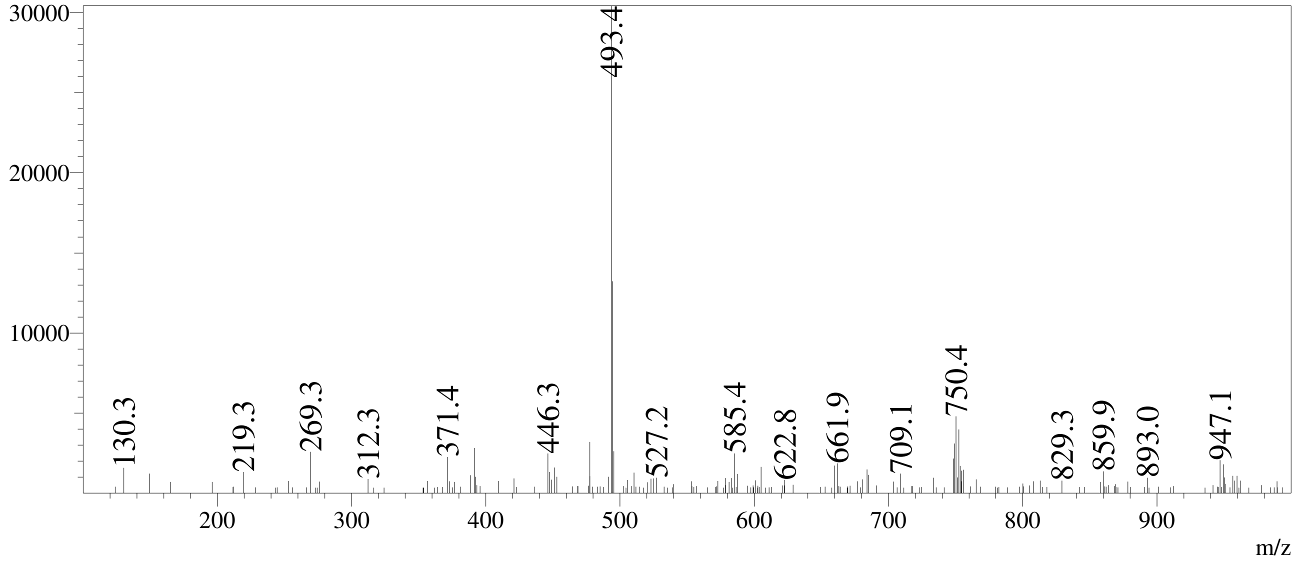

Metabolite profiling in human liver microsomes for Cpd.51

**Test article**	Cpd.51
**Purpose**	To identify major metabolites of test compound in human liver microsomes
**Study director(s)**	Jinyu Ba
**Major contributor(s)**	Haihong Li
**Effective date**	December 24th, 2025
**Sponsor**	烟台大学
**Study number**	CPB-P25-66382

Corresponding address

Shanghai ChemPartner Co., Ltd.

2727 Jinke Rd, Block A, Pudong, Shanghai, 201203, China

To the best of our knowledge this report has been reviewed for completeness, accuracy, and compliance with the protocol.

Study director:

____

Date

Jinyu Ba, M.S.
Study Director of Metabolite ID
Shanghai ChemPartner Co., Ltd.
Tel: (86) 21-51320088 Ext. 3073
Email: jyba@chempartner.com

Reviewed and approved by:

____

Date

Haihong Li, M.S.
Senior Research Scientist of Metabolite ID
Shanghai ChemPartner Co., Ltd.
Tel: (86) 21-51320088 Ext. 3074
Email: hhli1@chempartner.com

CONTENTS

1. INTRODUCTION AND OBJECTIVE 4
2. MATERIALS AND REAGENTS 4
3. EXPERIMENTAL PROCEDURES 4
4. LC-UV-MS CONDITIONS 5
5. SUMMARY 5

   Figure 1. LC-UV (λ = 250-260 nm) chromatograms of Cpd.51 and its metabolites incubated in human liver microsomes with or without NADPH for 0 min and 5 min 6
   Figure 2. LC-MS EIC of Cpd.51 and its metabolites incubated in human liver microsomes with NADPH for 0 min and 5 min. 6
   Figure 3. LC-MS EIC of M508 in T_0, T_5, $T_{5\text{-w/o}}$ and neat solution. 9
   Figure 4. Proposed metabolic pathways of Cpd.51 incubated in human liver microsomes in the presence of NADPH 10
   Table 1. Major metabolites of Cpd.51 incubated in human liver microsomes in the presence of NADPH 11
6. RESULTS AND DISCUSSION 12

INTRODUCTION AND OBJECTIVE

The objective of this study is to identify major metabolites of Cpd.51 generated in human liver microsomes.

In vitro metabolites identification was conducted after incubating Cpd.51 (parent compound, final concentration 10 μM) with human liver microsomes (final protein concentration, 1 mg/mL) at 37 °C in 100 mM potassium phosphate buffer containing 5 mM $MgCl_2$ in the presence of NADPH or without NADPH. The samples taken at 0 minute (min) and 5 min were quenched by methanol and analyzed using Waters G2-XS Q-Tof UPLC-MS system with positive-ion and negative-ion electrospray ionization. LC-MS extract ion chromatograms (EIC) of the T_5 and T_0 were compared to identify the major putative metabolites. The MS/MS spectra of Cpd.51 and the metabolites were obtained during positive-ion and negative-ion electrospray. The possible chemical structures of the metabolites were deduced based on their MS/MS spectra and retention times. The metabolic pathways of Cpd.51 in human liver microsomes were proposed.

MATERIALS AND REAGENTS

Cpd.51 (Purity: 99.1%), provided by 烟台大学
Acetonitrile, SIGMA-ALDRICH (Cat. # 34851-4L, Lot. # WXBD3947V)
Methanol (MeOH), SIGMA-ALDRICH (Cat. # 34860-4L-R, Lot. # WXBD3647V)
$K_2HPO_4 \cdot 3H_2O$, Titan (Lot. # P2128180)
KH_2PO_4, Titan (Lot. # P1343518)
$MgCl_2 \cdot 6H_2O$, Titan (Lot. # P1040534)
Human liver microsomes (HLM), XENOTECH (Cat. # H0610, Lot. # 2110263)
NADPH, MCE (Cat. # HY-F0003, Lot. # 728189)

EXPERIMENTAL PROCEDURES

1. Assay buffer: 100 mM potassium phosphate buffer (K^+/Mg^{2+} buffer, pH 7.4):

Reagent	$K_2HPO_4 \cdot 3H_2O$ (g)	KH_2PO_4 (g)	$MgCl_2 \cdot 6H_2O$ (g)	H_2O (mL)
	FW: 228.22	FW: 136.09	FW: 203.3	
100 mM	9.240265	1.297376	0.509460	500

2. Preparation of cofactor solution in the K^+/Mg^{2+} Buffer:

Reagent	K^+/Mg^{2+} buffer (mL)	NADPH (mg)	Total (mL)
8 mM NADPH	1.049	7.00	1.049

3. Assay procedure:

   1) T_0: add 20 μL of 20 mg/mL liver microsomes (LM) stock solution, 100 μL of cofactor solution and 279 μL of K^+/Mg^{2+} buffer to the T_0 vial, and quench the mixed solution by adding 1200 μL of MeOH, vortex at 1000 rpm for 5 min, and then add 1 μL of 4 mM compound solution.

   2) T_5: add 20 μL of 20 mg/mL liver microsomes (LM) stock solution, 100 μL of cofactor solution and 279 μL of K^+/Mg^{2+} buffer to the T_5 vial, and then start the reaction by adding 1 μL of 4 mM compound solution. After 5 min incubation, quench the reaction by adding 1200 μL of MeOH and vortex at 1000 rpm for 5 min.

   3) $T_{5\text{-w/o}}$: add 20 μL of 20 mg/mL liver microsomes (LM) stock solution and 379 μL of K^+/Mg^{2+} buffer to the $T_{5\text{-w/o}}$ vial, and then start the reaction by adding 1 μL of 4 mM compound solution. After 5 min incubation, quench the reaction by adding 1200 μL of

MeOH and vortex at 1000 rpm for 5 min.

4) Protein precipitation: centrifuge quenched samples at 14000 rpm for 5 min.

5) 100 μL of the supernatant was diluted with 50 μL of water and the mixture was vortexed for 5 min, and centrifuged at 14000 rpm for 5 min. Then 10 μL of the supernatant was injected onto LC-UV-MS for analysis.

LC-UV-MS CONDITIONS

1) LC condition:

Column: Acquity UPLC®BEH C18 (2.1 × 50 mm, 1.7 μm);

Mobile Phase: A (H_2O with 0.1% formic acid);
B (ACN with 0.1% formic acid);
Gradient Program:

Time (min)	Flow Rate (μL/min)	A (%)	B (%)
0.00	400	98	2
0.34	400	98	2
2.00	400	80	20
7.00	400	30	70
11.00	400	5	95
12.00	400	5	95
12.10	400	98	2
13.00	400	98	2

2) MS condition:

UPLC-UV-G2-XS Q-TOF: MS^E Centroid ESI (+/-)

**Scan Mode:** MS^E Centroid
Source
Capillary (kV): 3.00 (+) / 2.50 (-)
Sampling Cone: 40
Source Offset: 80
Temperature (°C)
Source: 120
Desolvation: 350
Gas Flows (L/h)
Cone Gas: 50
Desolvation Gas: 600

SUMMARY

Cpd.51 was transformed into 10 major metabolites in this study, which were named as $M_{E.M.}$ according to the exact mass of metabolites and the retention time in the current LC condition.

LC-UV chromatograms of Cpd.51 and its metabolites incubated in human liver microsomes are shown in **Figure 1**, while the LC-MS chromatograms are shown in **Figure 2**. Proposed metabolic pathways of Cpd.51 in human liver microsomes are shown in **Figure 4**. A summary, including observed *m*/*z* value, retention time, relative UV abundance, and MS peak area of Cpd.51 and its metabolites in human liver microsomes is presented in **Table 1**.

M508 was also detected in T_0, $T_{5\text{-w/o}}$ and neat solution, while the LC-MS chromatograms are shown in **Figure 3.**

Figure 1. LC-UV (λ = 250-260 nm) chromatograms of Cpd.51 and its metabolites incubated in human liver microsomes with or without NADPH for 0 min and 5 min

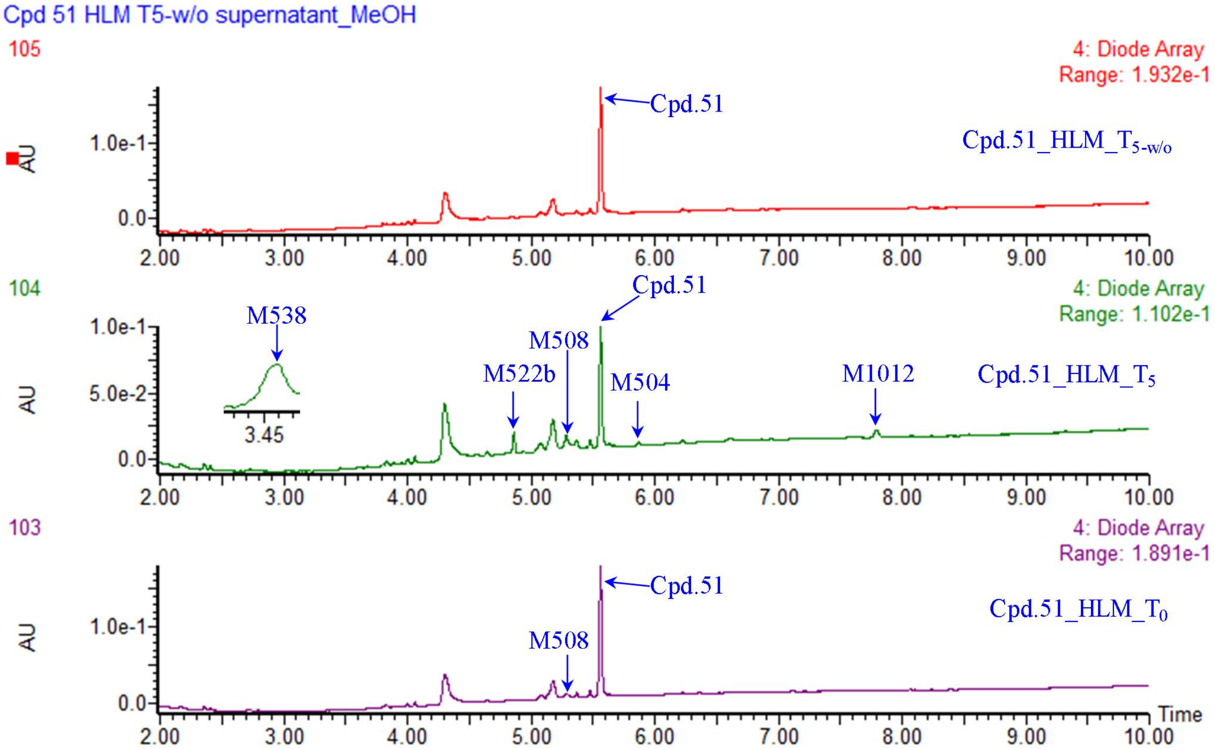

Figure 2. LC-MS EIC of Cpd.51 and its metabolites incubated in human liver microsomes with NADPH for 0 min and 5 min.

Positive-ion mode:

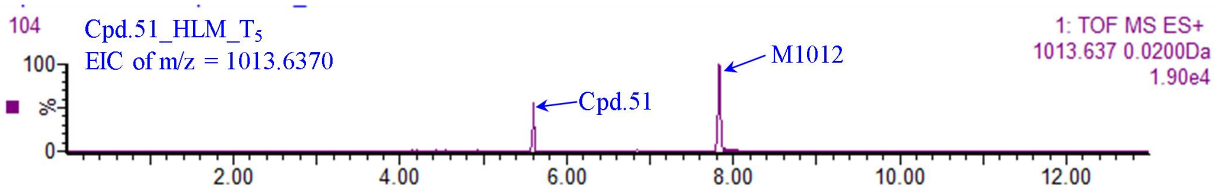

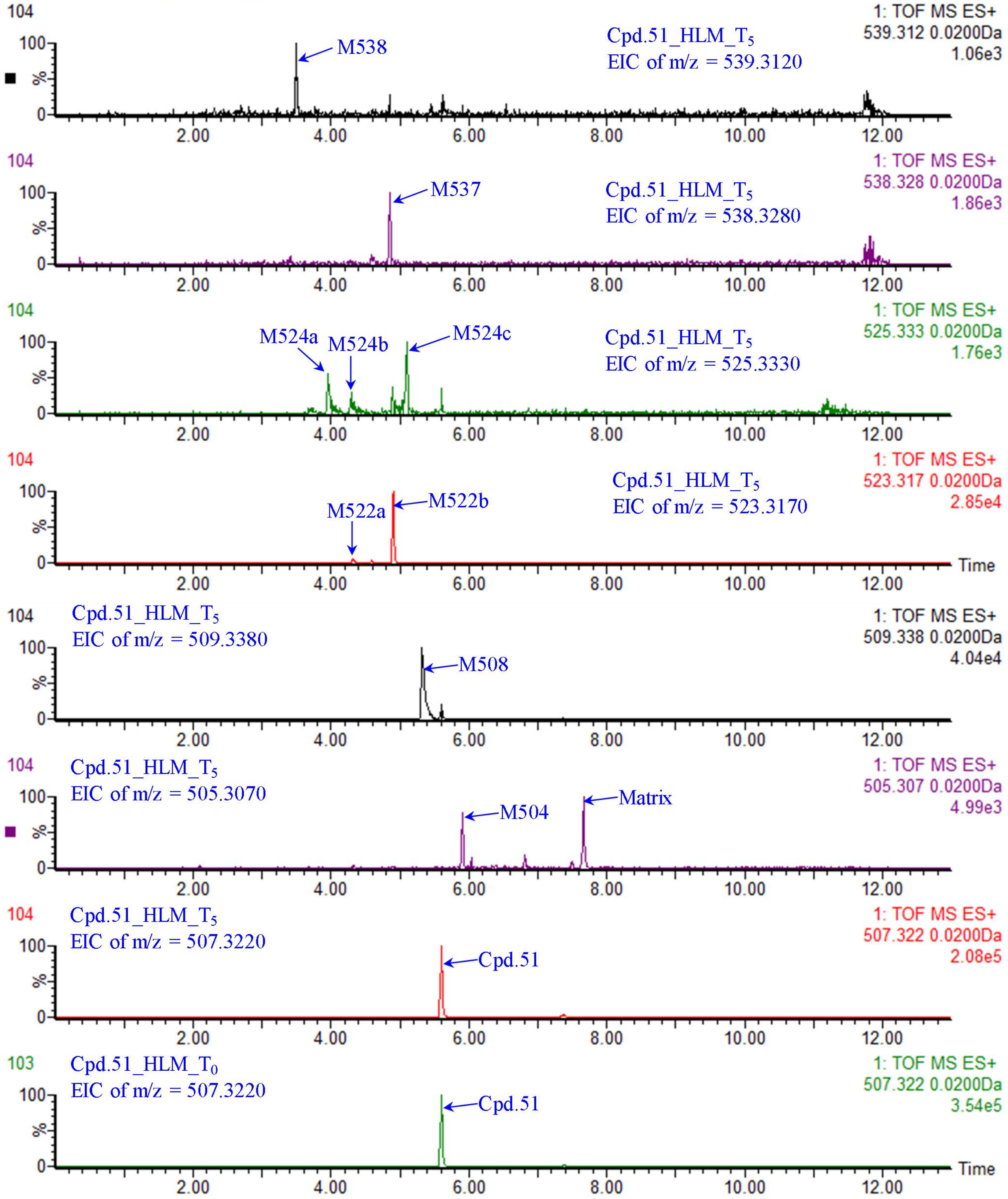

Negative-ion mode:

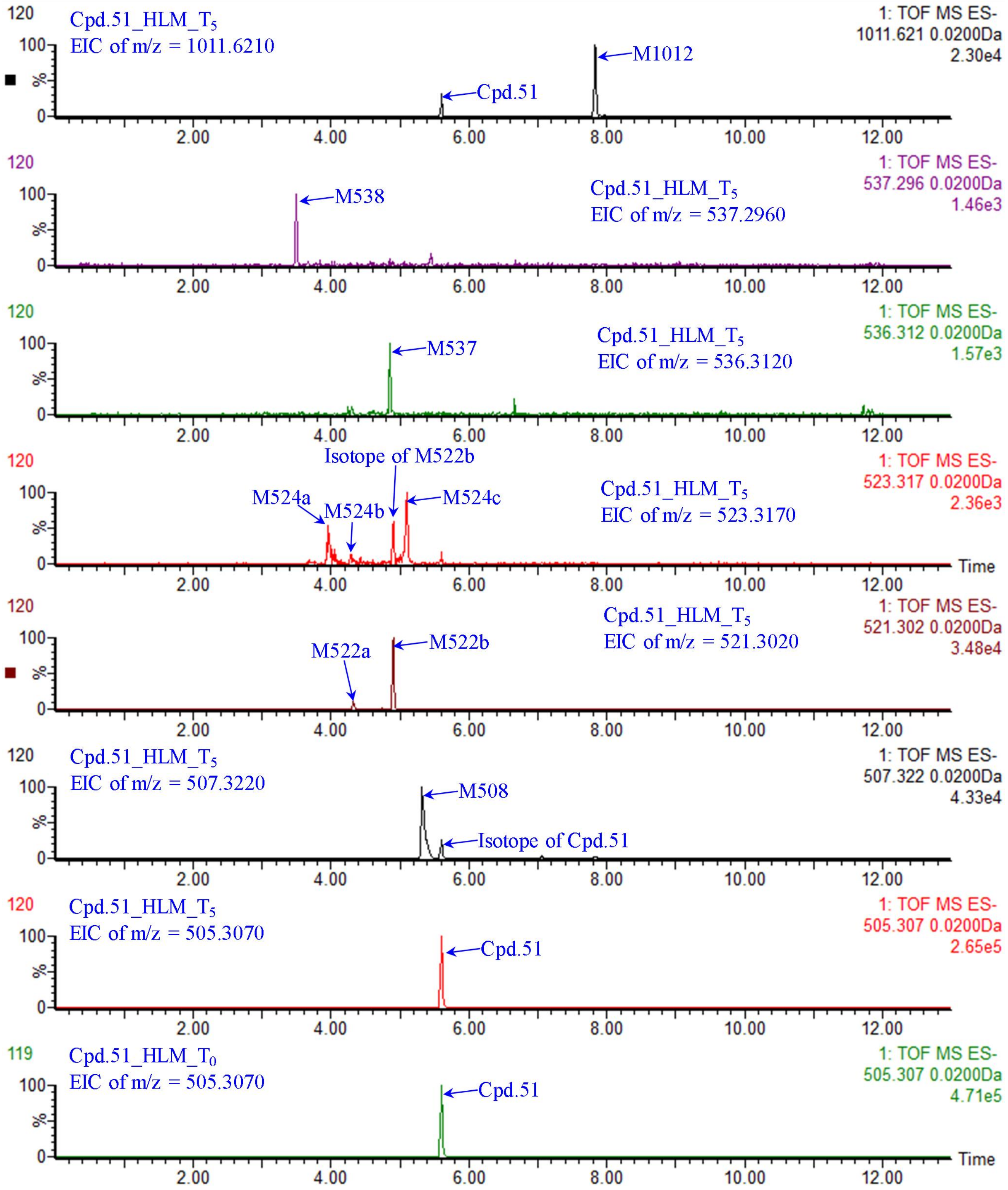
Cpd.51_HLM_T5
EIC of m/z = 1011.6210
1: TOF MS ES-
1011.621 0.0200Da
2.30e4
M1012
Cpd.51
Cpd.51_HLM_T5
EIC of m/z = 537.2960
537.296 0.0200Da
1.46e3
M538
Cpd.51_HLM_T5
EIC of m/z = 536.3120
536.312 0.0200Da
1.57e3
M537
Cpd.51_HLM_T5
EIC of m/z = 523.3170
523.317 0.0200Da
2.36e3
Isotope of M522b
M524a
M524b
M524c
Cpd.51_HLM_T5
EIC of m/z = 521.3020
521.302 0.0200Da
3.48e4
M522a
M522b
Cpd.51_HLM_T5
EIC of m/z = 507.3220
507.322 0.0200Da
4.33e4
M508
Isotope of Cpd.51
Cpd.51_HLM_T5
EIC of m/z = 505.3070
505.307 0.0200Da
2.65e5
Cpd.51
Cpd.51_HLM_T0
EIC of m/z = 505.3070
505.307 0.0200Da
4.71e5
Cpd.51
Time
%

Figure 3. LC-MS EIC of M508 in T_0, T_5, $T_{5\text{-w/o}}$ and neat solution.

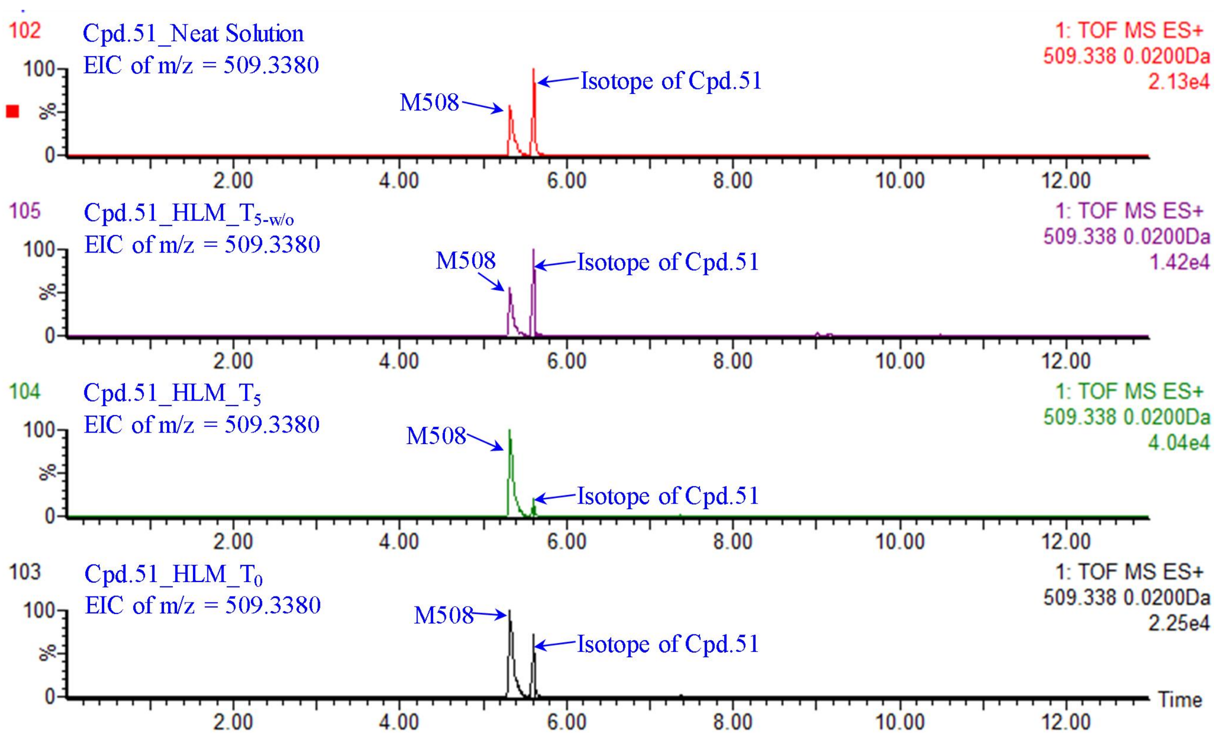

Figure 4. Proposed metabolic pathways of Cpd.51 incubated in human liver microsomes in the presence of NADPH

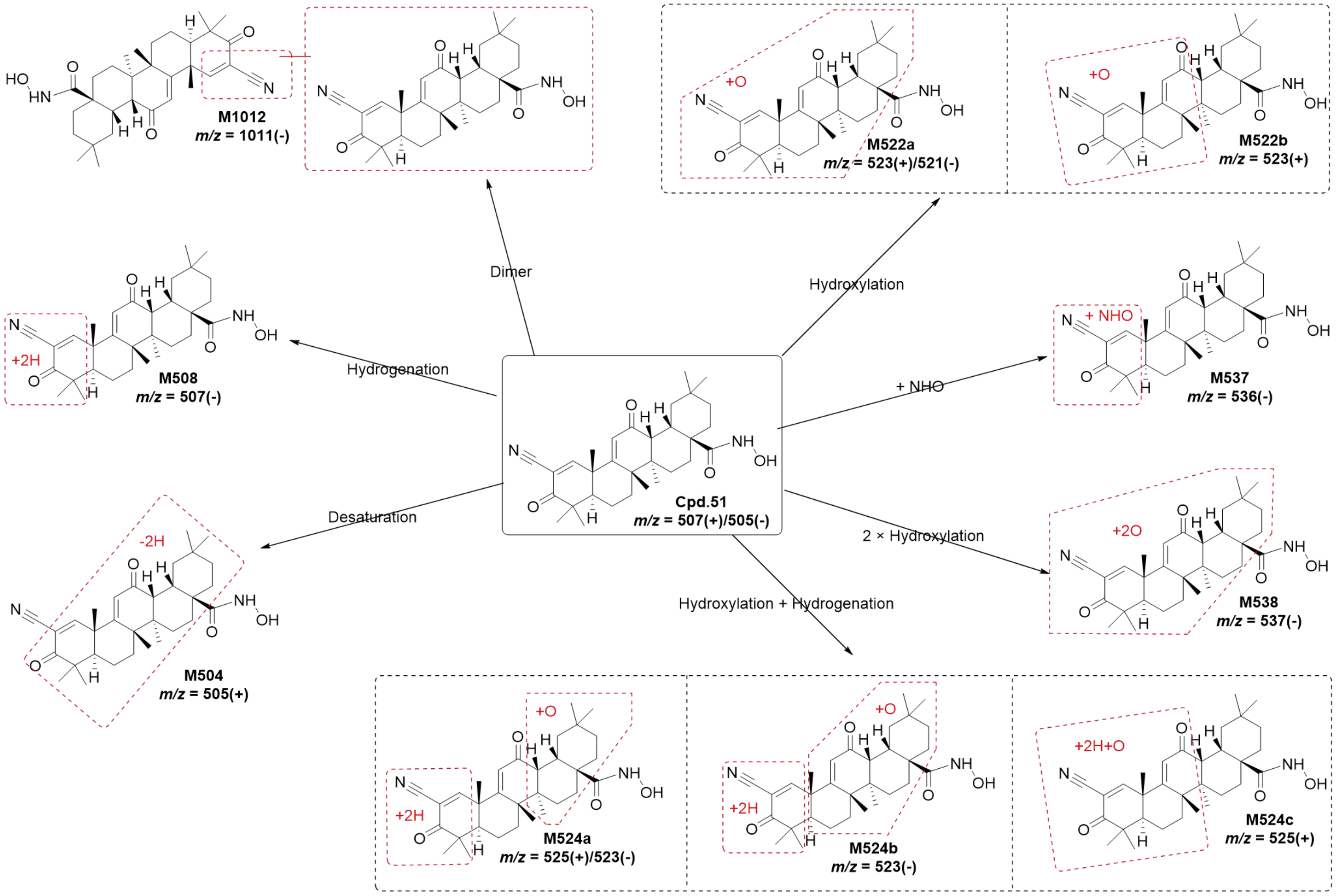

Table 1. Major metabolites of Cpd.51 incubated in human liver microsomes in the presence of NADPH

Peak ID	Found *m/z*	Mass Shift	Biotransformation	R.T. (min)	Human liver microsome	
					Relative UV*	MS peak area
Parent (T_0)	507.3223 $[M+H]^+$	n/a	n/a	5.60	100.00%	1.19E+04
	505.3066 $[M-H]^-$					1.59E+04
Parent (T_5)	507.3223 $[M+H]^+$	n/a	n/a	5.60	54.06%	6.69E+03
	505.3066 $[M-H]^-$					8.97E+03
M504	505.3064 $[M+H]^+$	-2.0157	Desaturation	5.91	1.09%	1.11E+02
	503.2909 $[M-H]^-$					ND
M508	509.3382 $[M+H]^+$	2.0156	Hydrogenation	5.32	11.61%	2.40E+03
	507.3222 $[M-H]^-$					2.59E+03
M522a	523.3130 $[M+H]^+$	15.9933	Hydroxylation	4.33	+	6.00E+01
	521.2999 $[M-H]^-$					1.08E+02
M522b	523.3158 $[M+H]^+$	15.9968	Hydroxylation	4.90	8.13%	7.86E+02
	521.3034 $[M-H]^-$					9.78E+02
M524a	525.3320 $[M+H]^+$	18.0100	Hydroxylation + Hydrogenation	3.96	+	3.90E+01
	523.3166 $[M-H]^-$					5.00E+01
M524b	525.3320 $[M+H]^+$	18.0064	Hydroxylation + Hydrogenation	4.29	+	1.00E+01
	523.3130 $[M-H]^-$					1.20E+01
M524c	525.3319 $[M+H]^+$	18.0089	Hydroxylation + Hydrogenation	5.09	+	5.90E+01
	523.3155 $[M-H]^-$					1.00E+02
M537	538.3284 $[M+H]^+$	31.0069	+ NHO	4.85	+	5.50E+01
	536.3135 $[M-H]^-$					4.30E+01
M538	539.3121 $[M+H]^+$	31.9907	2 × Hydroxylation	3.49	0.69%	2.50E+01
	537.2973 $[M-H]^-$					4.10E+01
M1012	1013.6331 $[M+H]^+$	506.3124	Dimer	7.83	5.16%	7.87E+02
	1011.6190 $[M-H]^-$					8.54E+02

*: All percentages were calculated based on the detected UV (λ = 250-260 nm) absorption relative to that of parent in T_0 sample (normalized as 100%);
+: Only detected in MS;
n/a: Not applicable.

RESULTS AND DISCUSSION

Structure elucidation of Cpd.51 and its metabolites

1) MS fragmentation assignment of Cpd.51

MSMS spectrum was obtained for Cpd.51 and the fragments were assigned to facilitate structure elucidation of Cpd.51 metabolites.

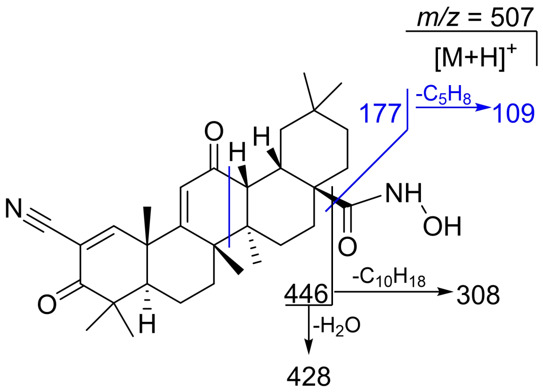

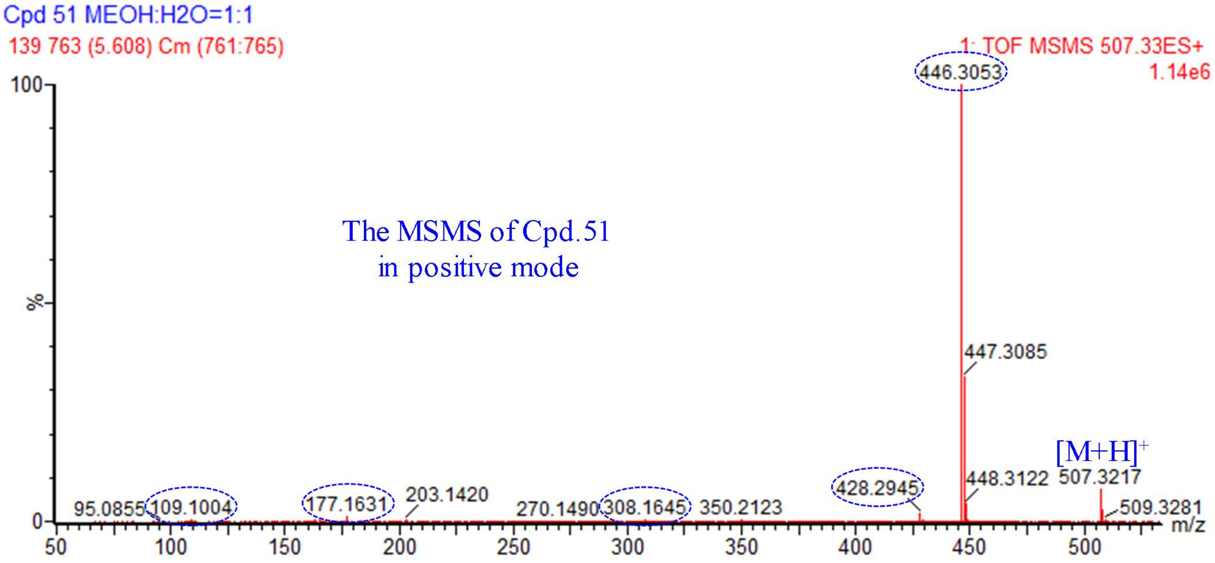

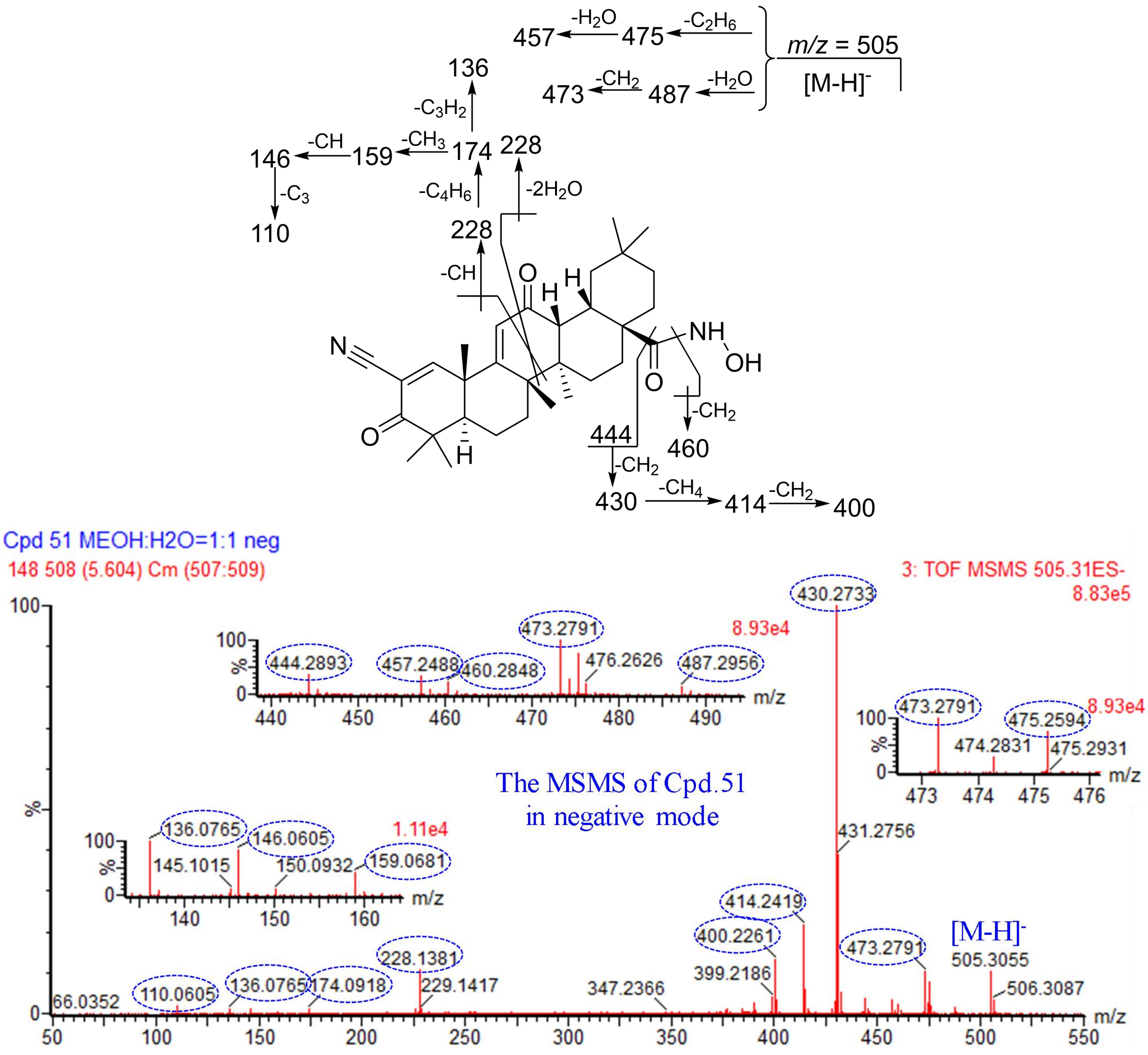

457 -H2O 475 -C2H6
m/z = 505
[M-H]-
473 -CH2 487 -H2O
136
-C3H2
146 -CH 159 -CH3 174 228
-C3
-C4H6
-2H2O
110
228
-CH
N
O
H
H
NH
OH
O
O
H
-CH2
444
460
-CH2
430 -CH4 414 -CH2 400
Cpd 51 MEOH:H2O=1:1 neg
148 508 (5.604) Cm (507:509)
3: TOF MSMS 505.31ES-
8.83e5
430.2733
473.2791
8.93e4
444.2893
457.2488
460.2848
476.2626
487.2956
m/z
473.2791
475.2594
8.93e4
474.2831
475.2931
The MSMS of Cpd.51
in negative mode
136.0765
146.0605
1.11e4
145.1015
150.0932
159.0681
431.2756
414.2419
400.2261
473.2791
[M-H]-
505.3055
506.3087
399.2186
228.1381
229.1417
347.2366
66.0352
110.0605
136.0765
174.0918
%
m/z

2) M504 (Desaturation)

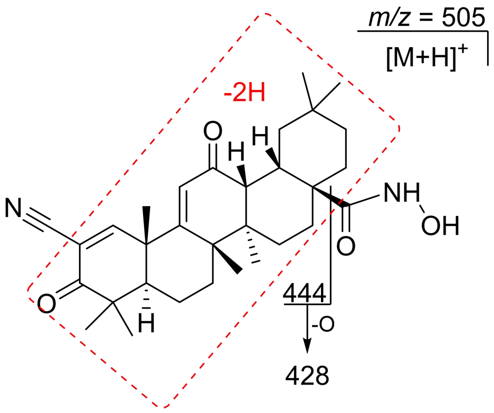

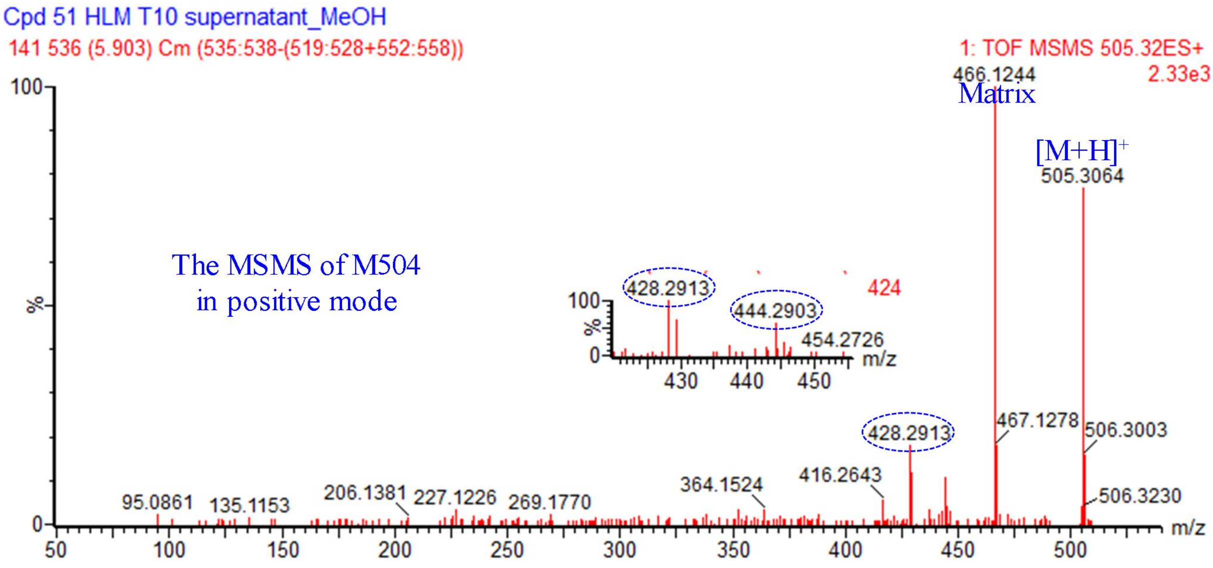

3) M508 (Hydrogenation)

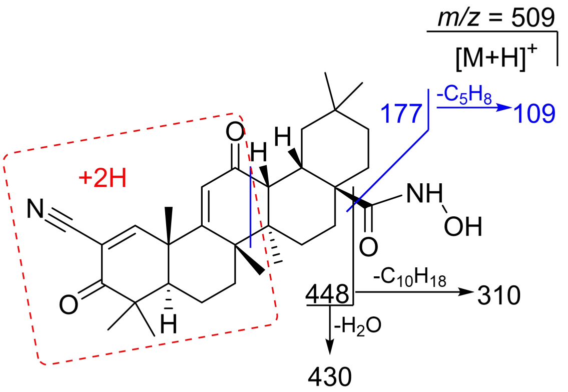

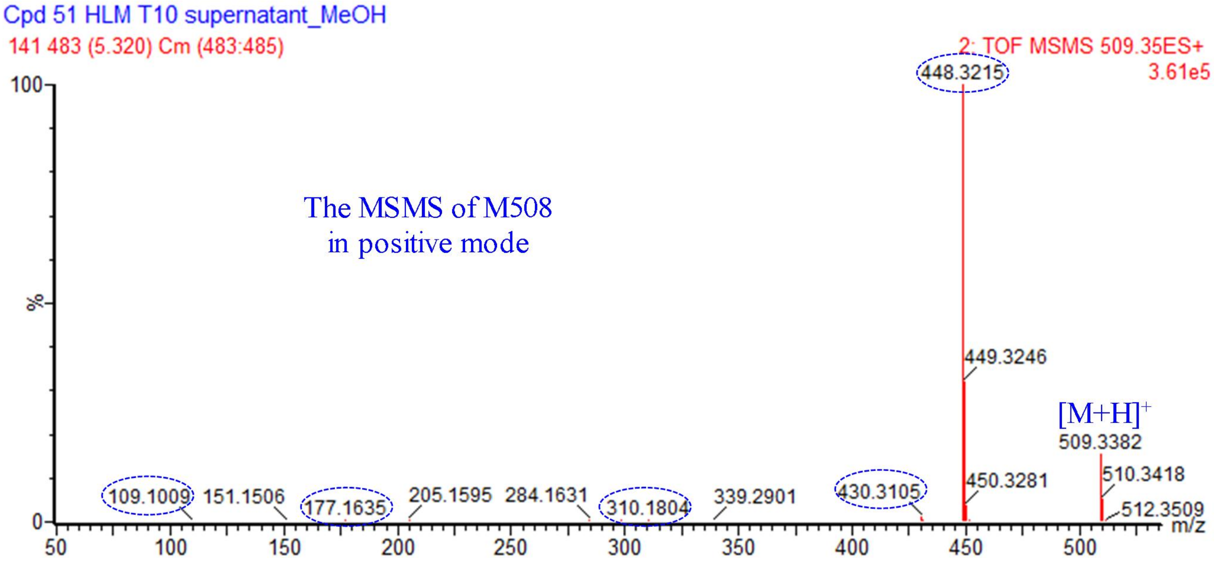

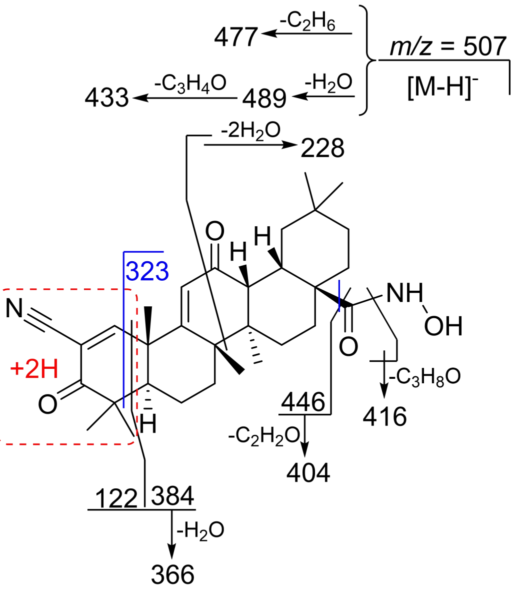

477
$-C_2H_6$
m/z = 507
[M-H]-
433
$-C_3H_4O$
489
$-H_2O$
$-2H_2O$
228
323
+2H
446
$-C_2H_2O$
404
$-C_3H_8O$
416
122
384
$-H_2O$
366

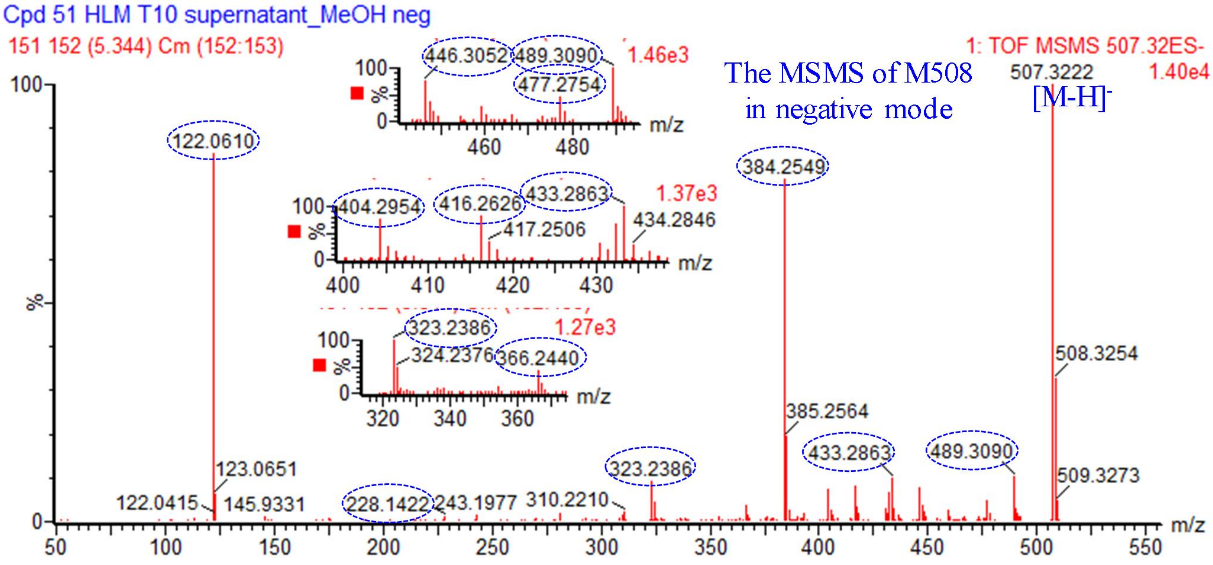

Cpd 51 HLM T10 supernatant_MeOH neg
151 152 (5.344) Cm (152:153)
1: TOF MSMS 507.32ES-
1.40e4
The MSMS of M508 in negative mode
507.3222
[M-H]-
122.0610
384.2549
508.3254
385.2564
123.0651
122.0415
145.9331
228.1422
243.1977
310.2210
323.2386
433.2863
489.3090
509.3273
446.3052
489.3090
477.2754
1.46e3
404.2954
416.2626
433.2863
417.2506
434.2846
1.37e3
323.2386
324.2376
366.2440
1.27e3
m/z
%

4) M522a (Hydroxylation)

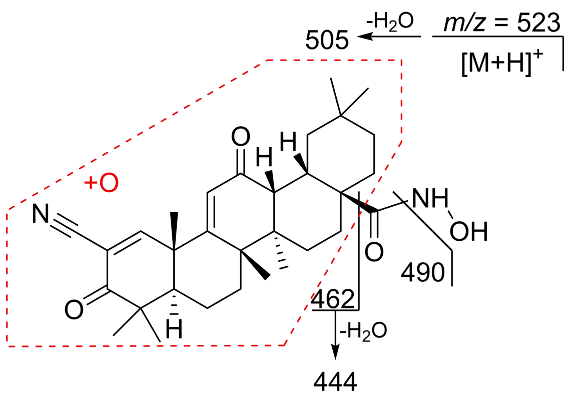

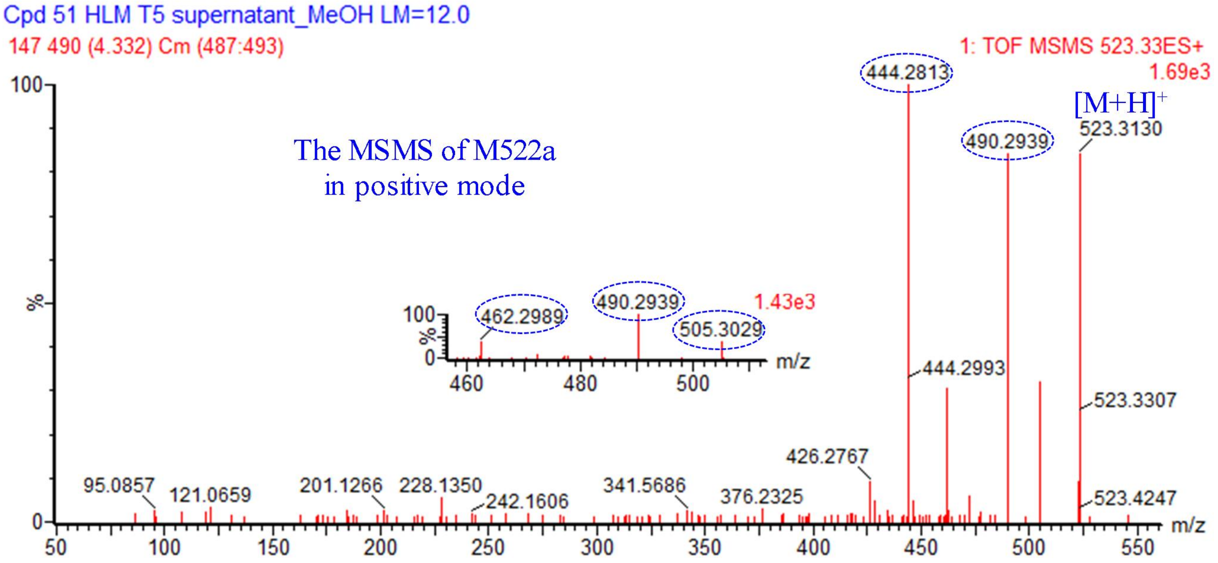

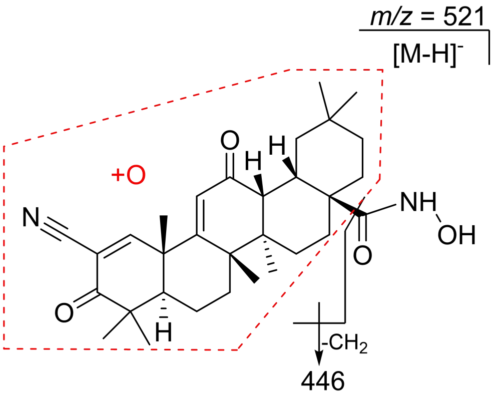
m/z = 521
[M-H]-
+O
-CH2
446

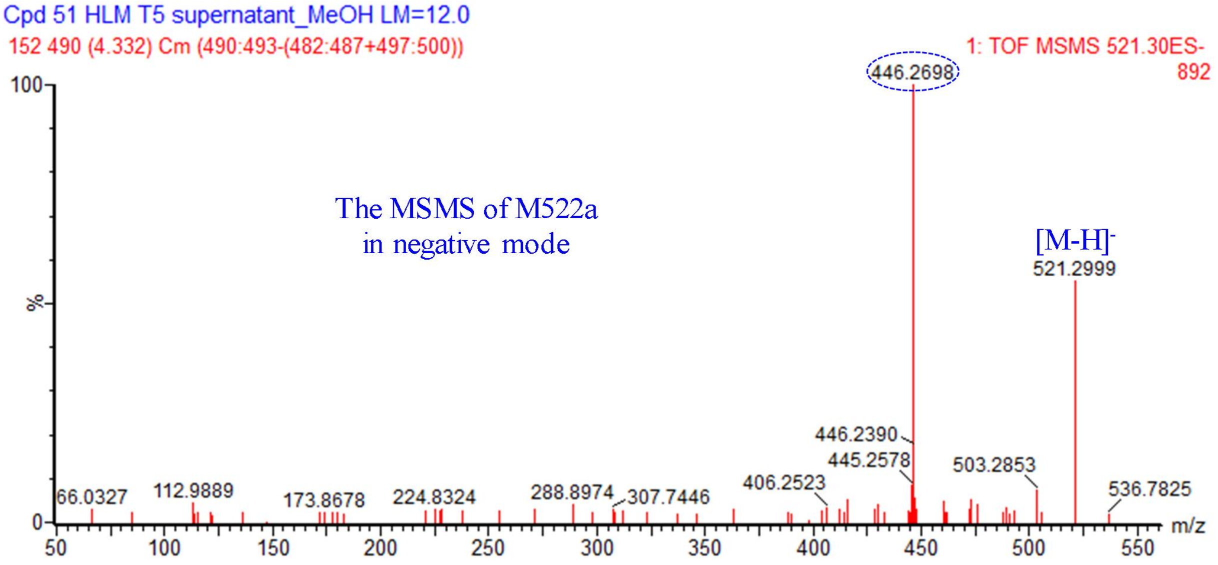
Cpd 51 HLM T5 supernatant_MeOH LM=12.0
152 490 (4.332) Cm (490:493-(482:487+497:500))
1: TOF MSMS 521.30ES-
892
The MSMS of M522a
in negative mode
[M-H]-
521.2999
446.2698
446.2390
445.2578
503.2853
536.7825
406.2523
307.7446
288.8974
224.8324
173.8678
112.9889
66.0327
m/z
%

5) M522b (Hydroxylation)

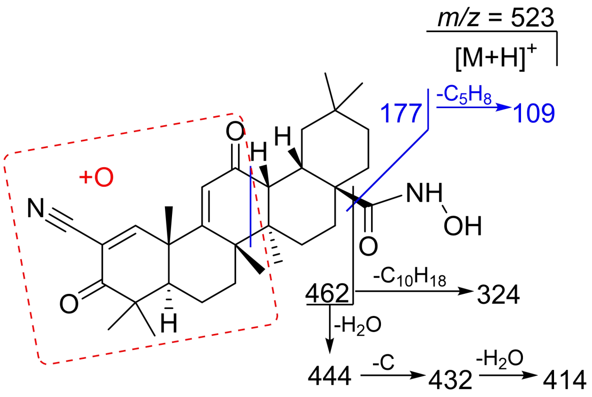

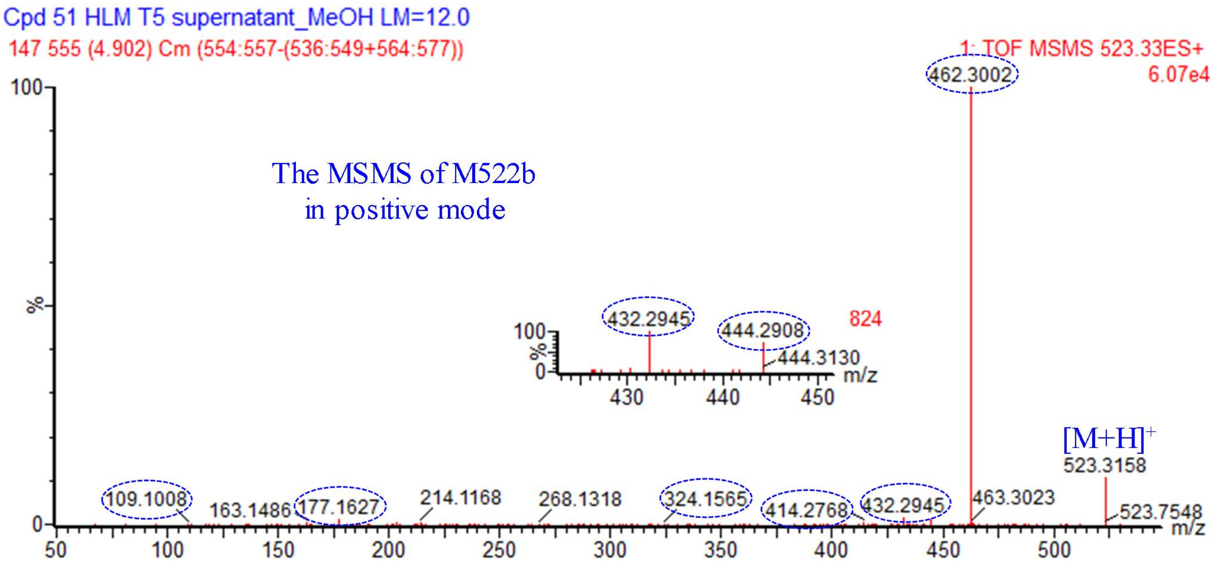

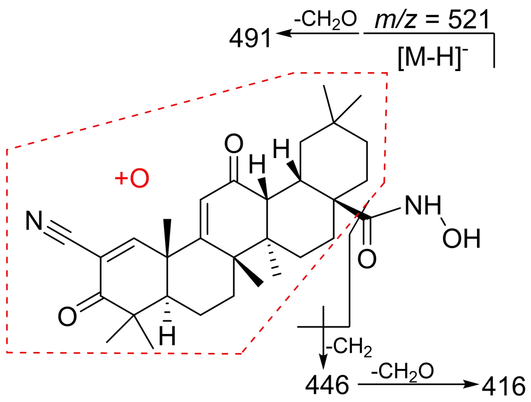
491
-CH2O
m/z = 521
[M-H]-
+O
N
O
H
H
NH
OH
O
O
H
-CH2
446
-CH2O
416

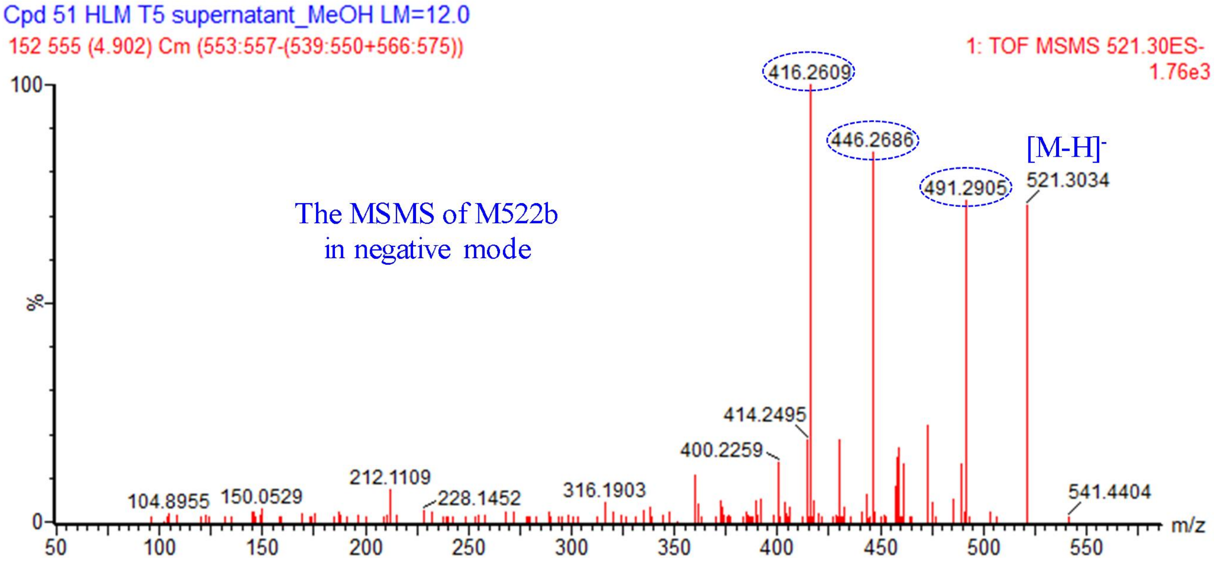
Cpd 51 HLM T5 supernatant_MeOH LM=12.0
152 555 (4.902) Cm (553:557-(539:550+566:575))
1: TOF MSMS 521.30ES-
1.76e3
416.2609
446.2686
491.2905
[M-H]-
521.3034
The MSMS of M522b
in negative mode
414.2495
400.2259
212.1109
104.8955
150.0529
228.1452
316.1903
541.4404
%
m/z

6) M524a (Hydroxylation + Hydrogenation)

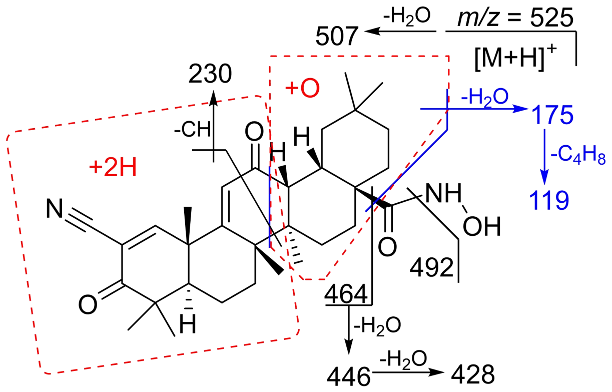

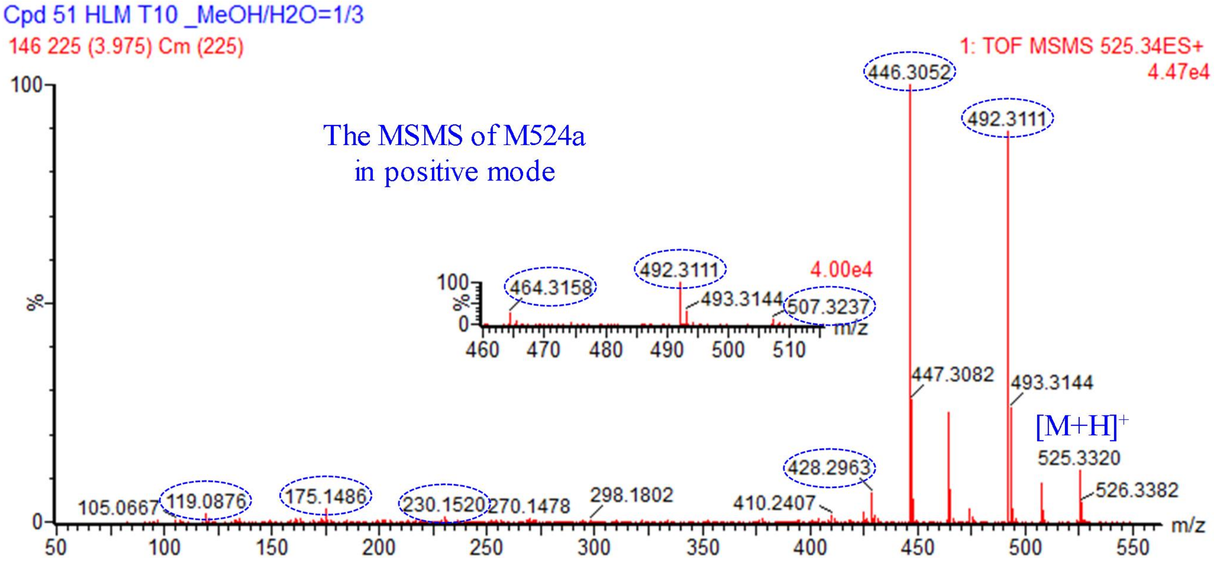

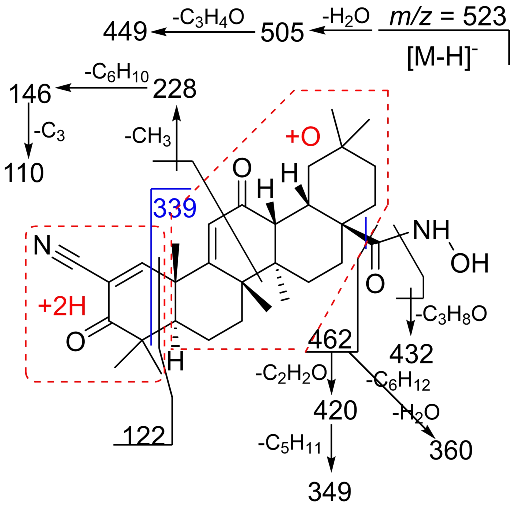
449
-C3H4O
505
-H2O
m/z = 523
[M-H]-
146
-C6H10
228
-C3
110
-CH3
+O
339
N
O
H
H
NH
OH
+2H
O
H
O
462
432
-C3H8O
-C2H2O
420
-C6H12
-H2O
360
122
-C5H11
349

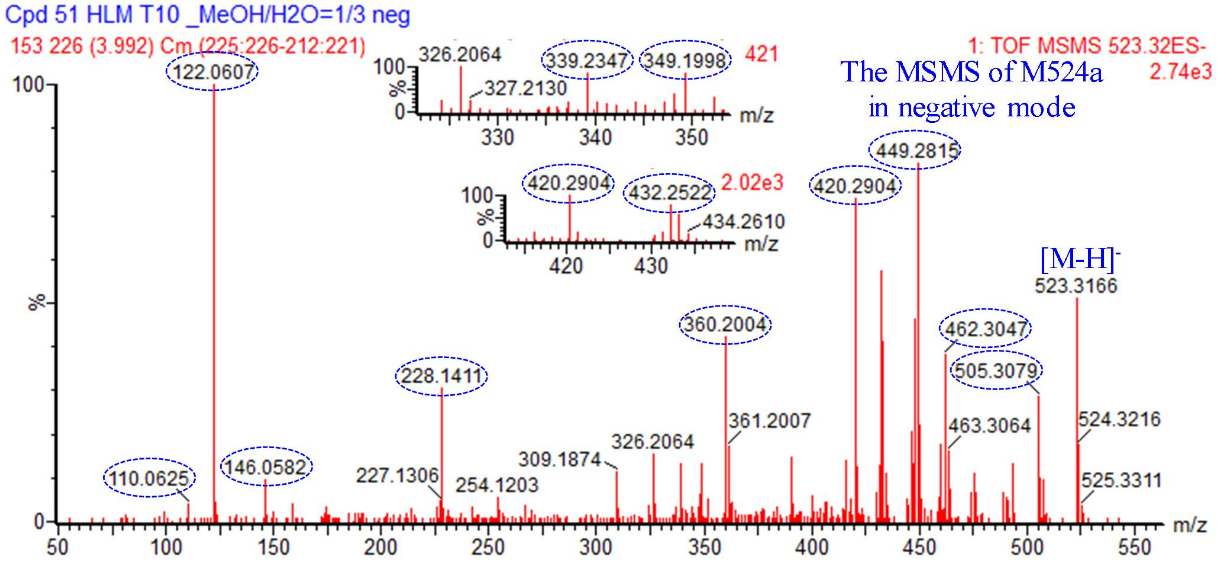
Cpd 51 HLM T10 _MeOH/H2O=1/3 neg
153 226 (3.992) Cm (225:226-212:221)
1: TOF MSMS 523.32ES-
2.74e3
The MSMS of M524a
in negative mode
122.0607
326.2064
339.2347
349.1998
421
327.2130
420.2904
432.2522
2.02e3
434.2610
449.2815
420.2904
[M-H]-
523.3166
360.2004
462.3047
505.3079
228.1411
361.2007
463.3064
524.3216
326.2064
309.1874
110.0625
146.0582
227.1306
254.1203
525.3311
m/z
%

7) M524b (Hydroxylation + Hydrogenation)

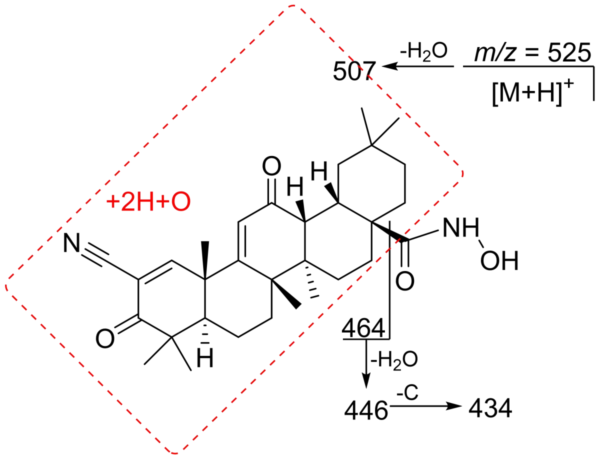

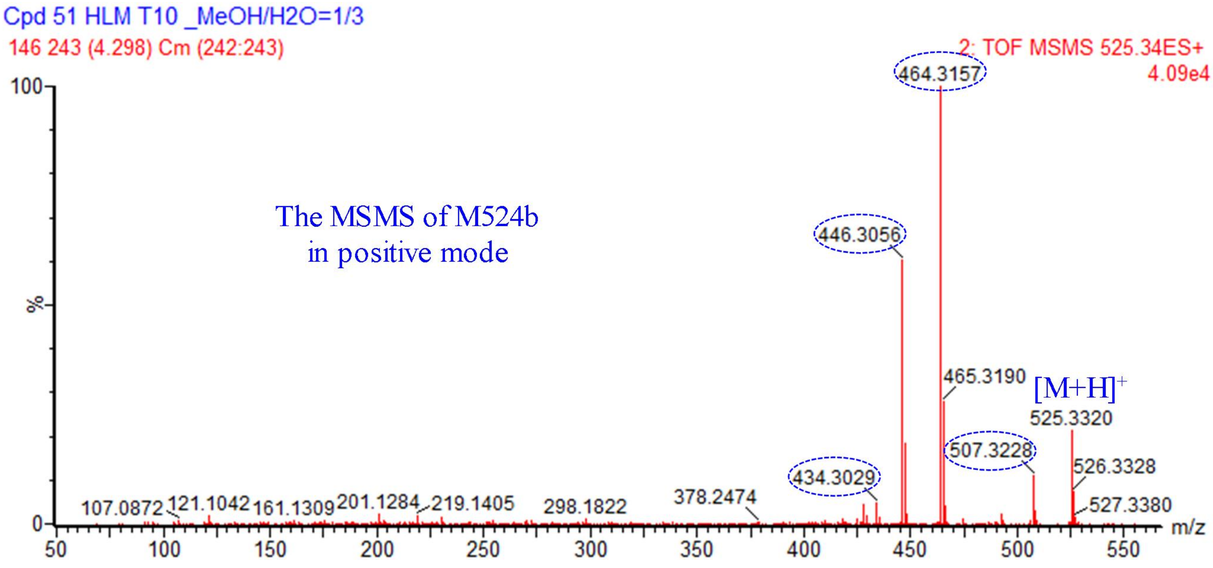

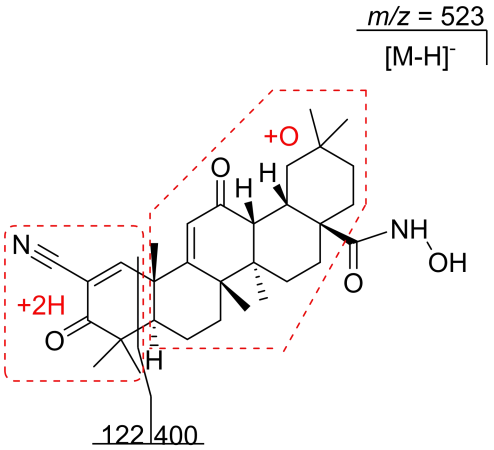
m/z = 523
[M-H]-
+O
+2H
N
O
H
NH
OH
122
400

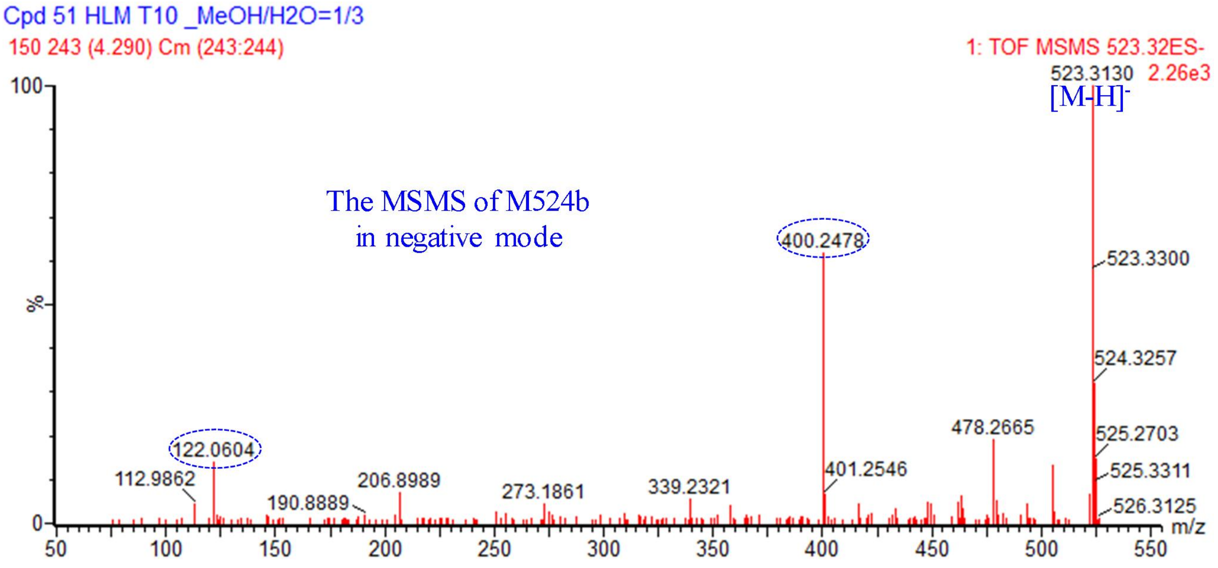
Cpd 51 HLM T10 _MeOH/H2O=1/3
150 243 (4.290) Cm (243:244)
1: TOF MSMS 523.32ES-
523.3130 2.26e3
[M-H]-
The MSMS of M524b
in negative mode
400.2478
523.3300
524.3257
478.2665
525.2703
401.2546
525.3311
526.3125
122.0604
112.9862
206.8989
190.8889
273.1861
339.2321
%
m/z

8) M524c (Hydroxylation + Hydrogenation)

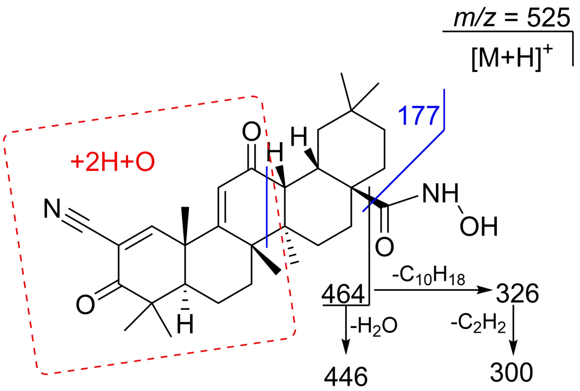

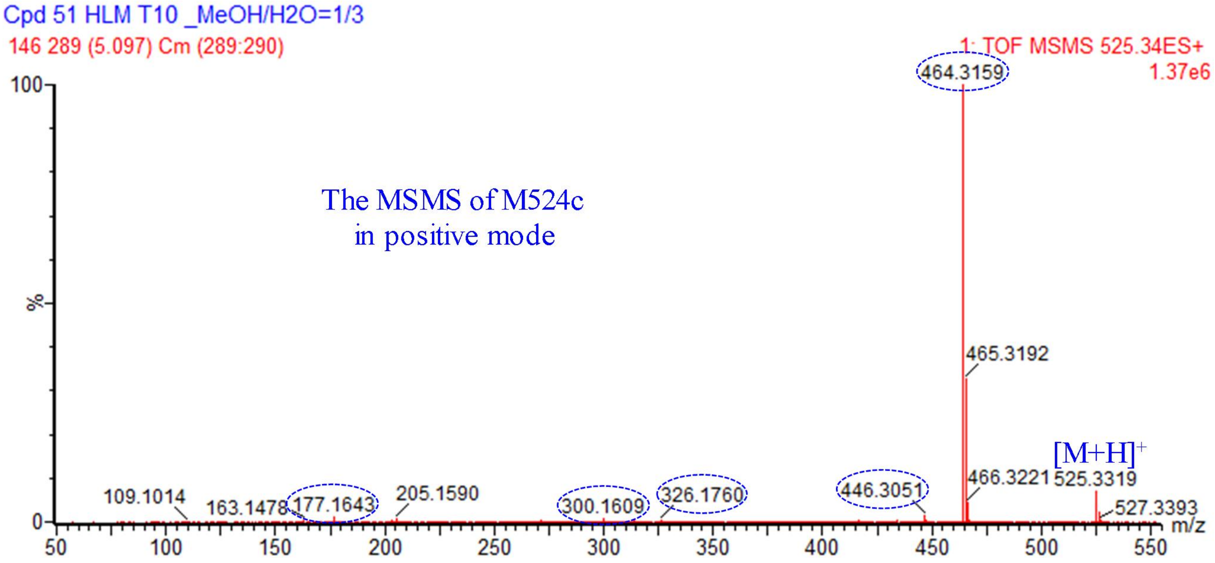

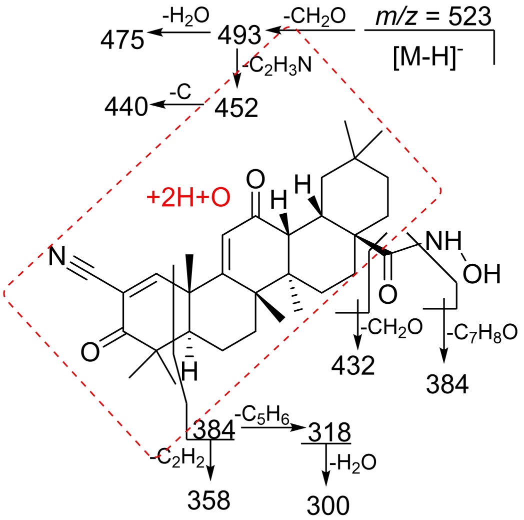

-H2O
-CH2O
m/z = 523
[M-H]-
475
493
-C2H3N
-C
440
452
+2H+O
O
H
H
N
NH
OH
O
O
H
-CH2O
-C7H8O
432
384
384
-C5H6
318
-C2H2
-H2O
358
300

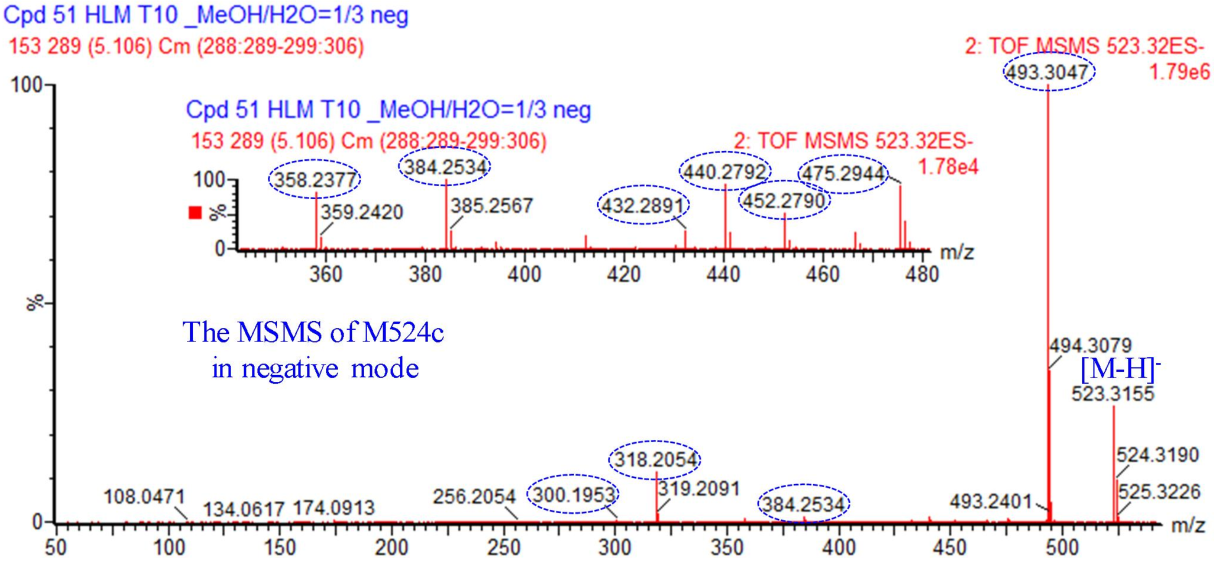

Cpd 51 HLM T10 _MeOH/H2O=1/3 neg
153 289 (5.106) Cm (288:289-299:306)
2: TOF MSMS 523.32ES-
493.3047
1.79e6
100
%
0
Cpd 51 HLM T10 _MeOH/H2O=1/3 neg
153 289 (5.106) Cm (288:289-299:306)
2: TOF MSMS 523.32ES-
1.78e4
358.2377
384.2534
440.2792
475.2944
359.2420
385.2567
432.2891
452.2790
360
380
400
420
440
460
480
m/z
The MSMS of M524c
in negative mode
494.3079
[M-H]-
523.3155
524.3190
525.3226
108.0471
134.0617
174.0913
256.2054
300.1953
318.2054
319.2091
384.2534
493.2401
50
100
150
200
250
300
350
400
450
500
m/z

9) M537 (+ NHO)

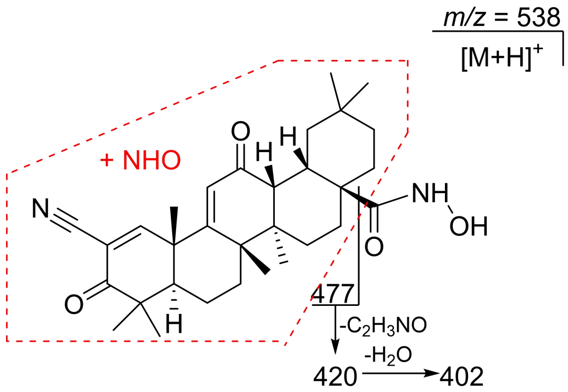
m/z = 538
[M+H]+
+ NHO
477
-C2H3NO
-H2O
420
402

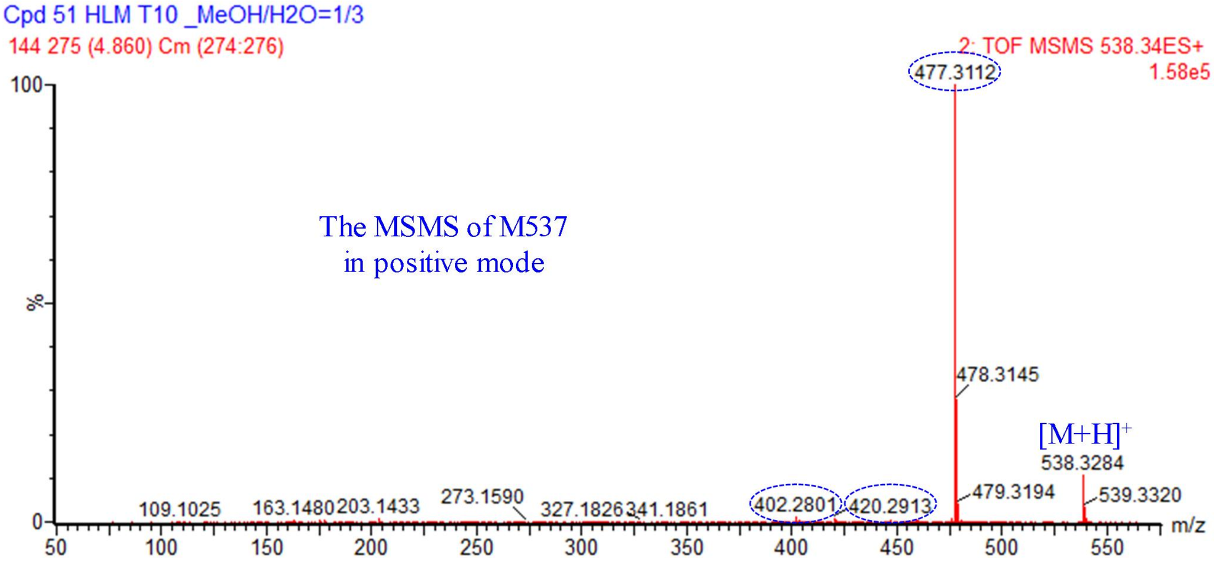
Cpd 51 HLM T10 _MeOH/H2O=1/3
144 275 (4.860) Cm (274:276)
2: TOF MSMS 538.34ES+
1.58e5
477.3112
The MSMS of M537
in positive mode
478.3145
[M+H]+
538.3284
109.1025
163.1480
203.1433
273.1590
327.1826
341.1861
402.2801
420.2913
479.3194
539.3320
m/z
%

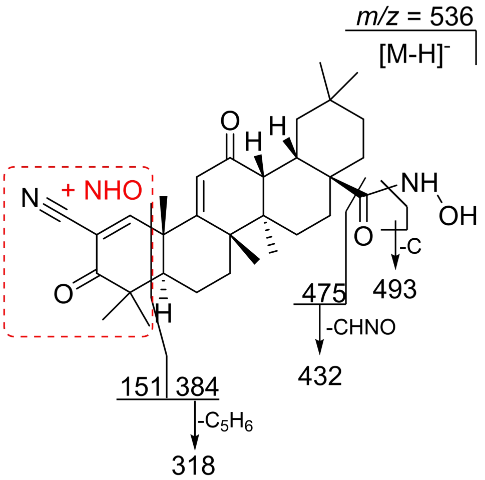
m/z = 536
[M-H]-
+ NHO
N
O
H
NH
OH
-C
493
475
-CHNO
432
151
384
-C5H6
318

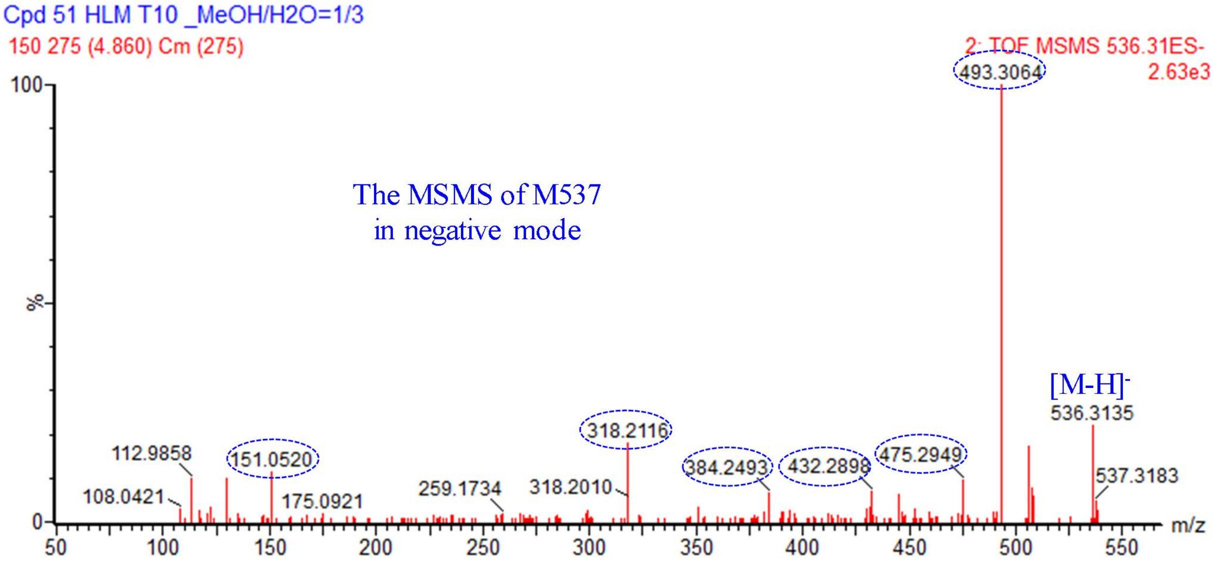
Cpd 51 HLM T10 _MeOH/H2O=1/3
150 275 (4.860) Cm (275)
2: TOF MSMS 536.31ES-
2.63e3
The MSMS of M537
in negative mode
493.3064
[M-H]-
536.3135
537.3183
112.9858
151.0520
108.0421
175.0921
259.1734
318.2116
318.2010
384.2493
432.2898
475.2949
%
m/z

10) M538 (2 × Hydroxylation)

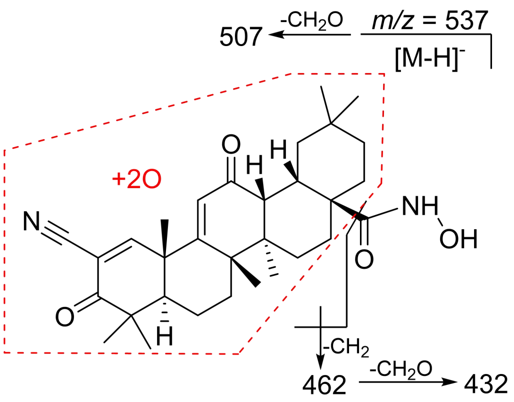

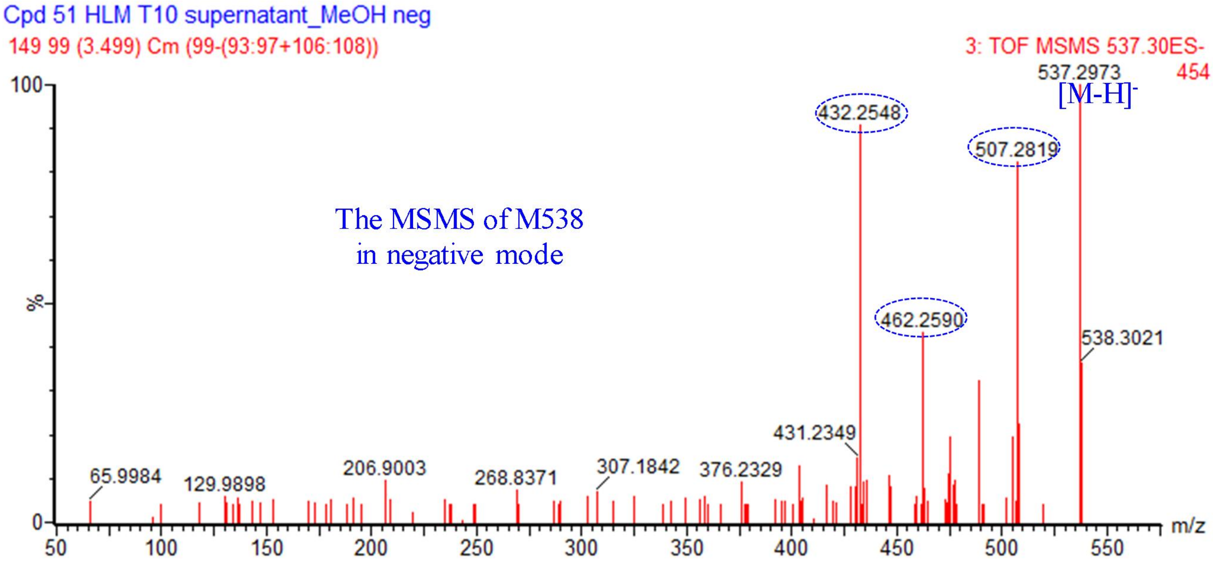

11) M1012 (Dimer)

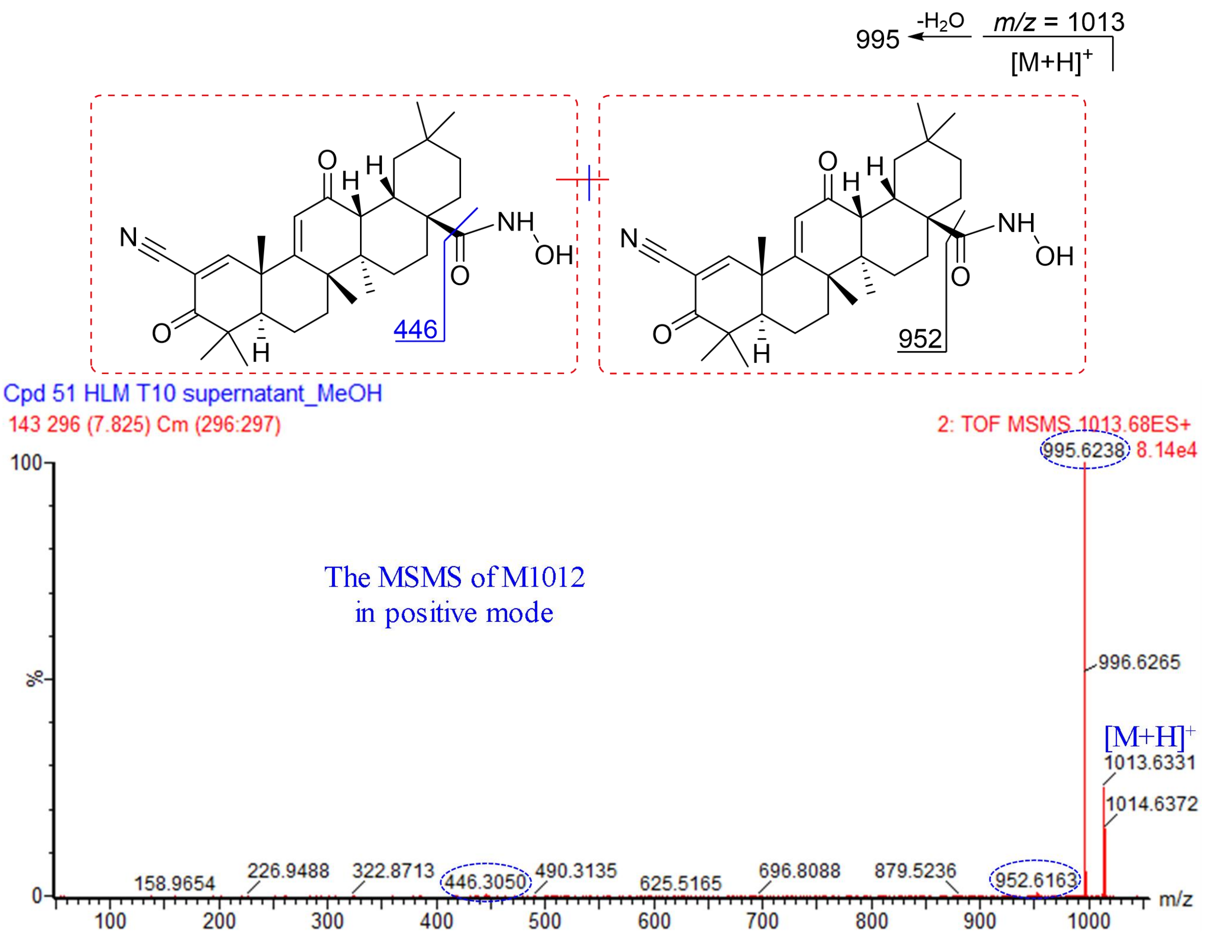

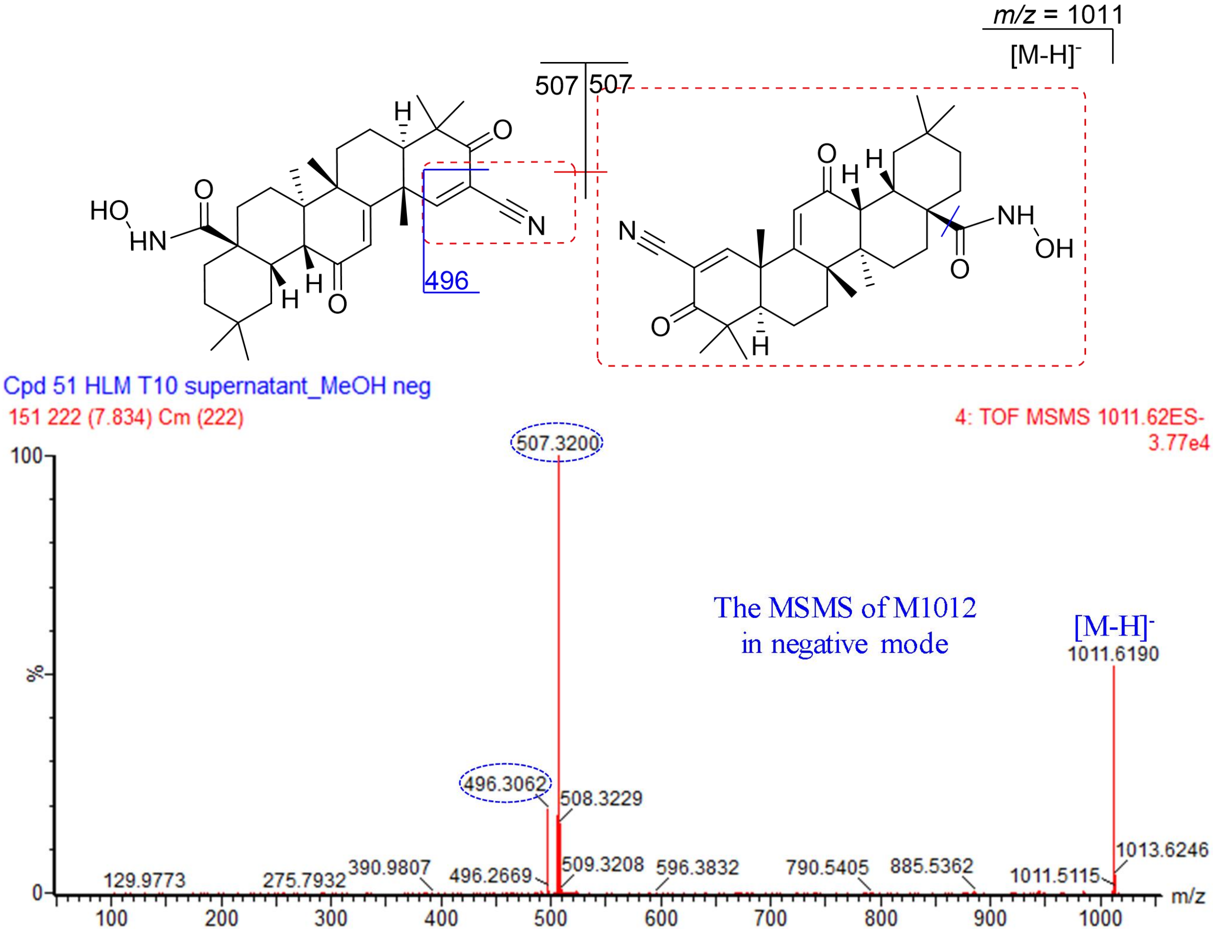

Supplement: Supplementary file 2 — Supplementary Document Part II. [file thnov16p5713s2.pdf]
